# Supplementary material for: Characterising the asynchronous resurgence of common respiratory viruses following the COVID-19 pandemic
Source: Nat Commun. 2025 Feb 13;16:1610. doi: 10.1038/s41467-025-56776-z (PMC11825952; doi:10.1038/s41467-025-56776-z)
Supplement: Supplementary file 1 — Supplementary Information [file 41467_2025_56776_MOESM1_ESM.pdf]

**Supplementary Materials**

# Characterising the asynchronous resurgence of common respiratory viruses following the onset of the COVID-19 pandemic

Chenkai Zhao, Tiantian Zhang, Ling Guo, Shiqi Sun, Yumeng Miao, Chee Fu Yung, Jane Tomlinson, Kirill Stolyarov, Zakhar Shchomak, Yong Poovorawan, David James Nokes, Carmen Muñoz-Almagro, Michal Mandelboim, James W Keck, Joanne Marie Langley, Terho Heikkinen, Jikui Deng, Philippe Colson, Giorgi Chakhunashvili, Mauricio T. Caballero, Louis Bont, Daniel R Feikin, Harish Nair, Xin Wang, You Li & Respiratory Virus Global Epidemiology Network

# Table of Contents

*Supplementary results* ............................................................ *1*

**Supplementary Table 1. Description of included studies.** ............................................................ **1**

**Supplementary Table 2. Description of included surveillance reports.** ............................................................ **3**

**Supplementary Table 3. Description of included data from RSV GEN44.** ............................................................ **4**

**Supplementary Table 4. Quality assessment results of included studies from the systematic literature search.** **5**

**Supplementary Table 5. Quality assessment results of included surveillance reports.** ............... **6**

**Supplementary Table 6. Quality assessment results of data from RSV GEN.** ............................ **10**

**Supplementary Figure 1. Distribution of common respiratory virus activity in this study.** ..... **11**

**Supplementary Figure 2. The virus-specific time interval (with 95% CI) between the peaks in two subsequent years in temperate regions.** ............................................................ **12**

**Supplementary Figure 3. The virus-specific time interval (with 95% CI) between the peaks in two subsequent years in tropics regions.** ............................................................ **13**

**Supplementary Figure 4. The time difference in days of onset between viruses during their first resurgence and second resurgence following the COVID-19 pandemic onset, matched by sites reporting influenza A virus subtypes.** ............................................................ **14**

**Supplementary Figure 5. The time difference in days of peak between viruses during their first resurgence and second resurgence following the COVID-19 pandemic onset, matched by sites reporting influenza A virus subtypes.** ............................................................ **15**

**Supplementary Figure 6. The time difference in days of onset between viruses during their first resurgence and second resurgence following the COVID-19 pandemic onset, matched by sites reporting PIV subtypes.** ............................................................ **16**

**Supplementary Figure 7. The time difference in days of peak between viruses during their first resurgence and second resurgence following the COVID-19 pandemic onset, matched by sites reporting PIV subtypes.** ............................................................ **17**

**Supplementary Figure 8. The time difference in days of peak between viruses during their first resurgence and second resurgence following the COVID-19 pandemic onset, matched by study sites in the temperate region.** ............................................................ **18**

**Supplementary Figure 9. The time difference in days of peak between viruses during their first resurgence and second resurgence following the COVID-19 pandemic onset, matched by study sites in the tropical region.** ............................................................ **19**

**Supplementary Figure 10. The time difference in days of onset between viruses during their first resurgence and second resurgence following the COVID-19 pandemic onset, matched by study sites in the northern temperate region.** ........................................................................ **20**

**Supplementary Figure 11. The time difference in days of peak between viruses during their first resurgence and second resurgence following the COVID-19 pandemic onset, matched by study sites in the northern temperate region.** ........................................................................ **21**

**Supplementary Figure 12. The time difference in days of onset between viruses during their first resurgence and second resurgence following the COVID-19 pandemic onset, matched by study sites in the southern temperate region.** ........................................................................ **22**

**Supplementary Figure 13. The time difference in days of peak between viruses during their first resurgence and second resurgence following the COVID-19 pandemic onset, matched by study sites in the southern temperate region.** ........................................................................ **23**

**Supplementary Figure 14. The time difference in days of onset between viruses during their first resurgence and second resurgence following the COVID-19 pandemic onset, matched by study sites in high-income countries.** ........................................................................ **24**

**Supplementary Figure 15. The time difference in days of peak between viruses during their first resurgence and second resurgence following the COVID-19 pandemic onset, matched by study sites in high-income countries.** ........................................................................ **25**

**Supplementary Figure 16. The time difference in days of onset between viruses during their first resurgence and second resurgence following the COVID-19 pandemic onset, matched by sites in non-high-income countries** ........................................................................ **26**

**Supplementary Figure 17. The time difference in days of peak between viruses during their first resurgence and second resurgence following the COVID-19 pandemic onset, matched by study sites in non-high-income countries.** ........................................................................ **27**

*Sensitivity analysis result* ........................................................................ *28*

**Supplementary Figure 18. The time difference in days of onset between viruses during their first resurgence and second resurgence following the COVID-19 pandemic onset, matched by study sites reporting positive proportion data.** ........................................................................ **28**

**Supplementary Figure 19. The time difference in days of peak between viruses during their first resurgence and second resurgence following the COVID-19 pandemic onset, matched by study sites reporting positive proportion data.** ........................................................................ **29**

**Supplementary Figure 20. The time difference in days of onset between viruses during their first resurgence and second resurgence following the COVID-19 pandemic onset, matched by study sites reporting positive cases data.** ........................................................................ **30**

**Supplementary Figure 21. The time difference in days of peak between viruses during their first resurgence and second resurgence following the COVID-19 pandemic onset, matched by study sites reporting positive cases data.** ........................................................................ **31**

**Supplementary Figure 22. The time difference in days of onset between viruses during their first resurgence and second resurgence following the COVID-19 pandemic onset, matched by study sites using data extracted from systematic literature review.................................................32**

**Supplementary Figure 23. The time difference in days of peak between viruses during their first resurgence and second resurgence following the COVID-19 pandemic onset, matched by study sites using data extracted from systematic literature review.................................................33**

**Supplementary Figure 24. The time difference in days of onset between viruses during their first resurgence and second resurgence following the COVID-19 pandemic onset, matched by study sites using data extracted from databases...............................................................................34**

**Supplementary Figure 25. The time difference in days of peak between viruses during their first resurgence and second resurgence following the COVID-19 pandemic onset, matched by study sites using data extracted from databases...............................................................................35**

**Supplementary Figure 26. The time difference in days of onset between viruses during their first resurgence and second resurgence following the COVID-19 pandemic onset, matched by study sites reporting monthly aggregated viral activity data............................................................36**

**Supplementary Figure 27. The time difference in days of peak between viruses during their first resurgence and second resurgence following the COVID-19 pandemic onset, matched by study sites reporting monthly aggregated viral activity data............................................................37**

**Supplementary Figure 28. The time difference in days of onset between viruses during their first resurgence and second resurgence following the COVID-19 pandemic onset, matched by study sites reporting weekly aggregated viral activity data. ............................................................38**

**Supplementary Figure 29. The time difference in days of peak between viruses during their first resurgence and second resurgence following the COVID-19 pandemic onset, matched by study sites reporting weekly aggregated viral activity data. ............................................................39**

**Supplementary Figure 30. The time difference in days of onset between viruses during their first resurgence and second resurgence following the COVID-19 pandemic onset, matched by study sites providing moderate-to-high quality data. .........................................................................40**

**Supplementary Figure 31. The time difference in days of peak between viruses during their first resurgence and second resurgence following the COVID-19 pandemic onset, matched by study sites providing moderate-to-high quality data. .........................................................................41**

**Supplementary Figure 32. The time difference in days of onset between viruses during their first resurgence and second resurgence following the COVID-19 pandemic onset, matched by study sites providing all age group data...............................................................................................42**

**Supplementary Figure 33. The time difference in days of peak between viruses during their first resurgence and second resurgence following the COVID-19 pandemic onset, matched by study sites providing all age group data...............................................................................................43**

*Ad-hoc analysis results* ........................................................................................................ *44*

**Supplementary Figure 34. The time difference in days of onset between viruses during their first resurgence and second resurgence following the COVID-19 pandemic onset in study sites excluding Canadian data. 44**

**Supplementary Figure 35. The time difference in days of peak between viruses during their first resurgence and second resurgence following the COVID-19 pandemic onset in study sites excluding Canadian data. 45**

**Supplementary Figure 36. The time difference in days of onset between viruses during their first resurgence and second resurgence following the COVID-19 pandemic onset in study sites excluding Chinese data. 46**

**Supplementary Figure 37. The time difference in days of peak between viruses during their first resurgence and second resurgence following the COVID-19 pandemic onset in study sites excluding Chinese data. 47**

**Supplementary Figure 38. The time difference in days of onset between viruses during their first resurgence and second resurgence following the COVID-19 pandemic onset in study sites excluding Finnish data. 48**

**Supplementary Figure 39. The time difference in days of peak between viruses during their first resurgence and second resurgence following the COVID-19 pandemic onset in study sites excluding Finnish data. 49**

**Supplementary Figure 40. The time difference in days of onset between viruses during their first resurgence and second resurgence following the COVID-19 pandemic onset, using nationally aggregated data from Canada and Finland. .................................................................. 50**

**Supplementary Figure 41. The time difference in days of peak between viruses during their first resurgence and second resurgence following the COVID-19 pandemic onset, using nationally aggregated data from Canada and Finland. .................................................................. 51**

**Supplementary Figure 42. The virus-specific time interval (with 95% CI) between the onsets in two subsequent years across national (A), provincial (B), and municipal (C) levels in Canada. 52**

**Supplementary Figure 43. The virus-specific time interval (with 95% CI) between the peaks in two subsequent years across national (A), provincial (B), and municipal (C) levels in Canada.... 53**

**Supplementary Figure 44. The time difference in days of onset between viruses during their first resurgence and second resurgence following the COVID-19 pandemic onset across national (A), provincial (B), and municipal (C) levels in Canada. ................................................................. 54**

**Supplementary Figure 45. The time difference in days of peak between viruses during their first resurgence and second resurgence following the COVID-19 pandemic onset across national (A), provincial (B), and municipal (C) levels in Canada. ................................................................. 55**

**Supplementary Figure 46. The time difference in days of onset between viruses during their first resurgence and second resurgence following the COVID-19 pandemic, matched by study sites that had over 1000 positive cases for each virus. ......56**

**Supplementary Figure 47. The time difference in days of peak between viruses during their first resurgence and second resurgence following the COVID-19 pandemic, matched by study sites that had over 1000 positive cases for each virus. ......57**

***Supplementary methods ...... 58***

**Supplementary Methods 1. Search Strategy. ......58**

**Supplementary Methods 2. Quality assessment form. ......61**

***Reference ...... 62***

**Supplementary results**

**Supplementary Table 1.Description of included studies**

| Author | Country | Site name | Study period | Reported viruses | Reporting frequency | Indicator(s) |
|---|---|---|---|---|---|---|
| Sheila F Lumley1 | United Kingdom | Oxford | 2016/03-2021/07 | IV, RSV, PIV, MPV, sCoV, RV, AdV | Month | Proportion |
| Yohei Kume2 | Japan | Fukushima | 2018/01-2021/12 | IV, RSV, PIV, MPV, sCoV, RV, AdV | Month | Case |
| Hye Jin Shi3 | Korea, Rep. | Incheon | 2018/01-2021/12 | IV, RSV, PIV, MPV, sCoV, RV, AdV | Month | Proportion |
| Xiucui Han4 | China | Hangzhou | 2019/01-2021/10 | IV, RSV, AdV | Month | Proportion & Case |
| A. A. Sominina5 | Russian | Nationwide | 2015/10-2021/05 | IV, RSV, PIV, MPV, sCoV, RV, AdV | Week | Proportion |
| Pengcheng Liu6 | China | Shanghai | 2018/06-2022/01 | IV, RSV, RV, AdV | Month | Proportion |
| Asmae Lamrani Hanchi7 | Morocco | Marrakech | 2018/01-2021/12 | IV, RSV, RV | Month | Case |
| Chungmin Park8 | Korea, Rep. | Nationwide | 2018/01-2021/04 | IV, RSV, PIV, MPV, sCoV, RV, AdV | Week | Proportion & Case |
| Lu Wan9 | China | Wuhan | 2018/01-2021/12 | IV, RSV, PIV, AdV | Month | Proportion |
| Ron Dagan10 | Israel | Negev | 2016/01-2021/12 | IV, RSV, PIV, MPV, RV, AdV | Month | Case |
| Norosoa Harline Razanajatovo11 | Madagascar | Nationwide | 2020/03-2022/05 | IV, RSV, PIV, MPV, sCoV, RV, AdV | Week | Case |
| Dana Danino12 | Israel | Negev | 2016/01-2022/03 | IV, RSV, PIV, AdV | Month | Case |
| Heping Wang13 | China | Shenzhen | 2018/07-2022/01 | IV, RSV, PIV, MPV, sCoV, RV, AdV | Month | Proportion |
| Ilana S. Fratty14 | Israel | Nationwide | 2019/08-2022/01 | IV, RSV, PIV, MPV, AdV | Week | Case |
| Zaid Haddadin15 | United States | Nashville | 2018/04-2021/07 | IV, RSV, PIV, MPV, sCoV, RV, AdV | Month | Case |
| Yonatan Oster16 | Israel | Jerusalem | 2017/01-2022/06 | IV, RSV, PIV, MPV, AdV | Month | Proportion & Case |
| Ying Li17 | China | Wuhan | 2019/01-2022/12 | IV, RSV, PIV, HADV | Month | Proportion & Case |
| Chuang-Xing Lin18 | China | Chaozhou | 2020/01-2021/12 | IV, RSV, PIV, MPV, RV, AdV | Month | Proportion |
| Yuan Feng19 | China | Xiangtan | 2018/01-2021/12 | IV, RSV, PIV, ADV | Month | Case |
| Nicole Maison20 | Germany | Munich | 2019/01-2022/11 | IV, RSV, PIV, MPV, sCoV, RV, AdV | Month | Case |
| Xue Fu21 | China | Guizhou | 2018/11-2023/05 | IV, RSV, PIV, AdV | Month | Proportion |
| Yuya Fukuda22 | Japan | Hokkaido | 2019/07-2023/06 | IV, RSV, MPV, AdV | Month | Case |
| Rundong Cao23 | China | Xuzhou | 2015/01-2021/12 | IV, RSV, PIV, sCoV,RV, AdV | Month | Case |
| Víctor Guadalupe-Fernández24 | Spain | Catalonia | 2016/06-2021/07 | IV, RSV, PIV, AdV | Week | Case |
| Yingchan Hao25 | China | Wuhan | 2018/01-2022/12 | IV, RSV, PIV, HADV | Month | Case |
| Mingyu Tang26 | China | Shanghai | 2019/01-2020/12 | IV, RSV, PIV, RV, AdV | Month | Proportion |
| Jianbo Xia27 | China | Wuhan | 2017/01-2021/12 | IV, RSV, PIV, AdV | Month | Proportion |
| Michaela Davids28 | South Africa | Gauteng，Mpumalanga | 2018/08-2022/04 | IV, RSV, PIV, MPV, sCoV, RV, AdV | Month | Case |

| | | | | | | |
|---|---|---|---|---|---|---|
| J. Dina29 | France | Normandy | 2016/09-2022/08 | IV, RSV, PIV, MPV, sCoV, RV, AdV | Month | Proportion |
| Füsun Kırca30 | Turkey | Ankara | 2020/04-2022/10 | IV, RSV, PIV, MPV, sCoV, RV, AdV | Month | Case |
| Wai-Sing Chan31 | China | Hong Kong | 2014/01-2023/04 | IV, RSV, PIV, MPV, sCoV, RV, AdV | Month | Proportion |

**Supplementary Table 2. Description of included surveillance reports.**

| Database name | Site | Study period | Reported viruses | Reporting frequency | Indicator(s) |
|---|---|---|---|---|---|
| The National Respiratory and Enteric Virus Surveillance System32 | United States | 2017/07-2024/04 | IV, RSV, PIV, MPV, sCoV, RV, AdV | Week | Proportion |
| Seattle Flu Alliance33 | United States | 2018/11-2022/02 | IV, RSV, PIV, MPV, sCoV, RV, AdV | Week | Proportion & Case |
| Houston methodist-Respiratory Pathogen Epidemiology Snapshot34 | United States | 2019/08-2023/03 | IV, RSV, RV | Week | Proportion & Case |
| Korea Disease Control and Prevention Agency35 | Korea, Rep. | 2017/01-2024/04 | IV, RSV, PIV, MPV, sCoV, RV, AdV | Week | Case |
| Finnish National Infectious Diseases Register.36 | Finland | 2017/01-2023/12 | IV, RSV, PIV, AdV | Month | Case |
| Taiwan National Infectious Disease Statistics System37 | Taiwan, China | 2018/10-2024/04 | IV, RSV, PIV, AdV | Week | Case |
| Czech Republic National institutes of health38 | Czech Republic | 2017/09-2024/04 | IV, RSV, PIV, MPV, sCoV, RV | Week | Case |
| FluWatch Surveillance39 | Canada | 2016/08-2024/04 | IV, RSV, MPV, RV, AdV | Week | Case |
| PAHO–FluNet40 | Bolivia | 2018/01-2023/12 | IV, RSV, PIV, MPV, sCoV, RV, AdV | Week | Case |
| PAHO–FluNet | Paraguay | 2018/01-2023/12 | IV, RSV, PIV, MPV, AdV | Week | Case |
| PAHO–FluNet | Colombia | 2018/01-2023/12 | IV, RSV, PIV, MPV, sCoV, RV, AdV | Week | Case |
| PAHO–FluNet | Argentina | 2018/01-2023/12 | IV, RSV, PIV, MPV, sCoV, AdV | Week | Case |
| PAHO–FluNet | Chile | 2018/01-2023/12 | IV, RSV, PIV, MPV, AdV | Week | Case |
| PAHO–FluNet | Costa Rica | 2018/01-2023/12 | IV, RSV, PIV, MPV, sCoV, RV, AdV | Week | Case |
| PAHO–FluNet | Guatemala | 2018/01-2023/12 | IV, RSV, PIV, MPV, sCoV, RV, | Week | Case |
| PAHO–FluNet | Mexico | 2018/01-2023/12 | IV, RSV, PIV, MPV, sCoV, RV, AdV | Week | Case |
| Weekly national flu reports41 | England | 2019/07-2023/04 | IV, RSV, PIV, MPV, RV, AdV | Week | Proportion |
| Respiratory Scotland42 | Scotland | 2016/10-2024/04 | IV, RSV, PIV, MPV, sCoV, RV,AdV | Week | Case |
| Arbeitsgemeinschaft Influenza43 | Germany | 2016/12-2024/12 | IV, RSV, PIV, MPV, sCoV, RV, AdV | Week | Case |

**Supplementary Table 3. Description of included data from RSV GEN44.**

| Country | Site | Study period | Reported viruses | Reporting frequency | Indicator(s) |
|---|---|---|---|---|---|
| Argentina | Buenos Aires | 2018/01-2023/12 | IV, RSV, PIV, MPV, sCoV, RV, HadV | Month | Proportion & Case |
| Australia | Melbourne | 2017/01-2023/12 | IV, RSV, PIV, MPV , RV, HadV | Month | Proportion & Case |
| Canada | Halifax | 2017/01-2023/12 | IV, RSV, PIV, MPV, sCoV, RV, AdV | Month | Proportion & Case |
| China | Shenzhen | 2017/01-2023/12 | IV, RSV, PIV, MPV, sCoV, RV, AdV | Month | Proportion & Case |
| France | Marseille | 2017/01-2023/12 | IV, RSV, PIV, MPV, sCoV, RV, AdV | Month | Proportion & Case |
| Georgia | Tbilisi | 2017/01-2023/12 | IV, RSV, PIV, MPV, sCoV, RV, AdV | Month | Proportion & Case |
| Israel | Israel | 2017/01-2023/12 | IV, RSV, PIV, MPV, AdV | Month | Proportion & Case |
| Kenya | Kilifi | 2017/01-2023/12 | IV, RSV, PIV, MPV, sCoV, RV, AdV | Month | Proportion & Case |
| Netherlands | Surveillance | 2017/01-2023/12 | IV, RSV, MPV, SCoV, RV, AdV | Week | Case |
| Portugal | Lisbon | 2017/01-2023/12 | IV, RSV, PIV, MPV, RV, AdV | Month | Proportion & Case |
| Russia | Multi cities | 2017/01-2023/12 | IV, RSV, PIV, MPV, sCoV, RV, AdV | Week | Proportion & Case |
| Singapore | Singapore | 2018/01-2023/12 | IV, RSV, PIV, MPV, sCoV, AdV | Month | Proportion & Case |
| Spain | Barcelona | 2017/01-2023/12 | IV, RSV, PIV, MPV | Month | Proportion & Case |
| Thailand | Bangkok | 2017/01-2023/12 | IV, RSV, MPV | Month | Proportion & Case |
| United States | Alaska | 2019/11-2023/12 | IV, RSV, MPV, RV | Month | Proportion & Case |

**Supplementary Table 4. Quality assessment results of included studies from the systematic literature search.**

| Author | Q1 | Q2 | Q3 | Q4 | Q5 | Q6 | Q7 | Q8 | Total point |
|---|---|---|---|---|---|---|---|---|---|
| Sheila F Lumley | yes | no | no | no | yes | yes | yes | not clear | 4 |
| Yohei Kume | no | no | no | no | no | yes | yes | not clear | 2 |
| Hye Jin Shi | yes | yes | no | yes | yes | yes | yes | no | 6 |
| Xiucui Han | no | no | no | yes | no | not clear | no | not clear | 1 |
| A. A. Sominina | yes | yes | yes | yes | yes | yes | yes | not clear | 7 |
| Pengcheng Liu | no | no | no | yes | no | no | no | not clear | 1 |
| Asmae Lamrani Hanchi | no | no | no | no | no | yes | no | not clear | 1 |
| Chungmin Park | yes | yes | yes | yes | no | yes | yes | not clear | 6 |
| Lu Wan | yes | no | no | yes | no | yes | no | no | 3 |
| Ron Dagan | no | no | no | yes | no | no | yes | no | 2 |
| Norosoa Harline Razanajatovo | yes | yes | yes | no | yes | yes | yes | no | 6 |
| Dana Danino | yes | no | no | yes | no | yes | no | not clear | 3 |
| Heping Wang | no | no | no | yes | no | no | yes | no | 2 |
| Ilana S. Fratty | no | yes | yes | no | no | yes | yes | not clear | 4 |
| Zaid Haddadin | no | yes | no | yes | no | yes | yes | not clear | 4 |
| Yonatan Oster | no | yes | no | no | no | yes | yes | no | 3 |
| Ying Li | no | no | no | no | no | yes | yes | not clear | 2 |
| Chuang-Xing Lin | no | no | no | no | no | yes | yes | not clear | 2 |
| Yuan Feng | no | no | no | no | no | yes | yes | not clear | 2 |
| Nicole Maison | no | no | no | no | no | yes | yes | not clear | 2 |
| Xue Fu | no | yes | no | no | no | yes | yes | not clear | 3 |
| Yuya Fukuda | yes | no | no | no | no | no | yes | not clear | 2 |
| Rundong Cao | no | no | no | no | no | yes | yes | not clear | 2 |
| Víctor Guadalupe-Fernández | yes | yes | yes | no | no | no | yes | not clear | 4 |
| Yingchan Hao | no | no | no | no | no | yes | yes | not clear | 2 |
| Mingyu Tang | no | no | no | no | no | no | yes | not clear | 1 |
| Jianbo Xia | no | no | no | no | no | yes | yes | not clear | 2 |
| Michaela Davids | yes | yes | no | no | no | yes | yes | not clear | 4 |
| J. Dina | yes | no | yes | yes | no | yes | yes | yes | 6 |
| Füsun Kırca | no | yes | no | yes | no | yes | yes | not clear | 4 |
| Wai-Sing Chan | yes | yes | no | yes | no | yes | yes | not clear | 5 |

**Supplementary Table 5. Quality assessment results of included surveillance reports.**

| Database name | Country | Location | Q1 | Q2 | Q3 | Q4 | Q5 | Q6 | Q7 | Q8 | Total point |
|---|---|---|---|---|---|---|---|---|---|---|---|
| The National Respiratory and Enteric Virus Surveillance System | United States | Nationwide | yes | Yes | yes | yes | yes | yes | yes | not clear | 7 |
| Seattle Flu Alliance | United States | Seattle | yes | yes | yes | yes | yes | yes | yes | not clear | 7 |
| Houston methodist-Respiratory Pathogen Epidemiology Snapshot | United States | Houston | yes | yes | yes | yes | yes | yes | no | not clear | 6 |
| Korea Disease Control and Prevention Agency | Korea, Rep. | Nationwide | yes | yes | yes | yes | yes | yes | yes | not clear | 7 |
| Finnish National Infectious Diseases Register | Finland | Nationwide | yes | yes | no | yes | yes | yes | yes | not clear | 6 |
| Finnish National Infectious Diseases Register | Finland | Etelä-Pohjanmaan sairaanhoitopiiri | yes | yes | no | no | yes | yes | no | not clear | 4 |
| Finnish National Infectious Diseases Register | Finland | Helsingin ja Uudenmaan sairaanhoitopiiri | yes | yes | no | yes | yes | yes | no | not clear | 5 |
| Finnish National Infectious Diseases Register | Finland | Lapin sairaanhoitopiiri | yes | yes | no | no | yes | yes | no | not clear | 4 |
| Finnish National Infectious Diseases Register | Finland | Pirkanmaan sairaanhoitopiiri | yes | yes | no | no | yes | yes | no | not clear | 4 |
| Finnish National Infectious Diseases Register | Finland | Pohjois-Pohjanmaan sairaanhoitopiiri | yes | yes | no | yes | yes | yes | yes | not clear | 6 |
| Finnish National Infectious Diseases Register | Finland | Vaasan sairaanhoitopiiri | yes | yes | no | yes | yes | yes | yes | not clear | 6 |

| | | | | | | | | | | |
|---|---|---|---|---|---|---|---|---|---|---|
| Finnish National Infectious Diseases Register | Finland | Varsinais-Suomen sairaanhoitopiiri | yes | yes | no | yes | yes | yes | yes | not clear | 6 |
| Taiwan National Infectious Disease Statistics System | China | Taiwan | yes | yes | yes | no | yes | yes | no | not clear | 5 |
| Czech Republic National institutes of health | Czech Republic | Nationwide | yes | yes | yes | no | yes | yes | yes | not clear | 6 |
| FluWatch Surveillance | Canada | Atlantic | yes | yes | yes | yes | yes | yes | yes | not clear | 7 |
| FluWatch Surveillance | Canada | New Brunswick, Atlantic | yes | yes | yes | no | no | yes | yes | not clear | 5 |
| FluWatch Surveillance | Canada | Newfoundland, Atlantic | yes | yes | yes | yes | yes | yes | yes | not clear | 7 |
| FluWatch Surveillance | Canada | Nova Scotia, Atlantic | yes | yes | yes | no | yes | yes | yes | not clear | 6 |
| FluWatch Surveillance | Canada | Alberta, Prairies | yes | yes | yes | yes | yes | yes | yes | not clear | 7 |
| FluWatch Surveillance | Canada | Manitoba, Prairies | yes | yes | yes | no | yes | yes | yes | not clear | 6 |
| FluWatch Surveillance | Canada | Prairies | yes | yes | yes | no | yes | yes | yes | not clear | 6 |
| FluWatch Surveillance | Canada | Saskatchewan, Prairies | yes | yes | yes | no | yes | yes | yes | not clear | 6 |
| FluWatch Surveillance | Canada | Hamilton, Province of Ontario | yes | yes | yes | no | yes | yes | yes | not clear | 6 |
| FluWatch Surveillance | Canada | Kingston, Province of Ontario | yes | yes | yes | no | yes | yes | yes | not clear | 6 |
| FluWatch Surveillance | Canada | London, Province of Ontario | yes | yes | yes | no | yes | yes | yes | not clear | 6 |
| FluWatch Surveillance | Canada | Orillia, Province of Ontario | yes | yes | yes | no | yes | yes | yes | not clear | 6 |

| | | | | | | | | | | | |
|---|---|---|---|---|---|---|---|---|---|---|---|
| FluWatch Surveillance | Canada | Ottawa, Province of Ontario | yes | yes | yes | yes | yes | yes | yes | not clear | 7 |
| FluWatch Surveillance | Canada | Toronto, Province of Ontario | yes | yes | yes | yes | yes | yes | yes | not clear | 7 |
| FluWatch Surveillance | Canada | Province of Ontario | yes | yes | yes | yes | yes | yes | yes | not clear | 7 |
| FluWatch Surveillance | Canada | St. Joseph's - Hamilton, Province of Ontario | yes | yes | yes | yes | yes | yes | yes | not clear | 7 |
| FluWatch Surveillance | Canada | Montréal-Laval, Province of Québec | yes | yes | yes | yes | yes | yes | yes | not clear | 7 |
| FluWatch Surveillance | Canada | Province of Québec | yes | yes | yes | yes | yes | yes | yes | not clear | 7 |
| FluWatch Surveillance | Canada | Québec-Chaudière-Appalaches, Province of Québec | yes | yes | yes | yes | yes | yes | yes | not clear | 7 |
| FluWatch Surveillance | Canada | British Columbia | yes | yes | yes | no | yes | yes | yes | not clear | 6 |
| FluWatch Surveillance | Canada | Nationwide | yes | yes | yes | yes | yes | yes | yes | not clear | 7 |
| PAHO–FluNet | Bolivia | Nationwide | yes | yes | yes | no | yes | yes | yes | not clear | 6 |
| PAHO–FluNet | Paraguay | Nationwide | yes | yes | yes | no | yes | yes | yes | not clear | 6 |
| PAHO–FluNet | Colombia | Nationwide | yes | yes | yes | yes | yes | yes | yes | not clear | 7 |
| PAHO–FluNet | Argentina | Nationwide | yes | yes | yes | yes | yes | yes | yes | not clear | 7 |
| PAHO–FluNet | Chile | Nationwide | yes | yes | yes | no | yes | yes | yes | not clear | 6 |
| PAHO–FluNet | Costa Rica | Nationwide | yes | yes | yes | yes | yes | yes | yes | not clear | 7 |
| PAHO–FluNet | Guatemala | Nationwide | yes | yes | yes | no | yes | yes | yes | not clear | 6 |
| PAHO–FluNet | Mexico | Nationwide | yes | yes | yes | no | yes | yes | yes | not clear | 6 |
| Weekly national flu reports | United Kingdom | England | yes | yes | yes | yes | yes | yes | yes | not clear | 7 |

| | | | | | | | | | | | |
|---|---|---|---|---|---|---|---|---|---|---|---|
| Public Health Scotland | United Kingdom | Scotland | yes | yes | yes | yes | yes | yes | yes | not clear | 7 |
| Arbeitsgemeinschaft Influenza | Germany | Nationwide | yes | yes | yes | yes | yes | yes | yes | not clear | 7 |

**Supplementary Table 6. Quality assessment results of data from RSV GEN.**

| Country | Location | Q1 | Q2 | Q3 | Q4 | Q5 | Q6 | Q7 | Q8 | Total point |
|---|---|---|---|---|---|---|---|---|---|---|
| Argentina | Buenos Aires | yes | no | no | yes | yes | yes | yes | not clear | 5 |
| Australia | Melbourne | no | yes | no | yes | yes | yes | yes | not clear | 5 |
| Canada | Halifax | no | yes | no | no | yes | yes | yes | not clear | 4 |
| China | Shenzhen | no | yes | no | yes | yes | no | yes | not clear | 4 |
| France | Marseille | yes | yes | no | yes | yes | yes | yes | not clear | 6 |
| Georgia | Tbilisi | yes | yes | no | yes | yes | yes | yes | not clear | 6 |
| Israel | Israel | yes | yes | no | yes | yes | yes | yes | not clear | 6 |
| Kenya | Kilifi | no | no | no | no | yes | yes | no | not clear | 2 |
| Netherlands | Surveillance | yes | yes | yes | yes | yes | yes | yes | not clear | 7 |
| Portugal | Lisbon | no | no | no | yes | yes | yes | yes | not clear | 4 |
| Russia | Multi cities | yes | no | yes | no | yes | yes | yes | not clear | 5 |
| Singapore | Singapore | no | yes | no | yes | yes | yes | yes | not clear | 5 |
| Spain | Barcelona | no | no | no | yes | yes | yes | no | not clear | 3 |
| Thailand | Bangkok | yes | yes | no | yes | yes | yes | no | not clear | 5 |
| United States | Alaska | yes | no | no | no | no | yes | yes | not clear | 3 |

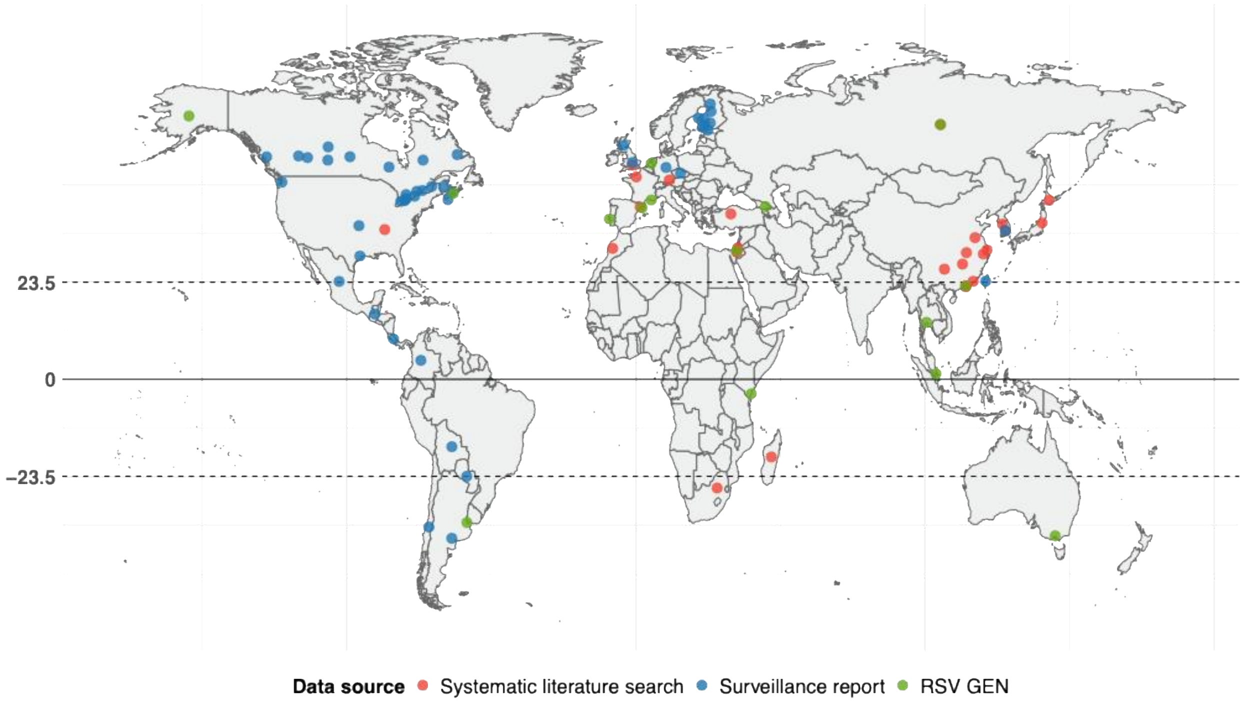


**Supplementary Figure 1.Distribution of common respiratory virus activity in this study.**

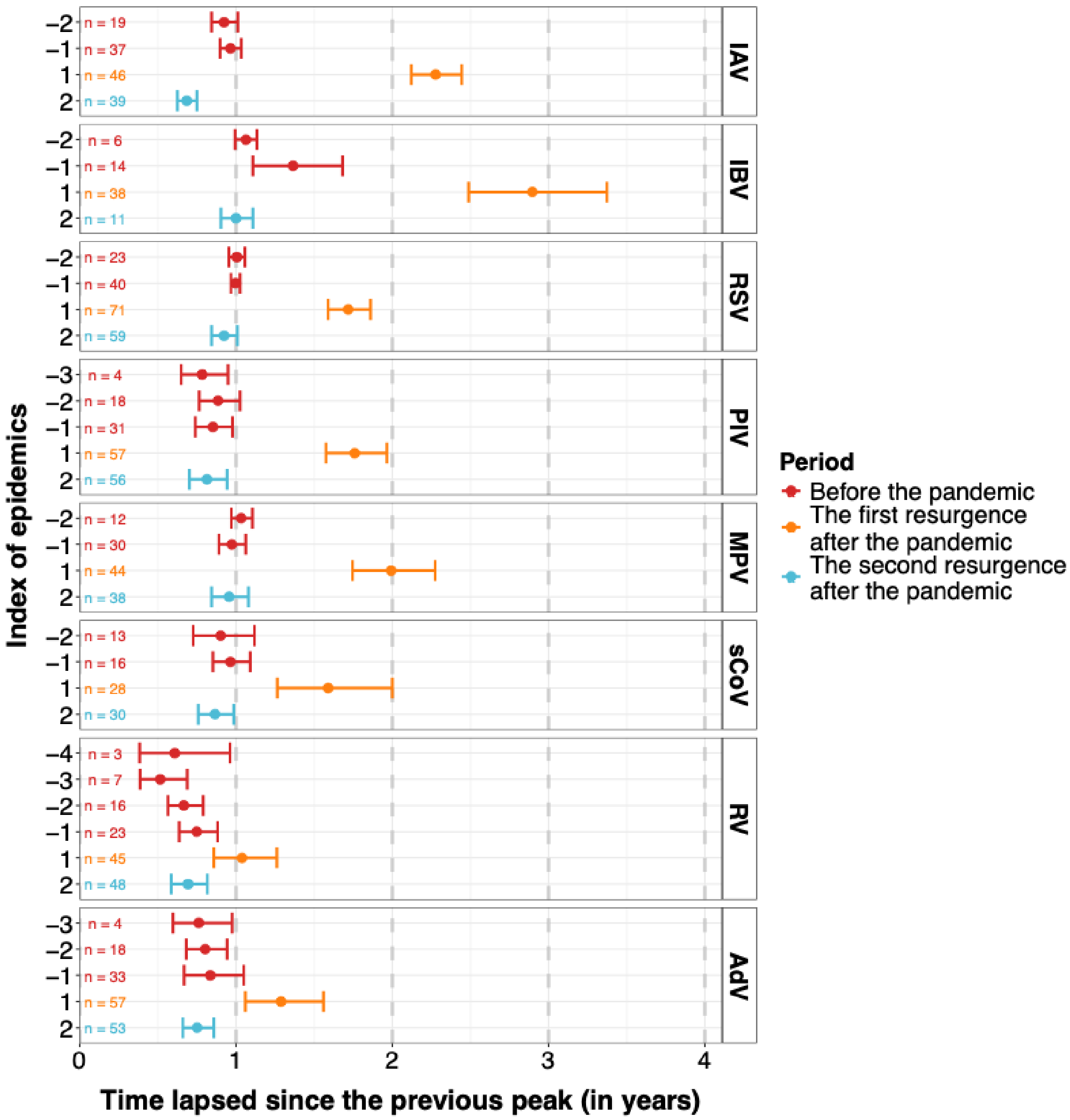


**Supplementary Figure 2. The virus-specific time interval (with 95% CI) between the peaks in two subsequent years in temperate regions.**

The x-axis represents the time lapsed since the previous onset or peak, measured in years. The left y-axis lists the index of epidemics relative to the onset of the COVID-19 pandemic, with negative numbers indicating epidemics occurring before the pandemic, and positive numbers indicating epidemics occurring after the onset of the pandemic. Data are presented as mean values and error bars indicate their 95% confidence intervals. IAV = Influenza A virus; IBV = Influenza B virus; RSV = Respiratory Syncytial Virus; PIV = Parainfluenza Virus; MPV = Metapneumovirus; sCoV = Seasonal Coronavirus; RV = Rhinovirus; AdV = Adenovirus (AdV).

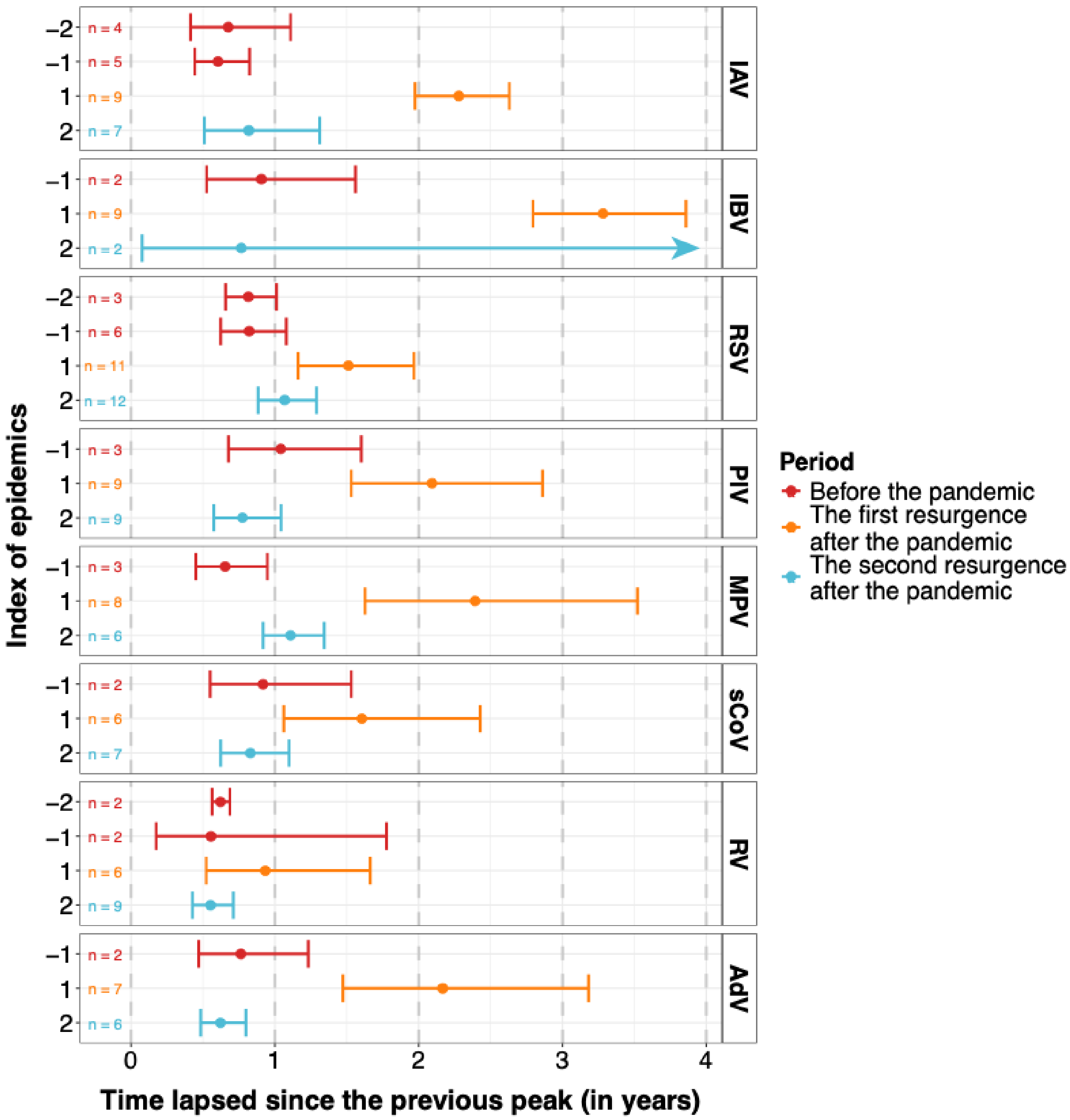


**Supplementary Figure 3.The virus-specific time interval (with 95% CI) between the peaks in two subsequent years in tropics regions.**

The x-axis represents the time lapsed since the previous onset or peak, measured in years. The left y-axis lists the index of epidemics relative to the onset of the COVID-19 pandemic, with negative numbers indicating epidemics occurring before the pandemic, and positive numbers indicating epidemics occurring after the onset of the pandemic. Data are presented as mean values and error bars indicate their 95% confidence intervals. IAV = Influenza A virus; IBV = Influenza B virus; RSV = Respiratory Syncytial Virus; PIV = Parainfluenza Virus; MPV = Metapneumovirus; sCoV = Seasonal Coronavirus; RV = Rhinovirus; AdV = Adenovirus (AdV).

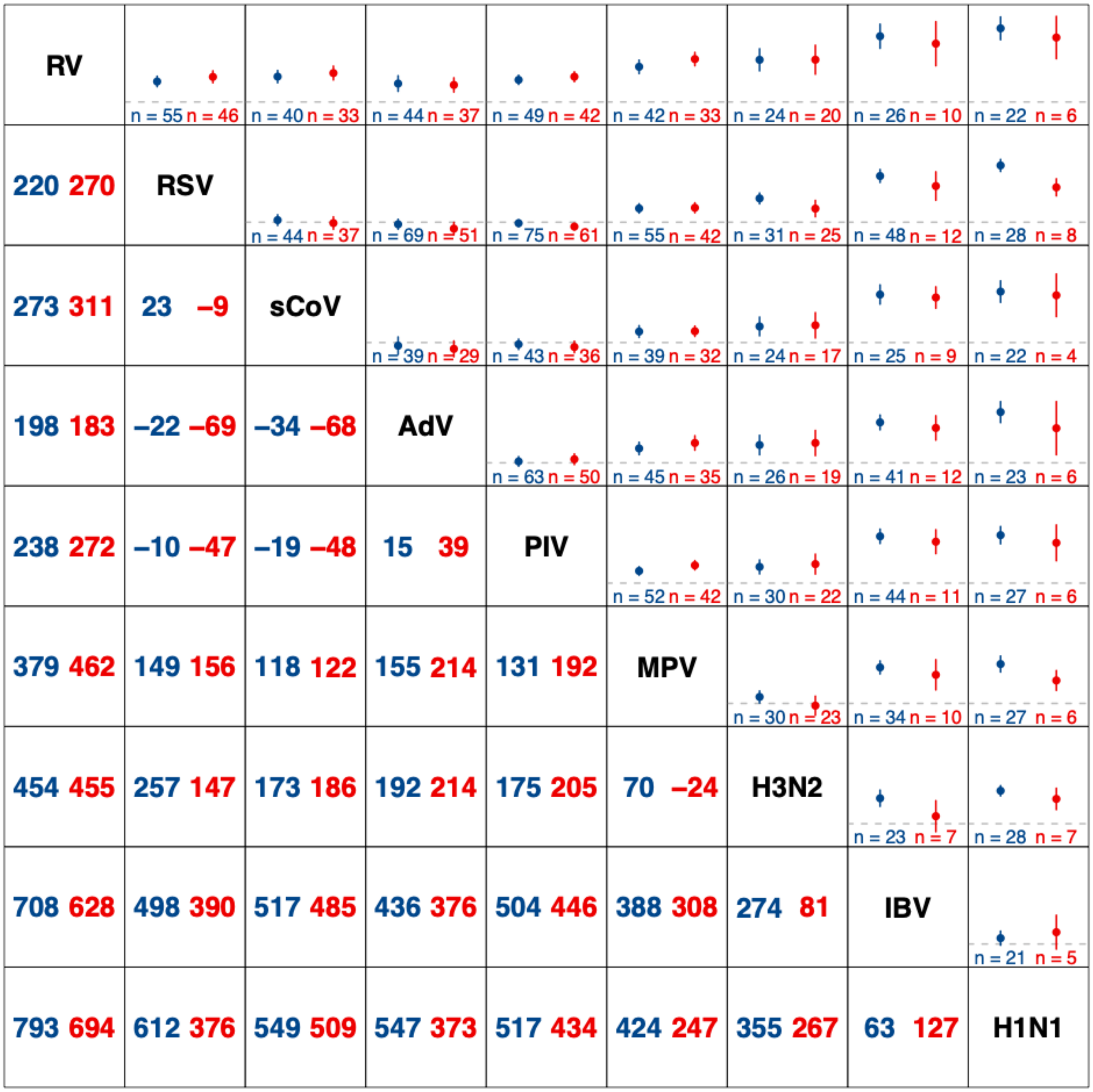


**Supplementary Figure 4. The time difference in days of onset between viruses during their first resurgence and second resurgence following the COVID-19 pandemic onset, matched by sites reporting influenza A virus subtypes.**

The matrix presents the pairwise comparison of timing between viruses in their first (shown in blue) and second resurgence (shown in red). Positive [negative] values indicated that the virus on the top / left resurged earlier [later] than the virus on the bottom / right. Dashed line indicated no differences in the timing. Data are presented as mean values and error bars indicate their 95% confidence intervals. IAV = Influenza A virus; IBV = Influenza B virus; RSV = Respiratory Syncytial Virus; PIV = Parainfluenza Virus; MPV = Metapneumovirus; sCoV = Seasonal Coronavirus; RV = Rhinovirus; AdV = Adenovirus (AdV).

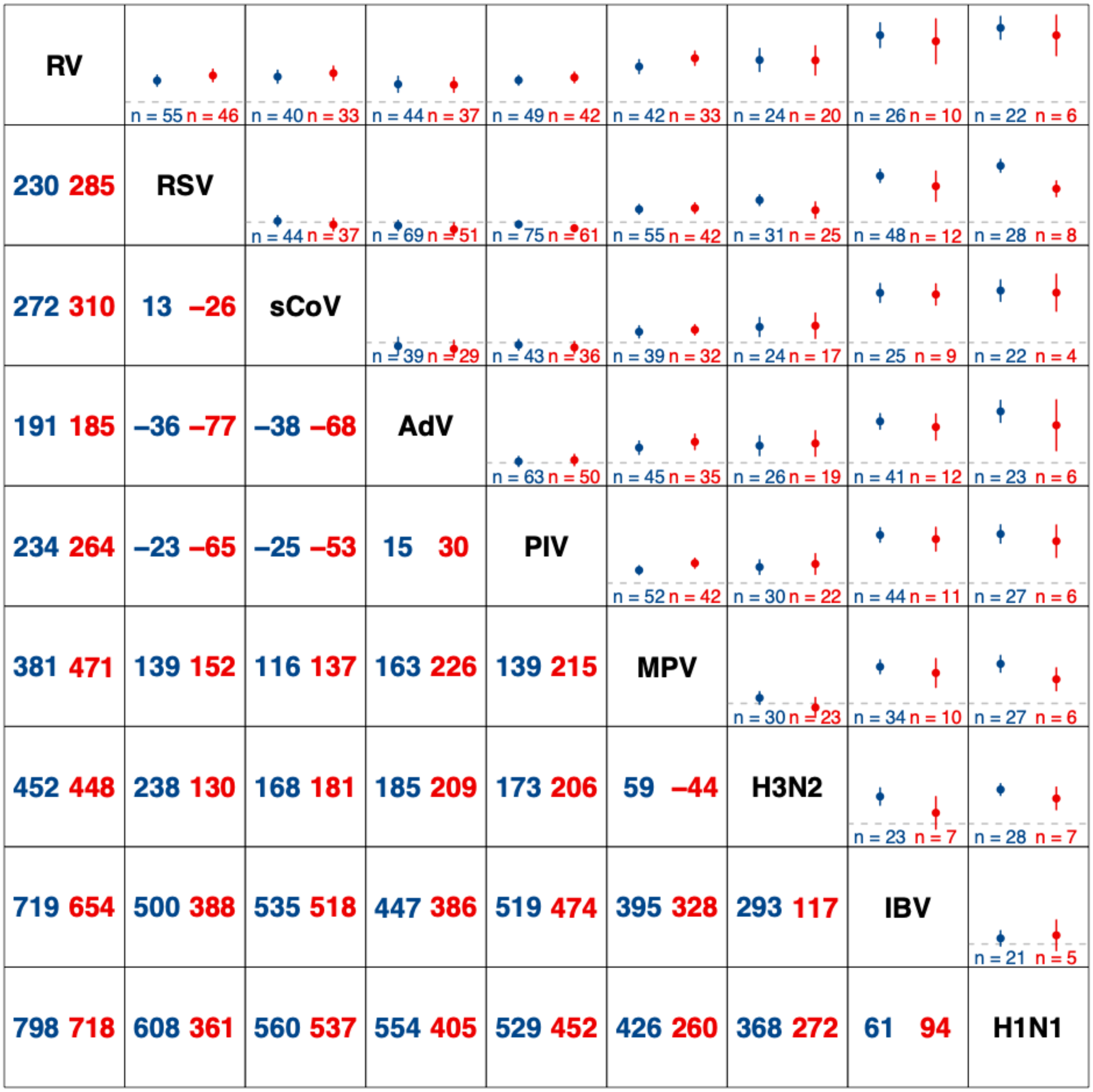


**Supplementary Figure 5. The time difference in days of peak between viruses during their first resurgence and second resurgence following the COVID-19 pandemic onset, matched by sites reporting influenza A virus subtypes.**

The matrix presents the pairwise comparison of timing between viruses in their first (shown in blue) and second resurgence (shown in red). Positive [negative] values indicated that the virus on the top / left resurged earlier [later] than the virus on the bottom / right. Dashed line indicated no differences in the timing. Data are presented as mean values and error bars indicate their 95% confidence intervals. IAV = Influenza A virus; IBV = Influenza B virus; RSV = Respiratory Syncytial Virus; PIV = Parainfluenza Virus; MPV = Metapneumovirus; sCoV = Seasonal Coronavirus; RV = Rhinovirus; AdV = Adenovirus (AdV).

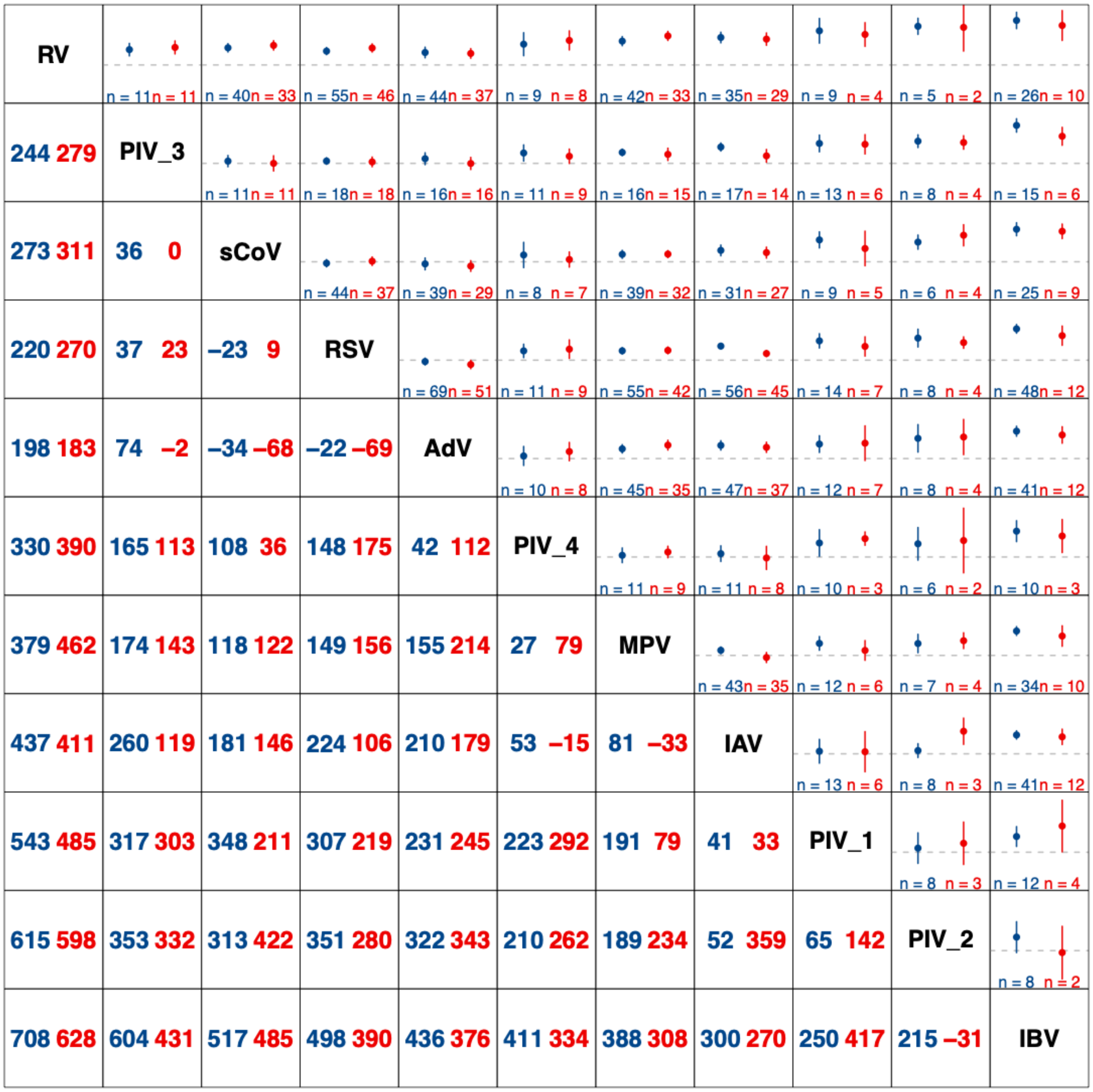


**Supplementary Figure 6. The time difference in days of onset between viruses during their first resurgence and second resurgence following the COVID-19 pandemic onset, matched by sites reporting PIV subtypes.**

The matrix presents the pairwise comparison of timing between viruses in their first (shown in blue) and second resurgence (shown in red). Positive [negative] values indicated that the virus on the top / left resurged earlier [later] than the virus on the bottom / right. Dashed line indicated no differences in the timing. Data are presented as mean values and error bars indicate their 95% confidence intervals. IAV = Influenza A virus; IBV = Influenza B virus; RSV = Respiratory Syncytial Virus; PIV = Parainfluenza Virus; MPV = Metapneumovirus; sCoV = Seasonal Coronavirus; RV = Rhinovirus; AdV = Adenovirus (AdV).

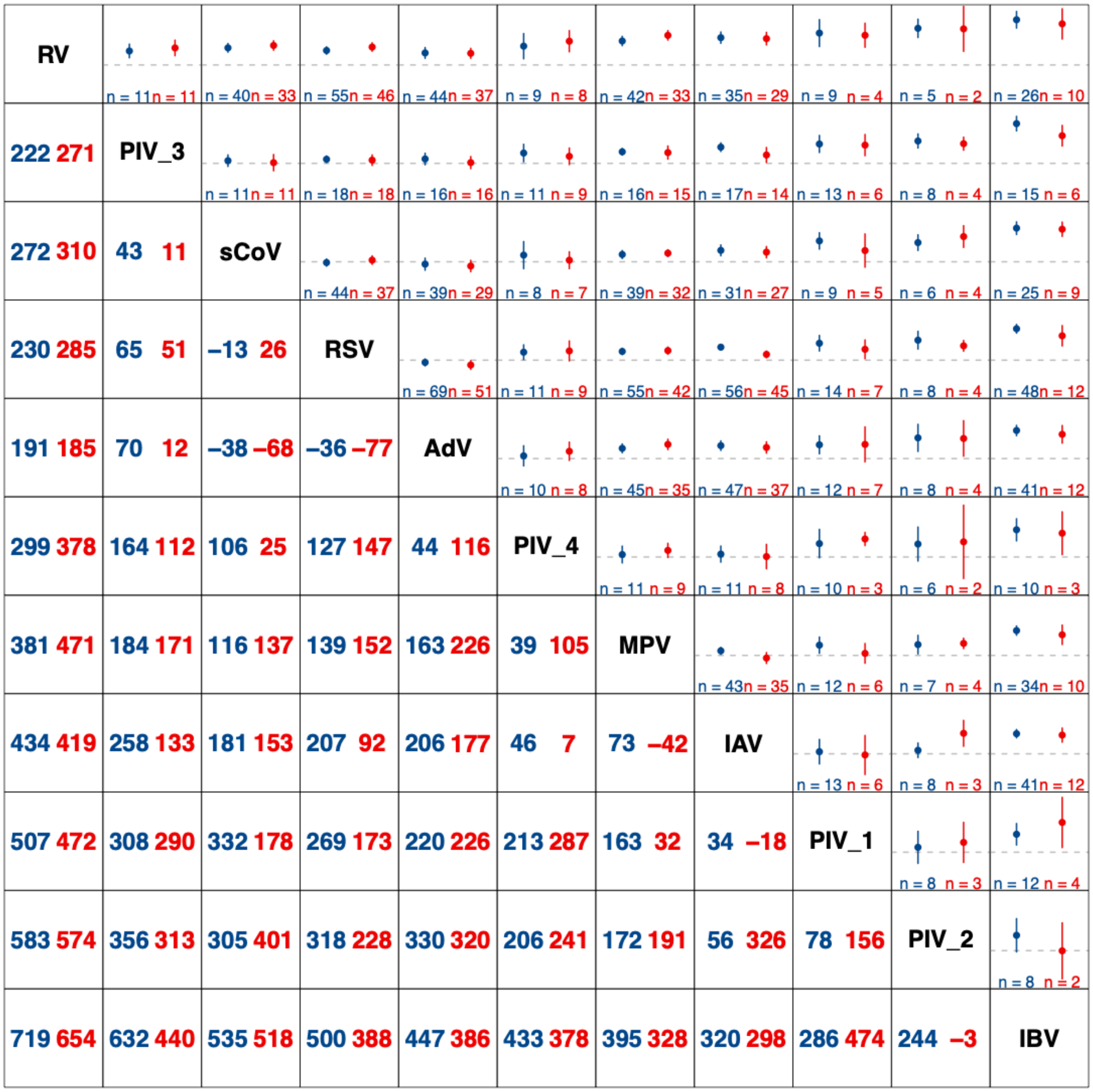


**Supplementary Figure 7. The time difference in days of peak between viruses during their first resurgence and second resurgence following the COVID-19 pandemic onset, matched by sites reporting PIV subtypes.**

The matrix presents the pairwise comparison of timing between viruses in their first (shown in blue) and second resurgence (shown in red). Positive [negative] values indicated that the virus on the top / left resurged earlier [later] than the virus on the bottom / right. Dashed line indicated no differences in the timing. Data are presented as mean values and error bars indicate their 95% confidence intervals. IAV = Influenza A virus; IBV = Influenza B virus; RSV = Respiratory Syncytial Virus; PIV = Parainfluenza Virus; MPV = Metapneumovirus; sCoV = Seasonal Coronavirus; RV = Rhinovirus; AdV = Adenovirus (AdV).

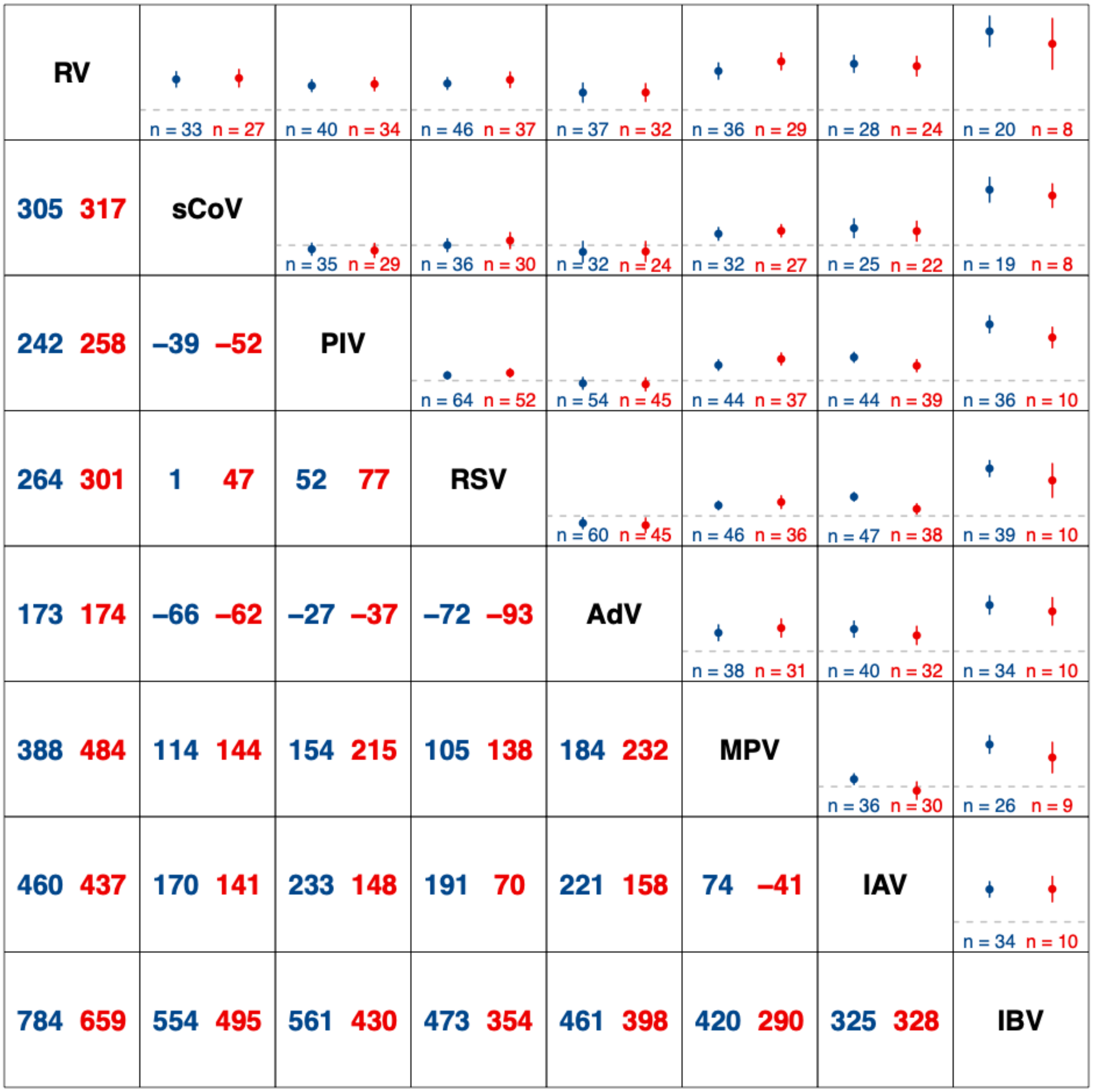


**Supplementary Figure 8. The time difference in days of peak between viruses during their first resurgence and second resurgence following the COVID-19 pandemic onset, matched by study sites in the temperate region.**

The matrix presents the pairwise comparison of timing between viruses in their first (shown in blue) and second resurgence (shown in red). Positive [negative] values indicated that the virus on the top / left resurged earlier [later] than the virus on the bottom / right. Dashed line indicated no differences in the timing. Data are presented as mean values and error bars indicate their 95% confidence intervals. IAV = Influenza A virus; IBV = Influenza B virus; RSV = Respiratory Syncytial Virus; PIV = Parainfluenza Virus; MPV = Metapneumovirus; sCoV = Seasonal Coronavirus; RV = Rhinovirus; AdV = Adenovirus (AdV).

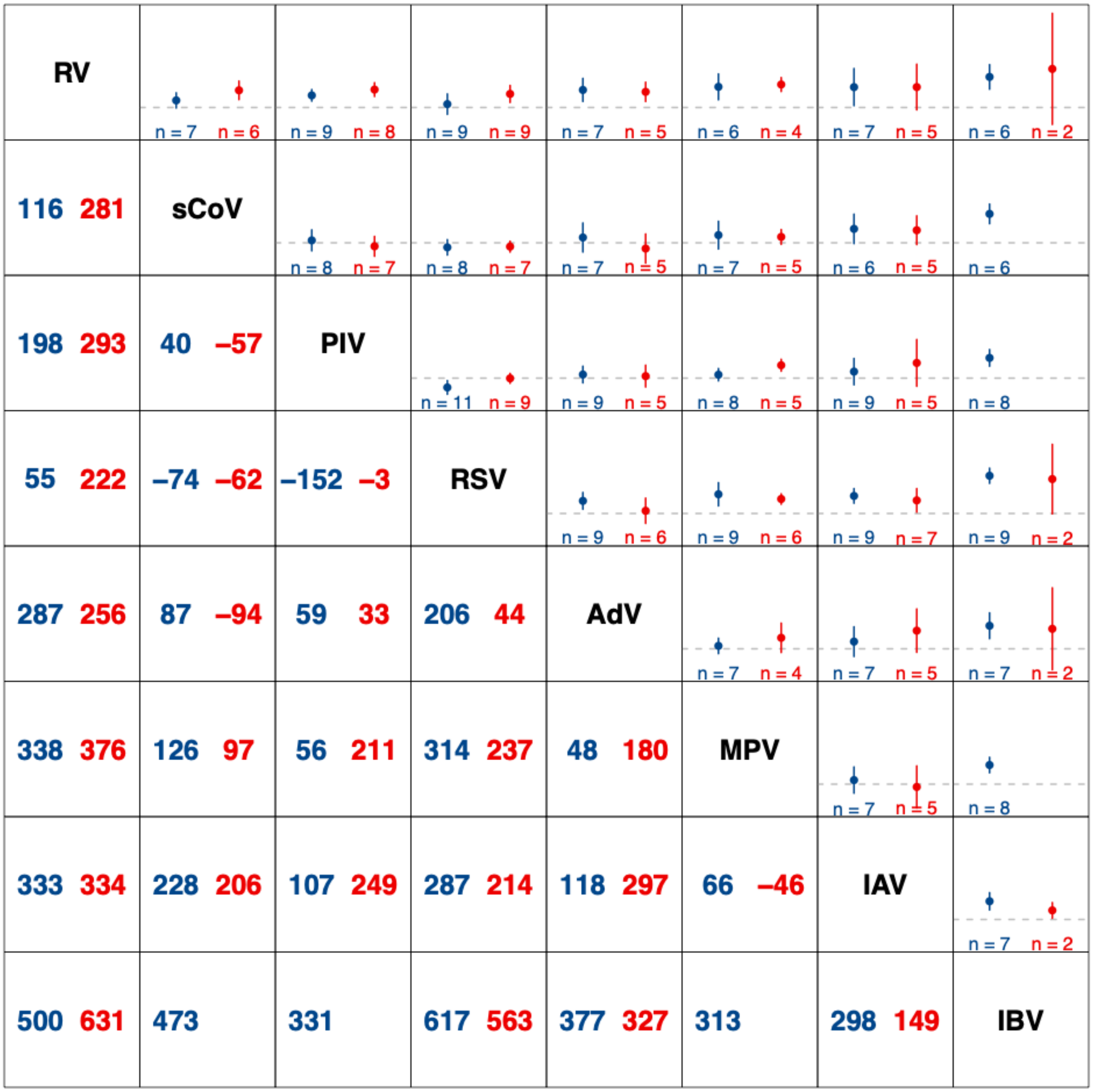


**Supplementary Figure 9. The time difference in days of peak between viruses during their first resurgence and second resurgence following the COVID-19 pandemic onset, matched by study sites in the tropical region.**

The matrix presents the pairwise comparison of timing between viruses in their first (shown in blue) and second resurgence (shown in red). Positive [negative] values indicated that the virus on the top / left resurged earlier [later] than the virus on the bottom / right. Dashed line indicated no differences in the timing. Data are presented as mean values and error bars indicate their 95% confidence intervals. IAV = Influenza A virus; IBV = Influenza B virus; RSV = Respiratory Syncytial Virus; PIV = Parainfluenza Virus; MPV = Metapneumovirus; sCoV = Seasonal Coronavirus; RV = Rhinovirus; AdV = Adenovirus (AdV).

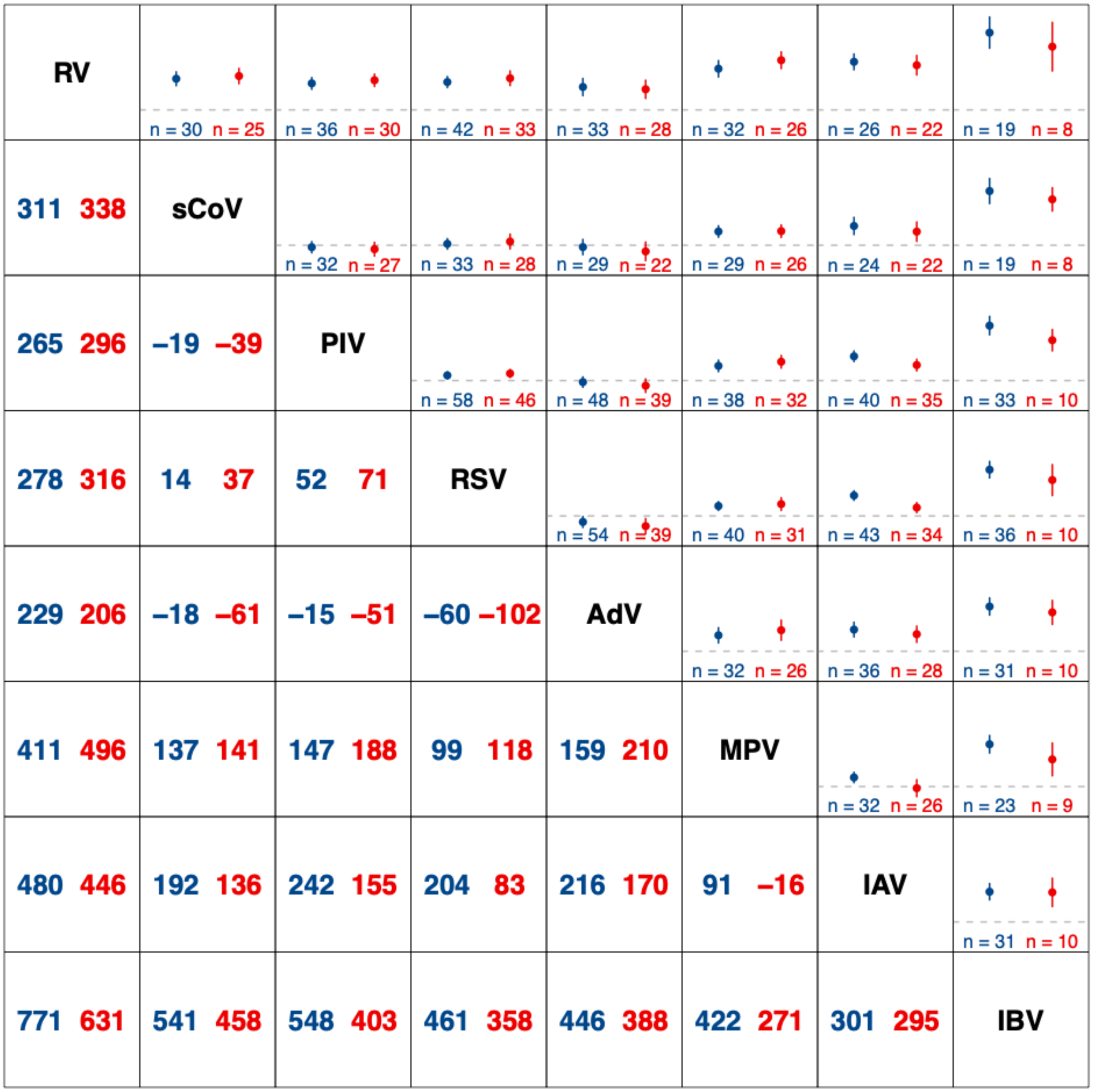


**Supplementary Figure 10. The time difference in days of onset between viruses during their first resurgence and second resurgence following the COVID-19 pandemic onset, matched by study sites in the northern temperate region.**

The matrix presents the pairwise comparison of timing between viruses in their first (shown in blue) and second resurgence (shown in red). Positive [negative] values indicated that the virus on the top / left resurged earlier [later] than the virus on the bottom / right. Dashed line indicated no differences in the timing. Data are presented as mean values and error bars indicate their 95% confidence intervals. IAV = Influenza A virus; IBV = Influenza B virus; RSV = Respiratory Syncytial Virus; PIV = Parainfluenza Virus; MPV = Metapneumovirus; sCoV = Seasonal Coronavirus; RV = Rhinovirus; AdV = Adenovirus (AdV).

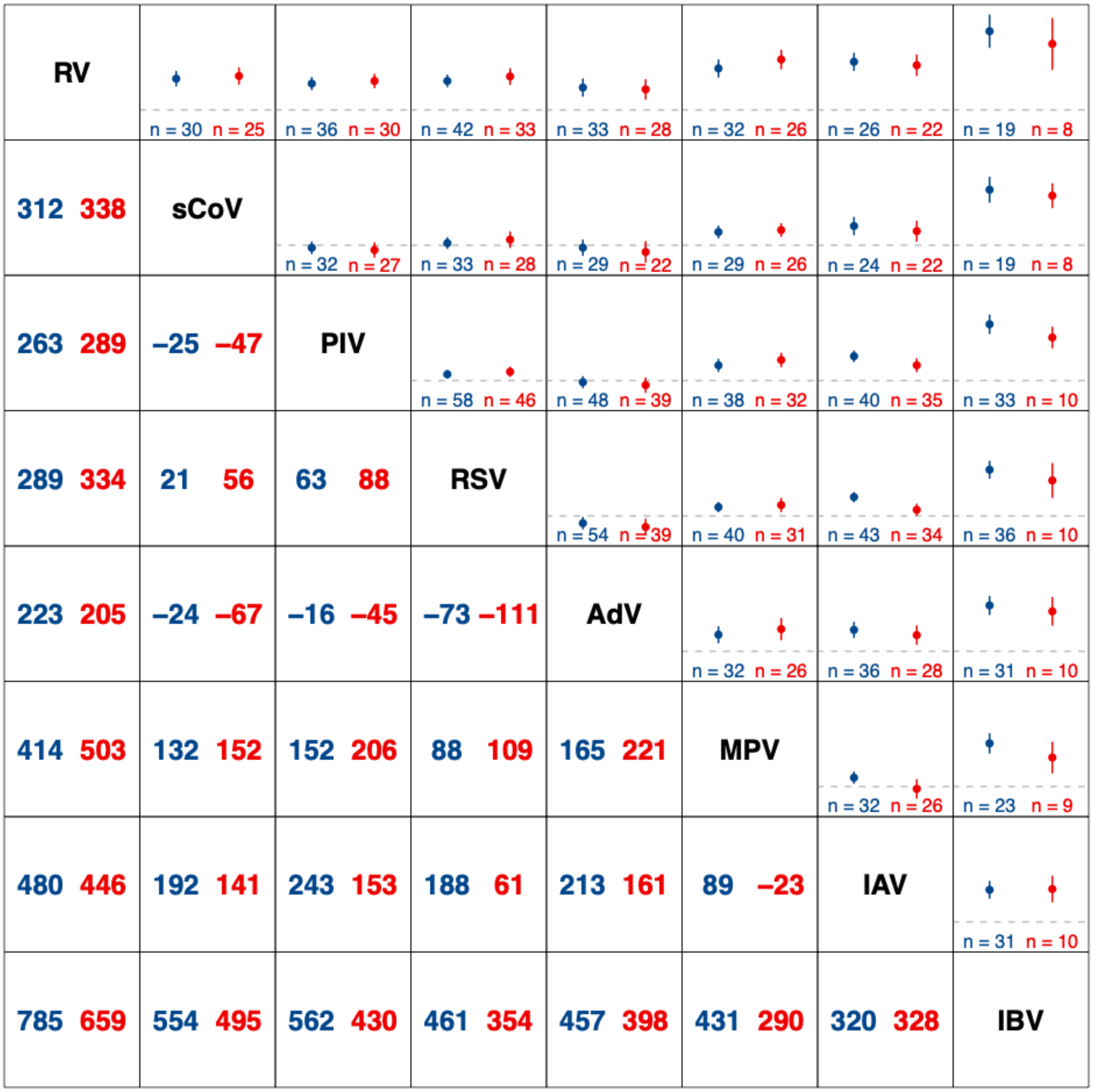


**Supplementary Figure 11. The time difference in days of peak between viruses during their first resurgence and second resurgence following the COVID-19 pandemic onset, matched by study sites in the northern temperate region.**

The matrix presents the pairwise comparison of timing between viruses in their first (shown in blue) and second resurgence (shown in red). Positive [negative] values indicated that the virus on the top / left resurged earlier [later] than the virus on the bottom / right. Dashed line indicated no differences in the timing. Data are presented as mean values and error bars indicate their 95% confidence intervals. IAV = Influenza A virus; IBV = Influenza B virus; RSV = Respiratory Syncytial Virus; PIV = Parainfluenza Virus; MPV = Metapneumovirus; sCoV = Seasonal Coronavirus; RV = Rhinovirus; AdV = Adenovirus (AdV).

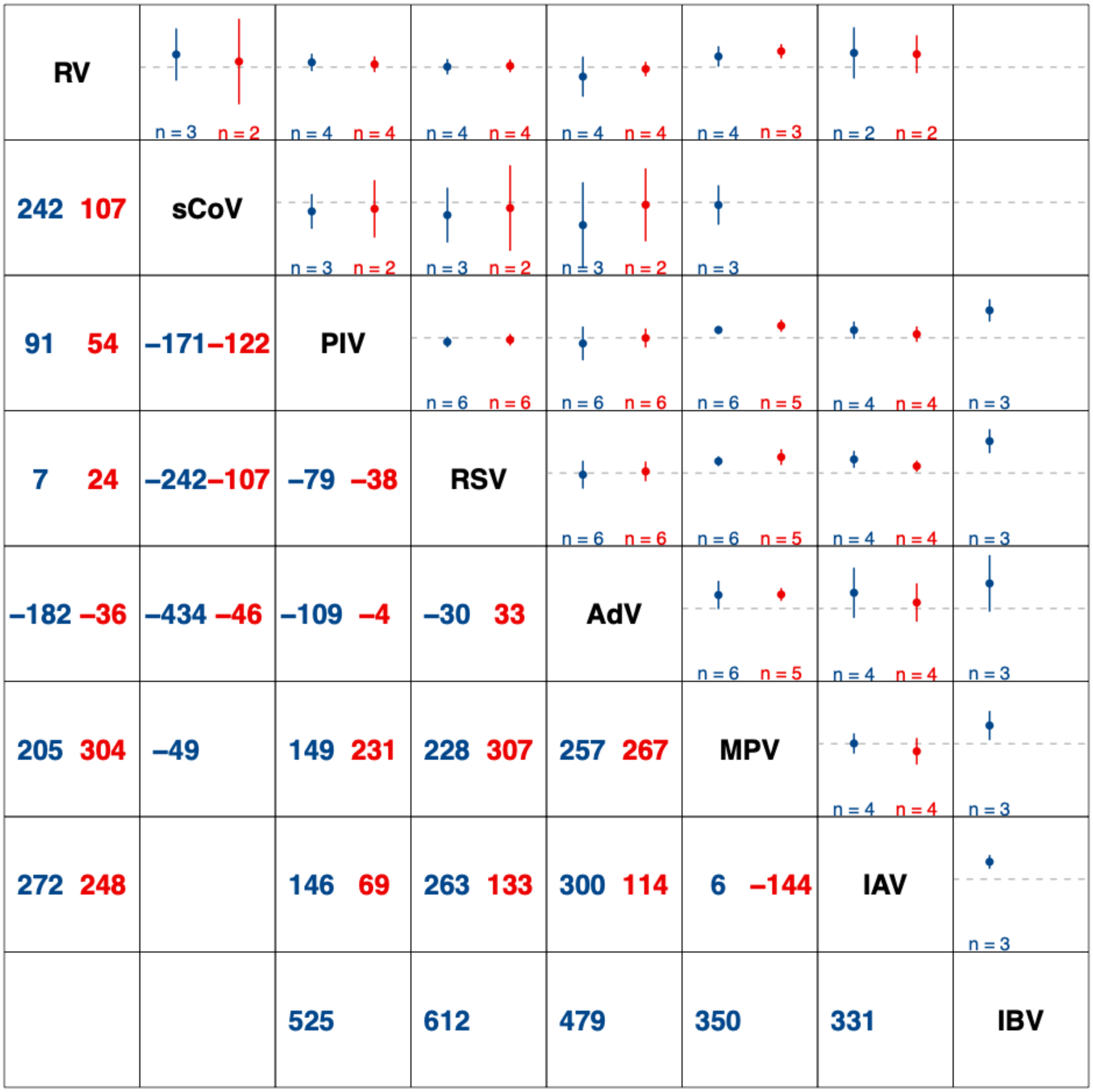


**Supplementary Figure 12. The time difference in days of onset between viruses during their first resurgence and second resurgence following the COVID-19 pandemic onset, matched by study sites in the southern temperate region.**

The matrix presents the pairwise comparison of timing between viruses in their first (shown in blue) and second resurgence (shown in red). Positive [negative] values indicated that the virus on the top / left resurged earlier [later] than the virus on the bottom / right. Dashed line indicated no differences in the timing. Data are presented as mean values and error bars indicate their 95% confidence intervals. IAV = Influenza A virus; IBV = Influenza B virus; RSV = Respiratory Syncytial Virus; PIV = Parainfluenza Virus; MPV = Metapneumovirus; sCoV = Seasonal Coronavirus; RV = Rhinovirus; AdV = Adenovirus (AdV).

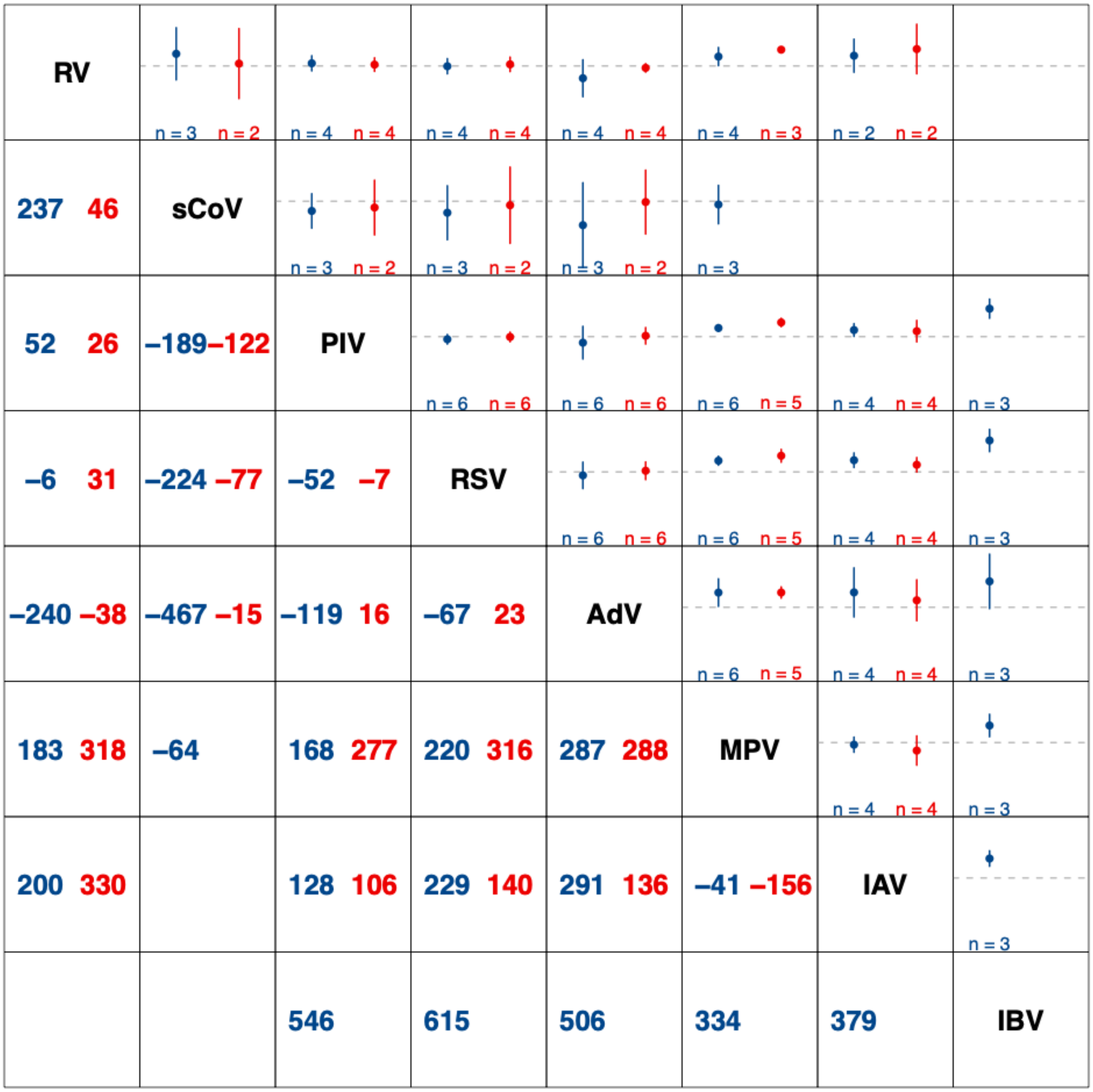


**Supplementary Figure 13. The time difference in days of peak between viruses during their first resurgence and second resurgence following the COVID-19 pandemic onset, matched by study sites in the southern temperate region.**

The matrix presents the pairwise comparison of timing between viruses in their first (shown in blue) and second resurgence (shown in red). Positive [negative] values indicated that the virus on the top / left resurged earlier [later] than the virus on the bottom / right. Dashed line indicated no differences in the timing. Data are presented as mean values and error bars indicate their 95% confidence intervals. IAV = Influenza A virus; IBV = Influenza B virus; RSV = Respiratory Syncytial Virus; PIV = Parainfluenza Virus; MPV = Metapneumovirus; sCoV = Seasonal Coronavirus; RV = Rhinovirus; AdV = Adenovirus (AdV).

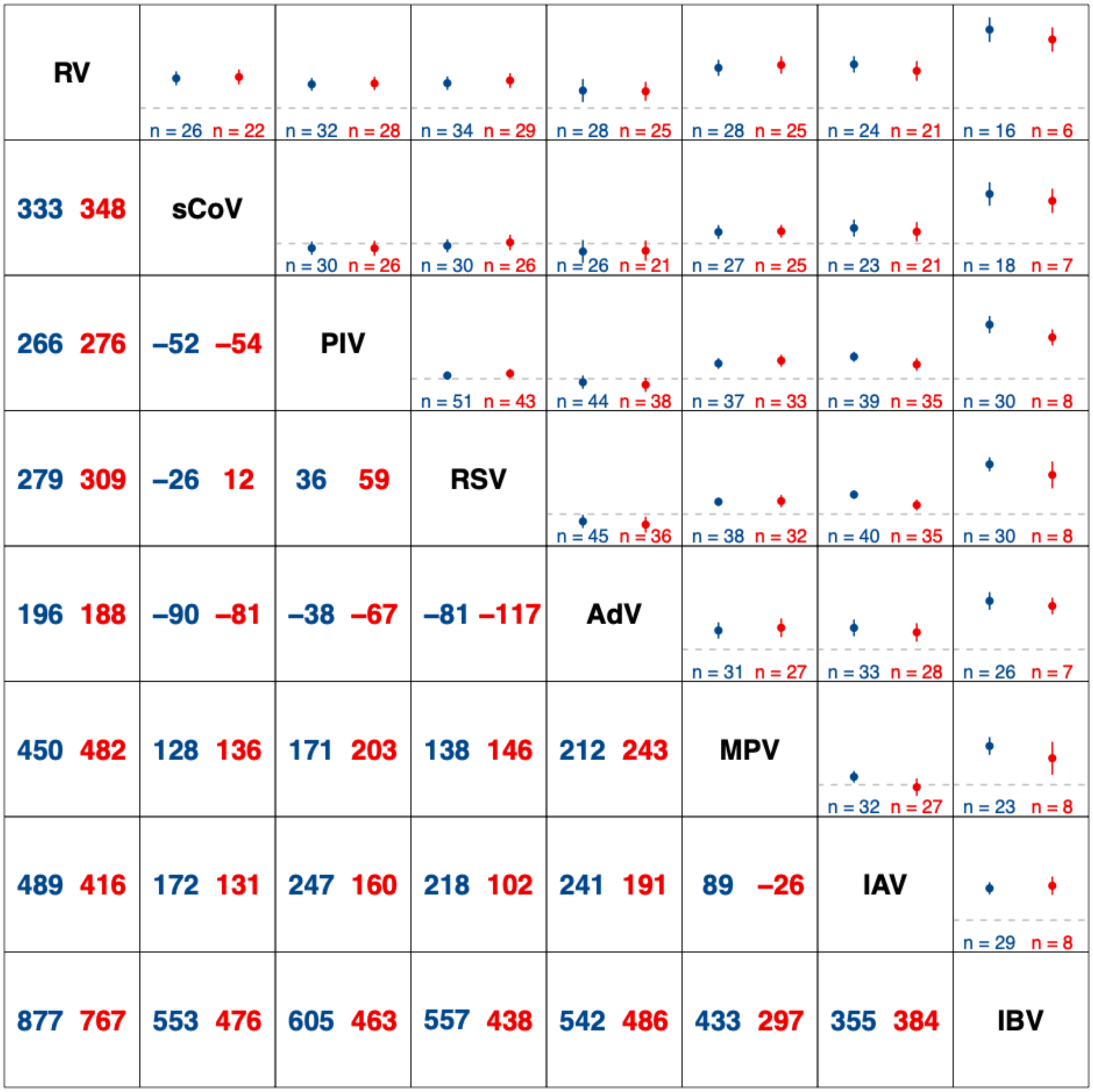


**Supplementary Figure 14. The time difference in days of onset between viruses during their first resurgence and second resurgence following the COVID-19 pandemic onset, matched by study sites in high-income countries.**

The matrix presents the pairwise comparison of timing between viruses in their first (shown in blue) and second resurgence (shown in red). Positive [negative] values indicated that the virus on the top / left resurged earlier [later] than the virus on the bottom / right. Dashed line indicated no differences in the timing. Data are presented as mean values and error bars indicate their 95% confidence intervals. IAV = Influenza A virus; IBV = Influenza B virus; RSV = Respiratory Syncytial Virus; PIV = Parainfluenza Virus; MPV = Metapneumovirus; sCoV = Seasonal Coronavirus; RV = Rhinovirus; AdV = Adenovirus (AdV).

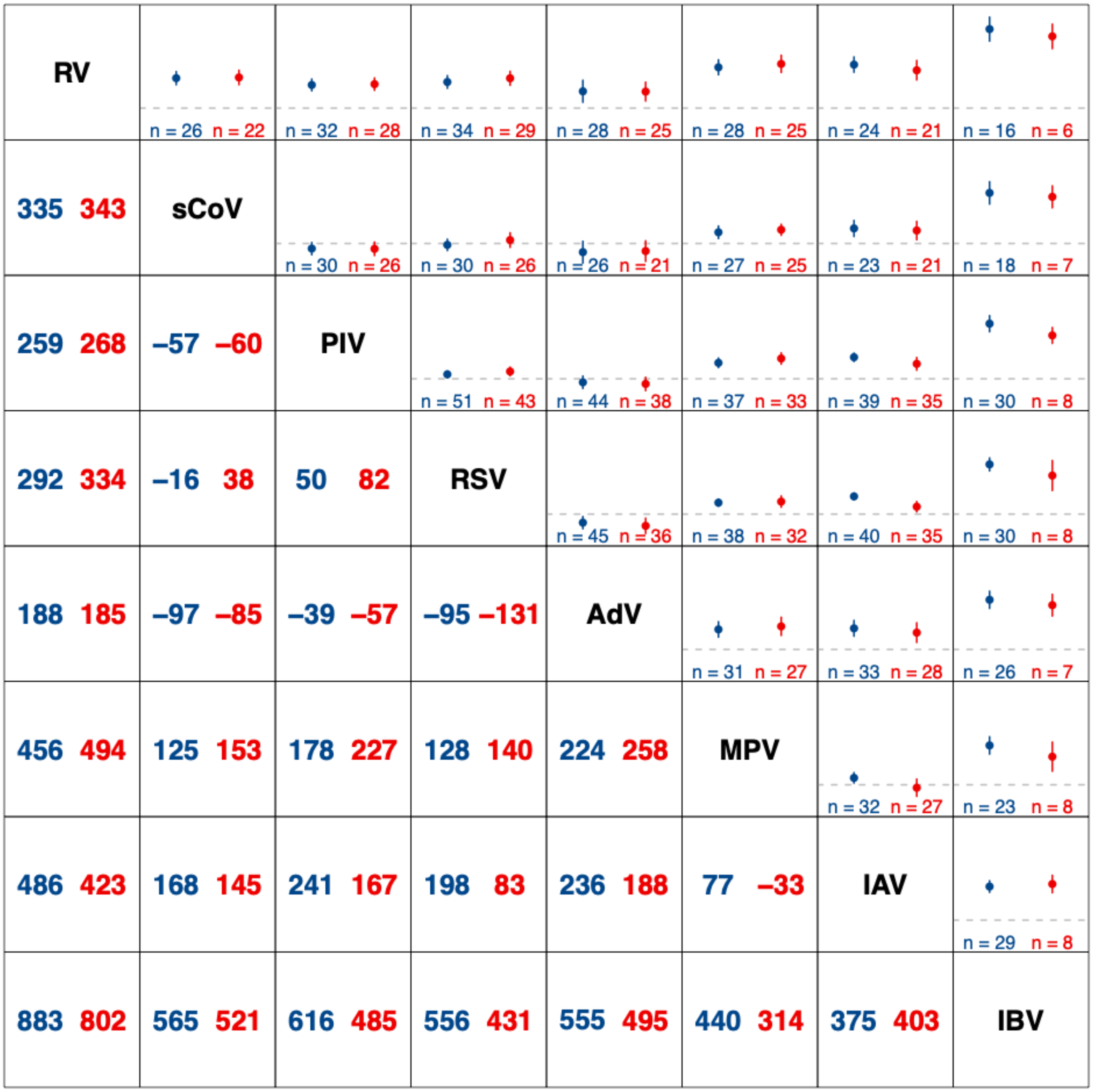


**Supplementary Figure 15. The time difference in days of peak between viruses during their first resurgence and second resurgence following the COVID-19 pandemic onset, matched by study sites in high-income countries.**

The matrix presents the pairwise comparison of timing between viruses in their first (shown in blue) and second resurgence (shown in red). Positive [negative] values indicated that the virus on the top / left resurged earlier [later] than the virus on the bottom / right. Dashed line indicated no differences in the timing. Data are presented as mean values and error bars indicate their 95% confidence intervals. IAV = Influenza A virus; IBV = Influenza B virus; RSV = Respiratory Syncytial Virus; PIV = Parainfluenza Virus; MPV = Metapneumovirus; sCoV = Seasonal Coronavirus; RV = Rhinovirus; AdV = Adenovirus (AdV).

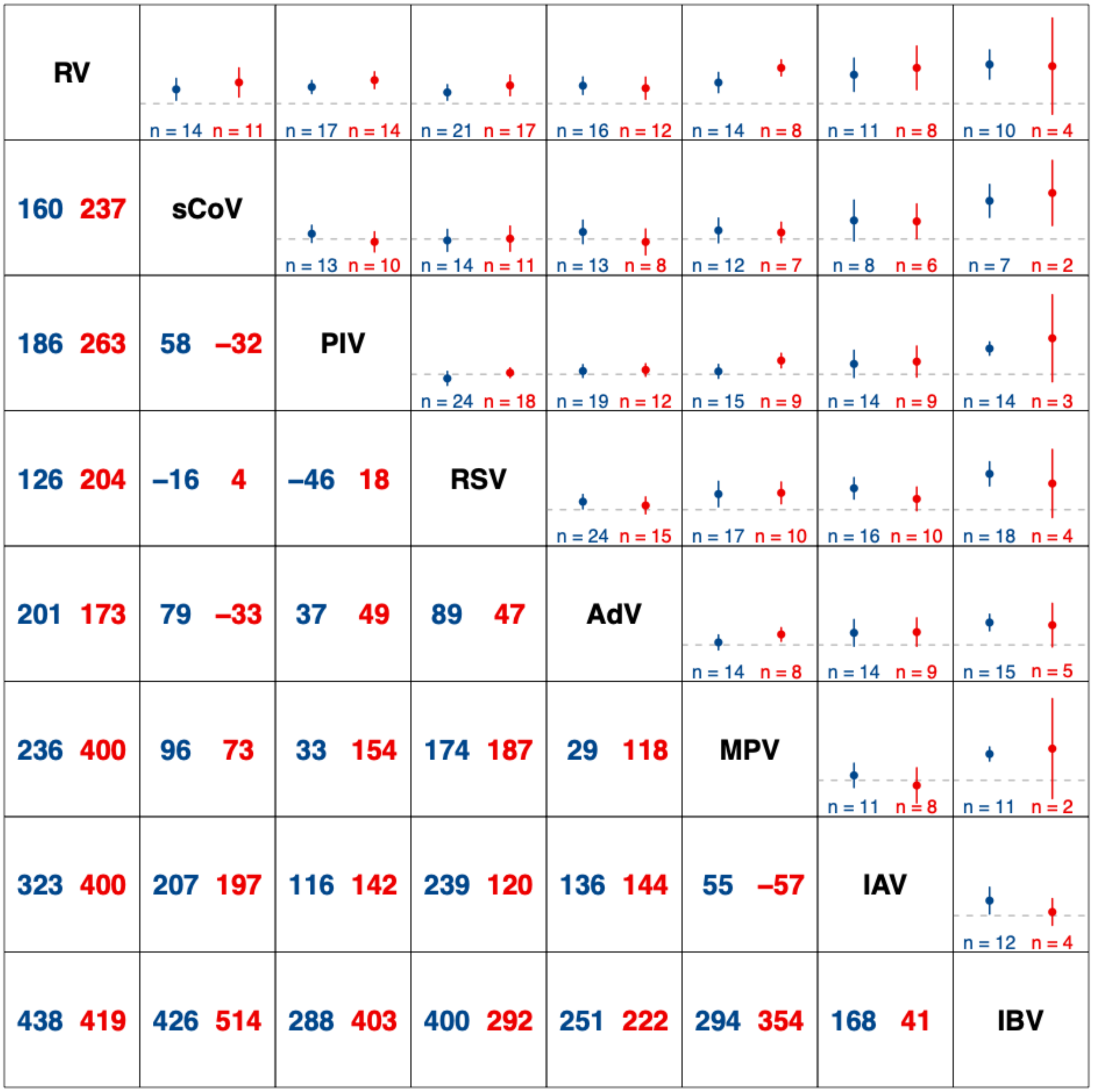


**Supplementary Figure 16. The time difference in days of onset between viruses during their first resurgence and second resurgence following the COVID-19 pandemic onset, matched by sites in non-high-income countries.**

The matrix presents the pairwise comparison of timing between viruses in their first (shown in blue) and second resurgence (shown in red). Positive [negative] values indicated that the virus on the top / left resurged earlier [later] than the virus on the bottom / right. Dashed line indicated no differences in the timing. Data are presented as mean values and error bars indicate their 95% confidence intervals. IAV = Influenza A virus; IBV = Influenza B virus; RSV = Respiratory Syncytial Virus; PIV = Parainfluenza Virus; MPV = Metapneumovirus; sCoV = Seasonal Coronavirus; RV = Rhinovirus; AdV = Adenovirus (AdV).

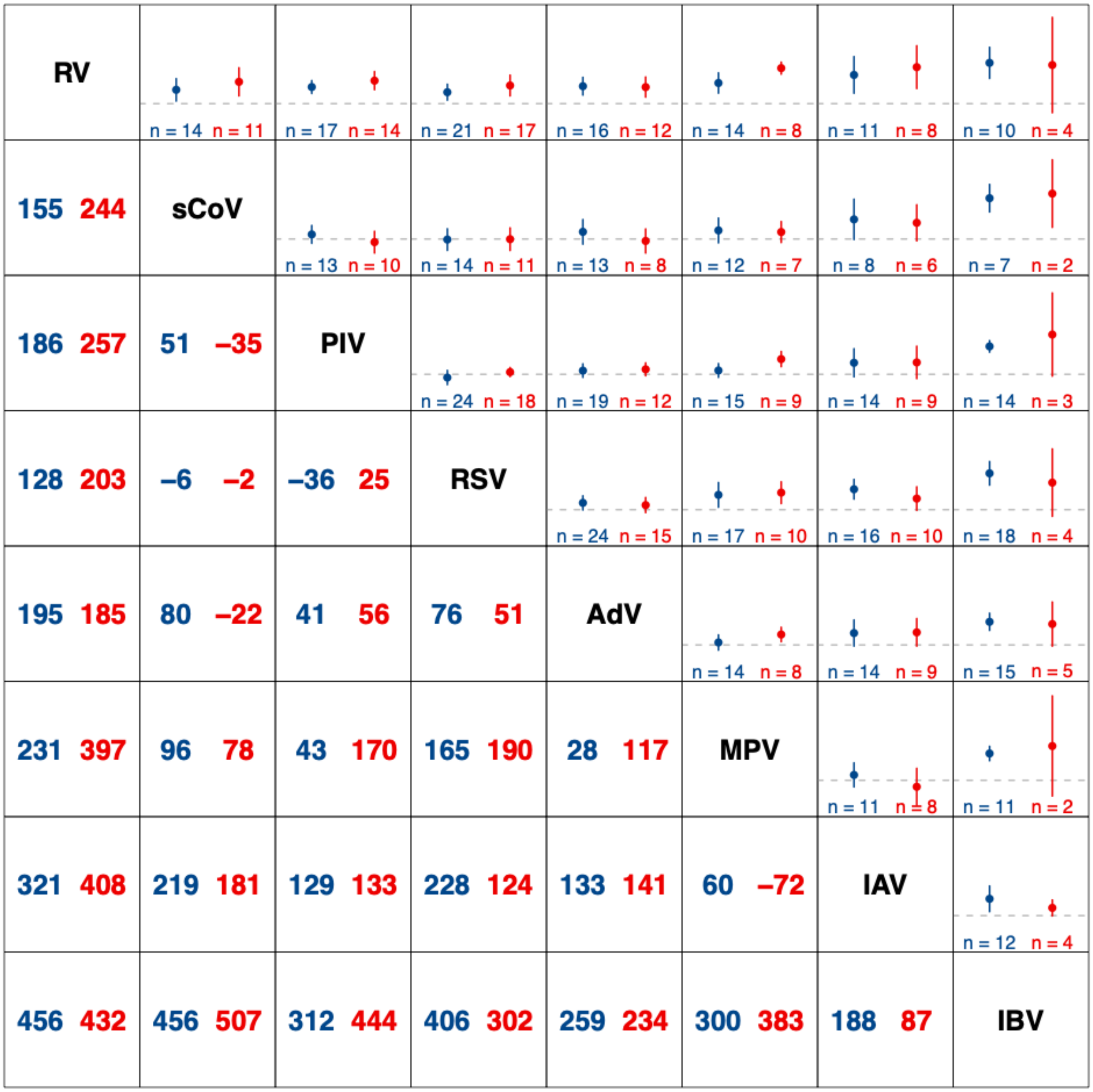


**Supplementary Figure 17. The time difference in days of peak between viruses during their first resurgence and second resurgence following the COVID-19 pandemic onset, matched by study sites in non-high-income countries.**

The matrix presents the pairwise comparison of timing between viruses in their first (shown in blue) and second resurgence (shown in red). Positive [negative] values indicated that the virus on the top / left resurged earlier [later] than the virus on the bottom / right. Dashed line indicated no differences in the timing. Data are presented as mean values and error bars indicate their 95% confidence intervals. IAV = Influenza A virus; IBV = Influenza B virus; RSV = Respiratory Syncytial Virus; PIV = Parainfluenza Virus; MPV = Metapneumovirus; sCoV = Seasonal Coronavirus; RV = Rhinovirus; AdV = Adenovirus (AdV).

**Sensitivity analysis result**

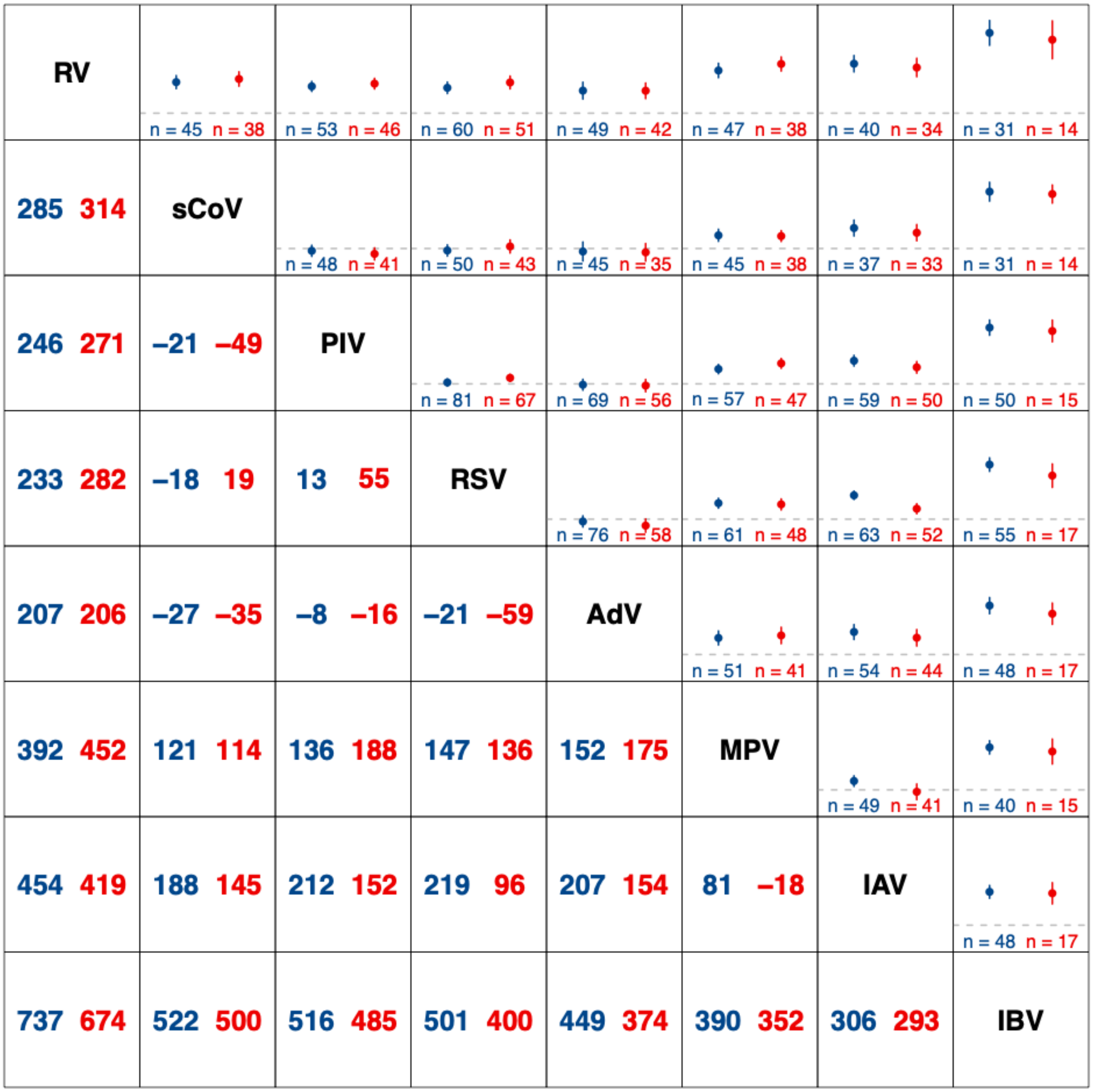


**Supplementary Figure 18. The time difference in days of onset between viruses during their first resurgence and second resurgence following the COVID-19 pandemic onset, matched by study sites reporting positive proportion data.**

The matrix presents the pairwise comparison of timing between viruses in their first (shown in blue) and second resurgence (shown in red). Positive [negative] values indicated that the virus on the top / left resurged earlier [later] than the virus on the bottom / right. Dashed line indicated no differences in the timing. Data are presented as mean values and error bars indicate their 95% confidence intervals. IAV = Influenza A virus; IBV = Influenza B virus; RSV = Respiratory Syncytial Virus; PIV = Parainfluenza Virus; MPV = Metapneumovirus; sCoV = Seasonal Coronavirus; RV = Rhinovirus; AdV = Adenovirus (AdV).

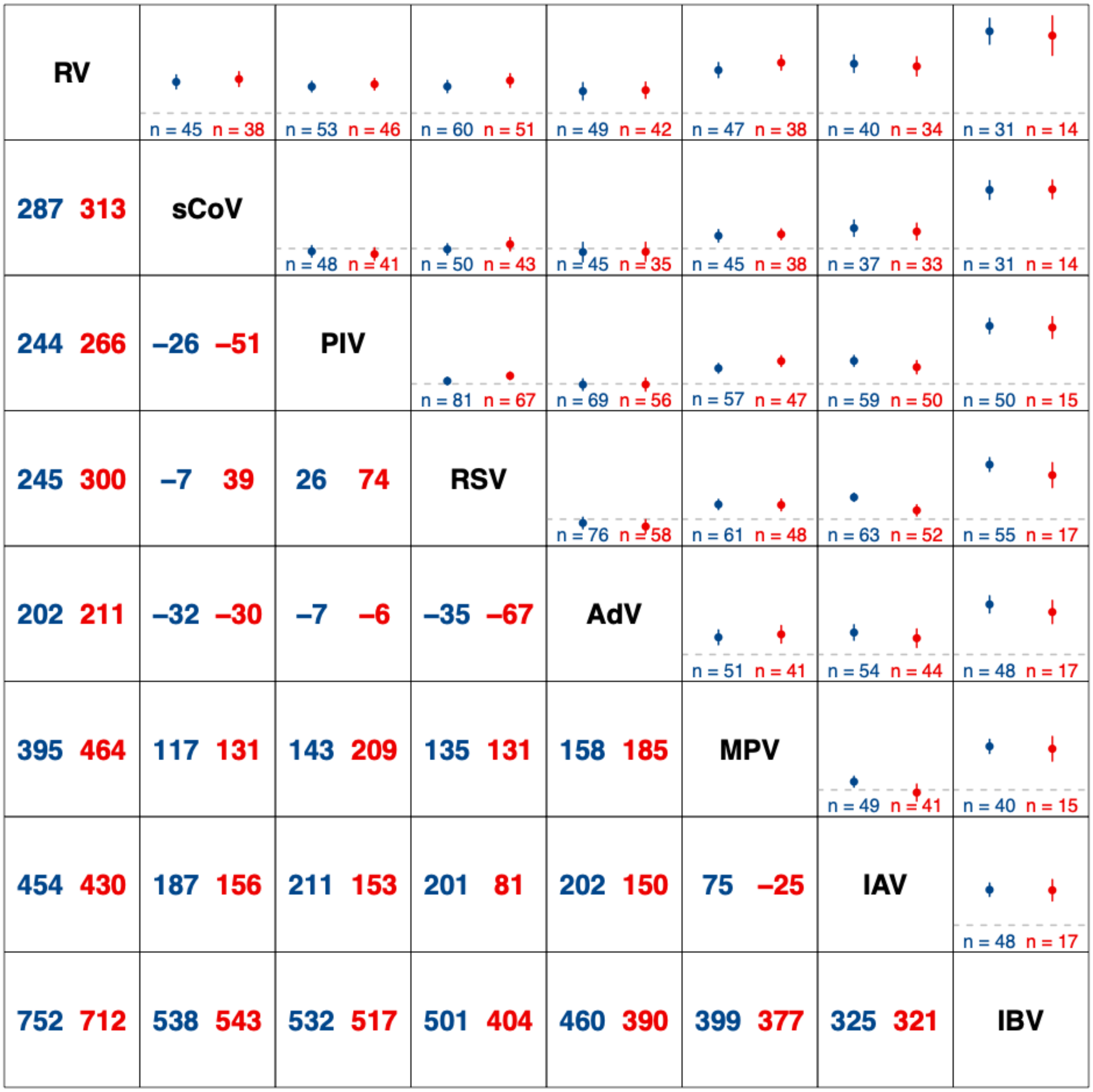


**Supplementary Figure 19. The time difference in days of peak between viruses during their first resurgence and second resurgence following the COVID-19 pandemic onset, matched by study sites reporting positive proportion data.**

The matrix presents the pairwise comparison of timing between viruses in their first (shown in blue) and second resurgence (shown in red). Positive [negative] values indicated that the virus on the top / left resurged earlier [later] than the virus on the bottom / right. Dashed line indicated no differences in the timing. Data are presented as mean values and error bars indicate their 95% confidence intervals. IAV = Influenza A virus; IBV = Influenza B virus; RSV = Respiratory Syncytial Virus; PIV = Parainfluenza Virus; MPV = Metapneumovirus; sCoV = Seasonal Coronavirus; RV = Rhinovirus; AdV = Adenovirus (AdV).

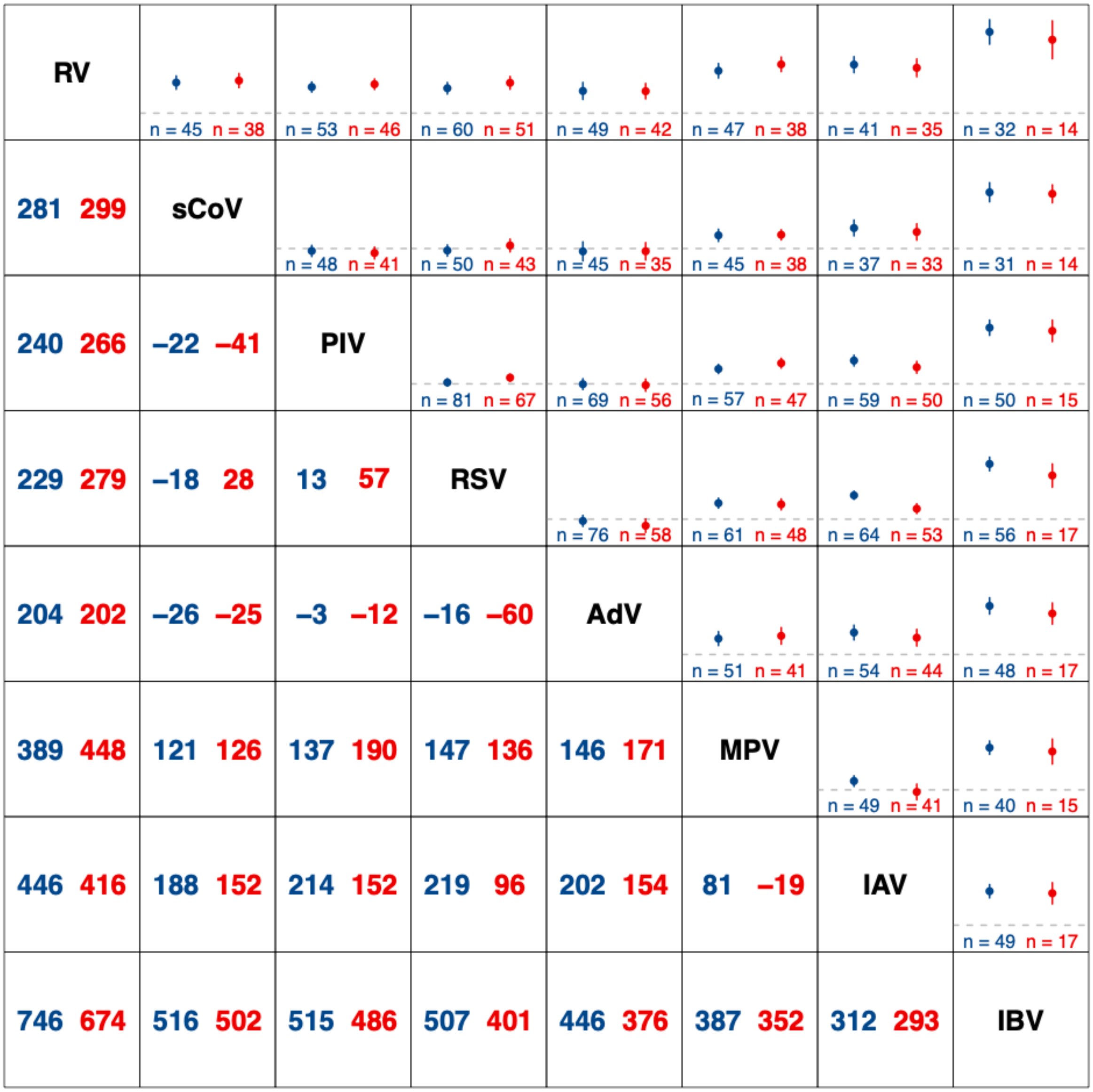


**Supplementary Figure 20. The time difference in days of onset between viruses during their first resurgence and second resurgence following the COVID-19 pandemic onset, matched by study sites reporting positive cases data.**

The matrix presents the pairwise comparison of timing between viruses in their first (shown in blue) and second resurgence (shown in red). Positive [negative] values indicated that the virus on the top / left resurged earlier [later] than the virus on the bottom / right. Dashed line indicated no differences in the timing. Data are presented as mean values and error bars indicate their 95% confidence intervals. IAV = Influenza A virus; IBV = Influenza B virus; RSV = Respiratory Syncytial Virus; PIV = Parainfluenza Virus; MPV = Metapneumovirus; sCoV = Seasonal Coronavirus; RV = Rhinovirus; AdV = Adenovirus (AdV).

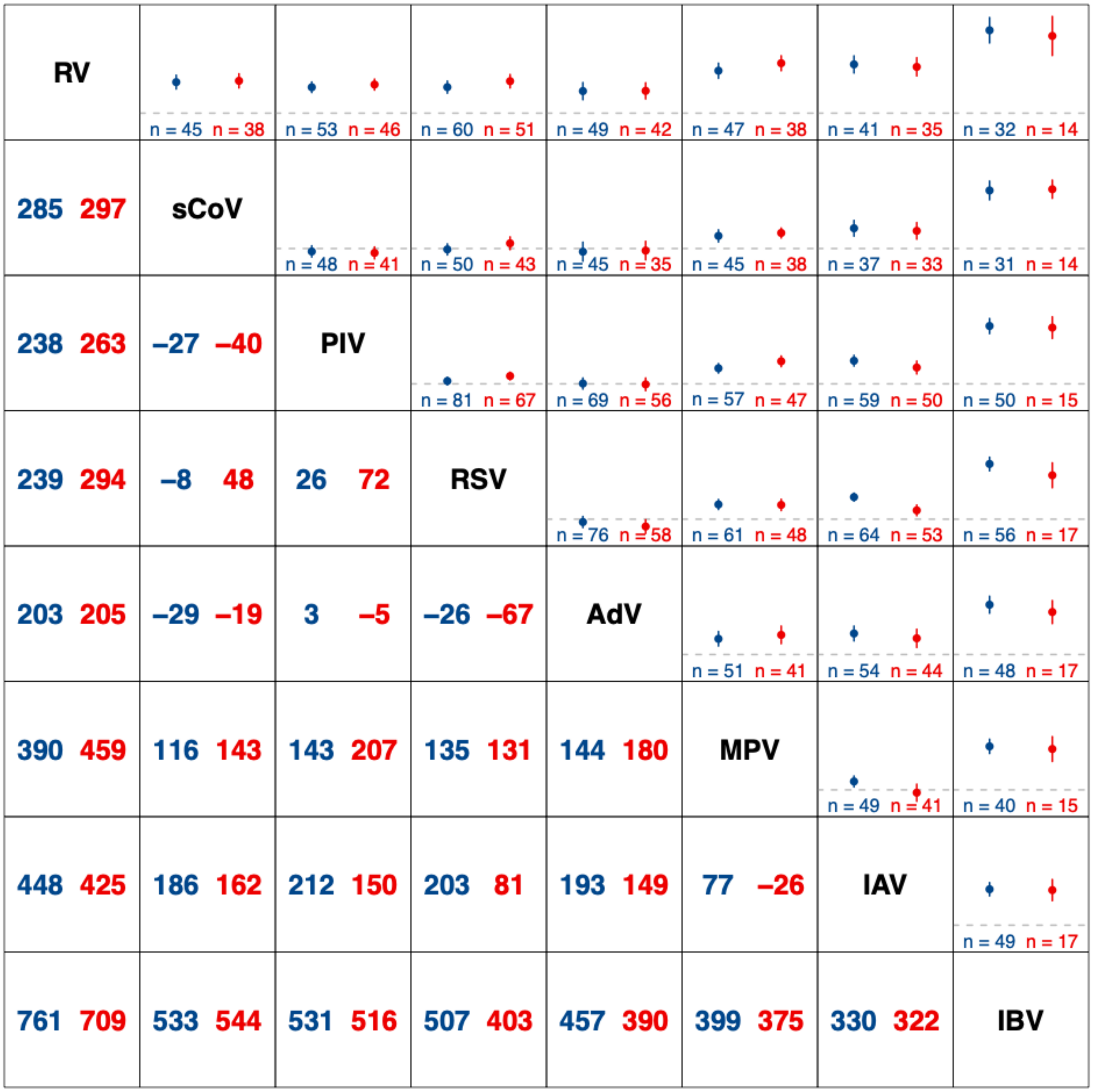


**Supplementary Figure 21. The time difference in days of peak between viruses during their first resurgence and second resurgence following the COVID-19 pandemic onset, matched by study sites reporting positive cases data.**

The matrix presents the pairwise comparison of timing between viruses in their first (shown in blue) and second resurgence (shown in red). Positive [negative] values indicated that the virus on the top / left resurged earlier [later] than the virus on the bottom / right. Dashed line indicated no differences in the timing. Data are presented as mean values and error bars indicate their 95% confidence intervals. IAV = Influenza A virus; IBV = Influenza B virus; RSV = Respiratory Syncytial Virus; PIV = Parainfluenza Virus; MPV = Metapneumovirus; sCoV = Seasonal Coronavirus; RV = Rhinovirus; AdV = Adenovirus (AdV).

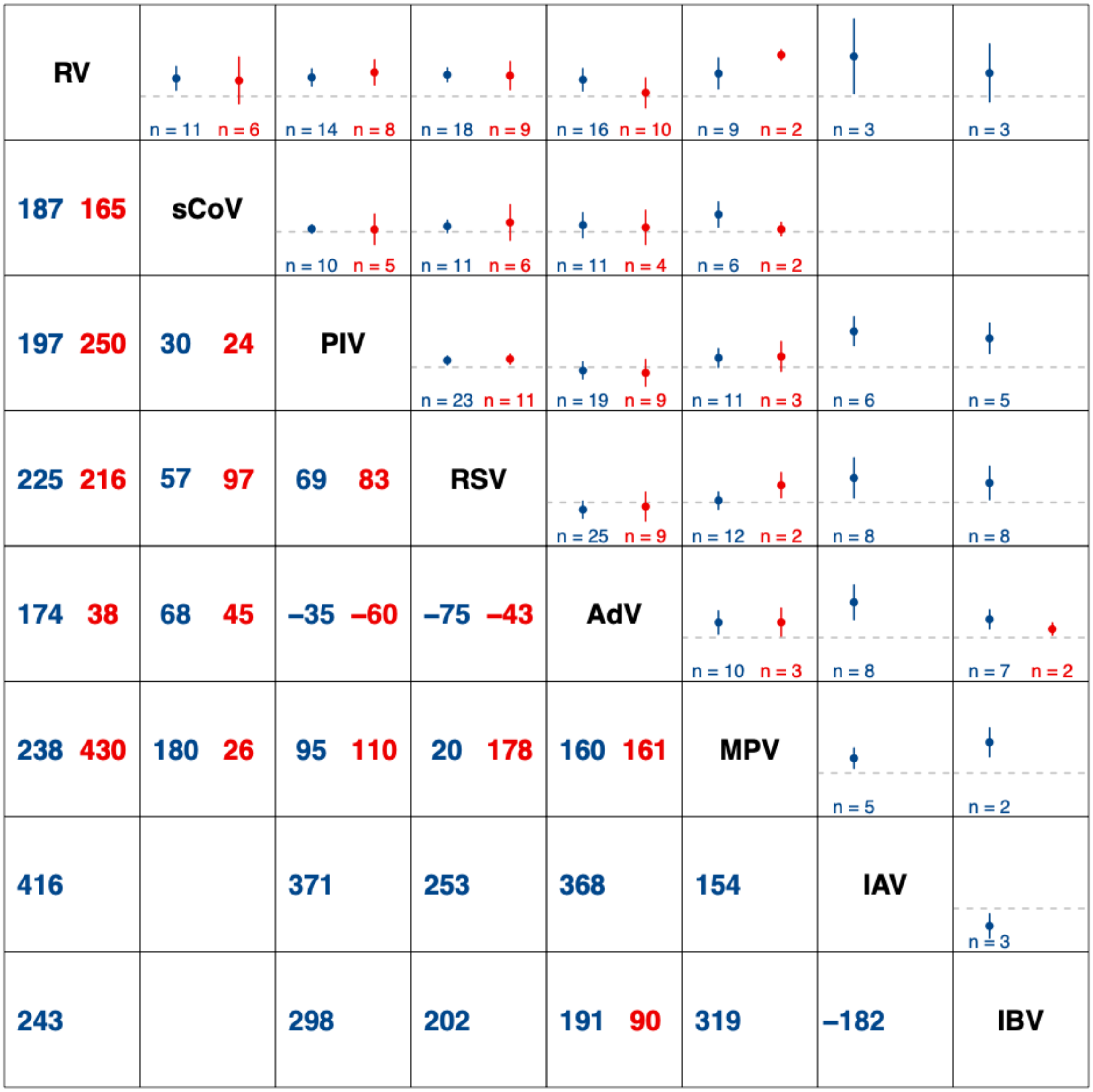


**Supplementary Figure 22. The time difference in days of onset between viruses during their first resurgence and second resurgence following the COVID-19 pandemic onset, matched by study sites using data extracted from systematic literature review.**

The matrix presents the pairwise comparison of timing between viruses in their first (shown in blue) and second resurgence (shown in red). Positive [negative] values indicated that the virus on the top / left resurged earlier [later] than the virus on the bottom / right. Dashed line indicated no differences in the timing. Data are presented as mean values and error bars indicate their 95% confidence intervals. IAV = Influenza A virus; IBV = Influenza B virus; RSV = Respiratory Syncytial Virus; PIV = Parainfluenza Virus; MPV = Metapneumovirus; sCoV = Seasonal Coronavirus; RV = Rhinovirus; AdV = Adenovirus (AdV).

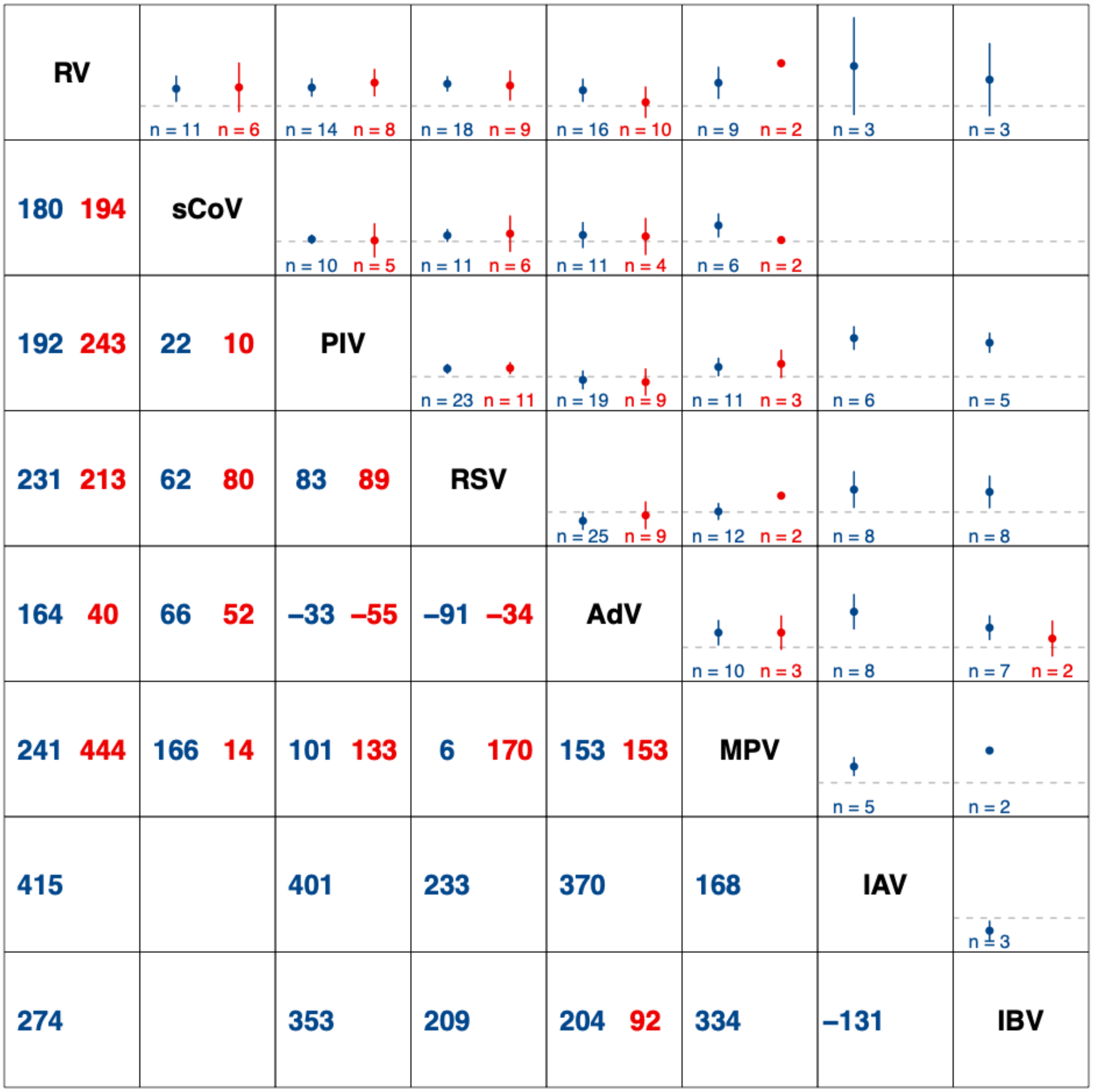


**Supplementary Figure 23. The time difference in days of peak between viruses during their first resurgence and second resurgence following the COVID-19 pandemic onset, matched by study sites using data extracted from systematic literature review.**

The matrix presents the pairwise comparison of timing between viruses in their first (shown in blue) and second resurgence (shown in red). Positive [negative] values indicated that the virus on the top / left resurged earlier [later] than the virus on the bottom / right. Dashed line indicated no differences in the timing. Data are presented as mean values and error bars indicate their 95% confidence intervals. IAV = Influenza A virus; IBV = Influenza B virus; RSV = Respiratory Syncytial Virus; PIV = Parainfluenza Virus; MPV = Metapneumovirus; sCoV = Seasonal Coronavirus; RV = Rhinovirus; AdV = Adenovirus (AdV).

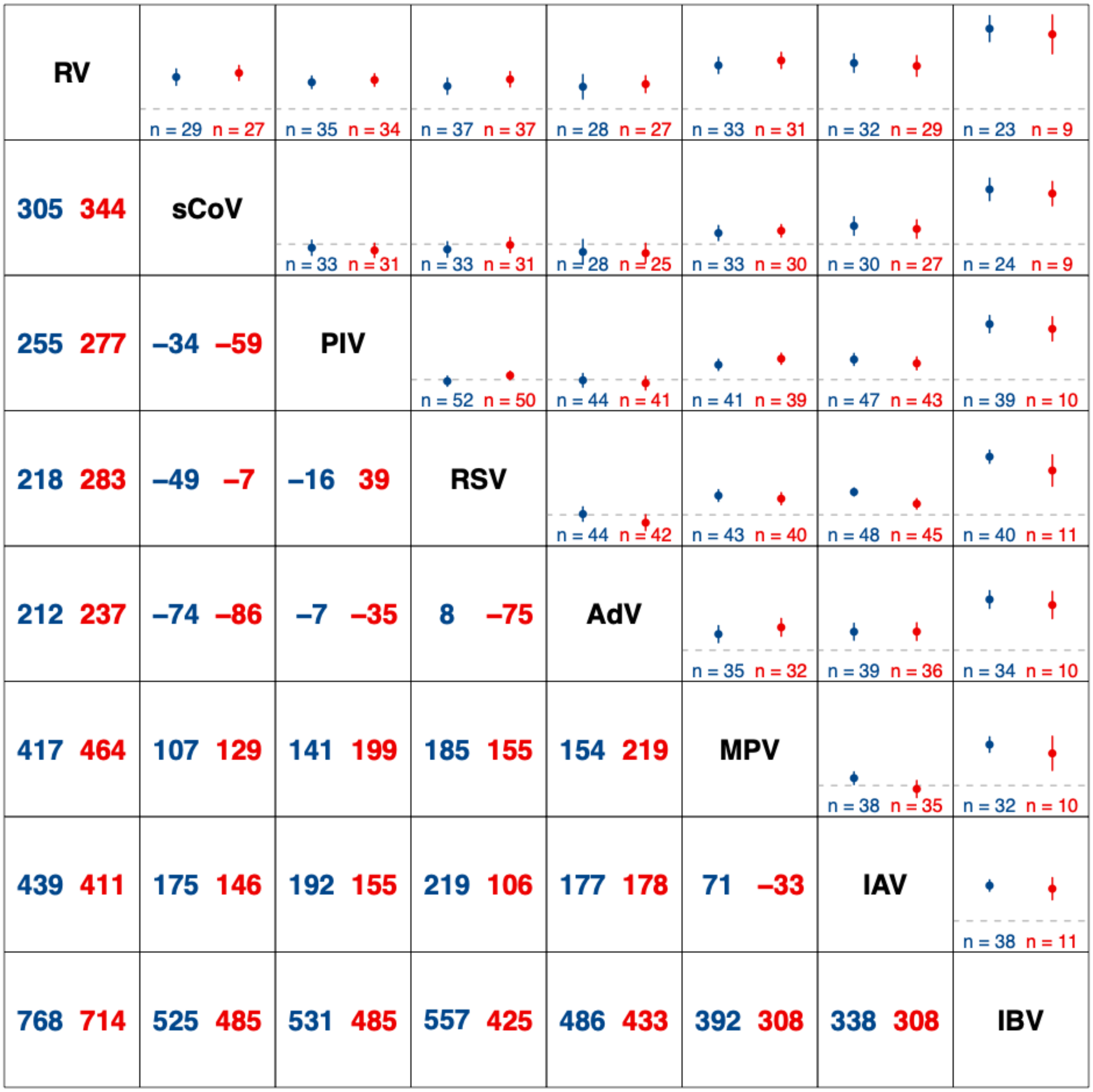


**Supplementary Figure 24. The time difference in days of onset between viruses during their first resurgence and second resurgence following the COVID-19 pandemic onset, matched by study sites using data extracted from databases.**

The matrix presents the pairwise comparison of timing between viruses in their first (shown in blue) and second resurgence (shown in red). Positive [negative] values indicated that the virus on the top / left resurged earlier [later] than the virus on the bottom / right. Dashed line indicated no differences in the timing. Data are presented as mean values and error bars indicate their 95% confidence intervals. IAV = Influenza A virus; IBV = Influenza B virus; RSV = Respiratory Syncytial Virus; PIV = Parainfluenza Virus; MPV = Metapneumovirus; sCoV = Seasonal Coronavirus; RV = Rhinovirus; AdV = Adenovirus (AdV).

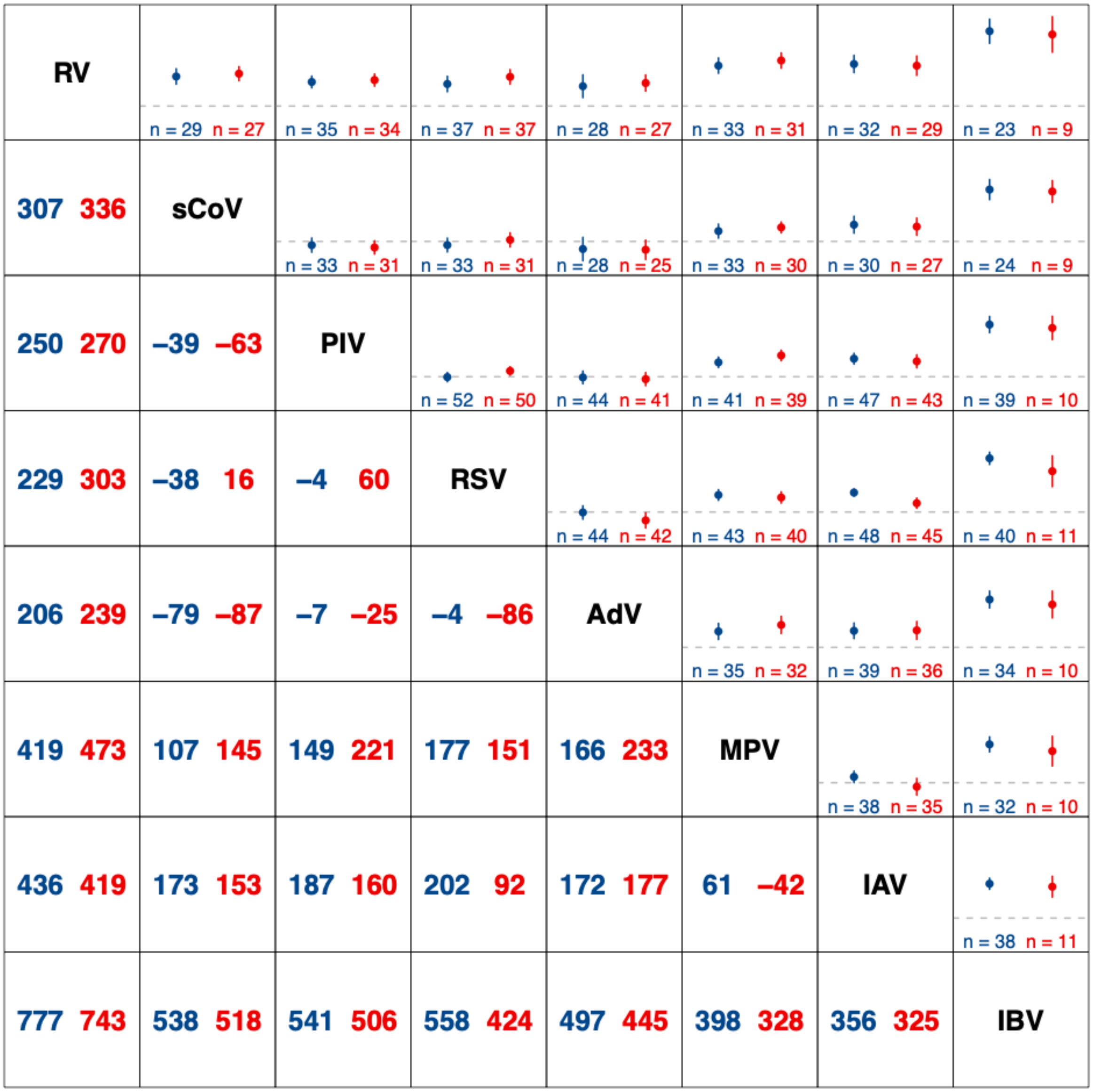


**Supplementary Figure 25.The time difference in days of peak between viruses during their first resurgence and second resurgence following the COVID-19 pandemic onset, matched by study sites using data extracted from databases.**

The matrix presents the pairwise comparison of timing between viruses in their first (shown in blue) and second resurgence (shown in red). Positive [negative] values indicated that the virus on the top / left resurged earlier [later] than the virus on the bottom / right. Dashed line indicated no differences in the timing. Data are presented as mean values and error bars indicate their 95% confidence intervals. IAV = Influenza A virus; IBV = Influenza B virus; RSV = Respiratory Syncytial Virus; PIV = Parainfluenza Virus; MPV = Metapneumovirus; sCoV = Seasonal Coronavirus; RV = Rhinovirus; AdV = Adenovirus (AdV).

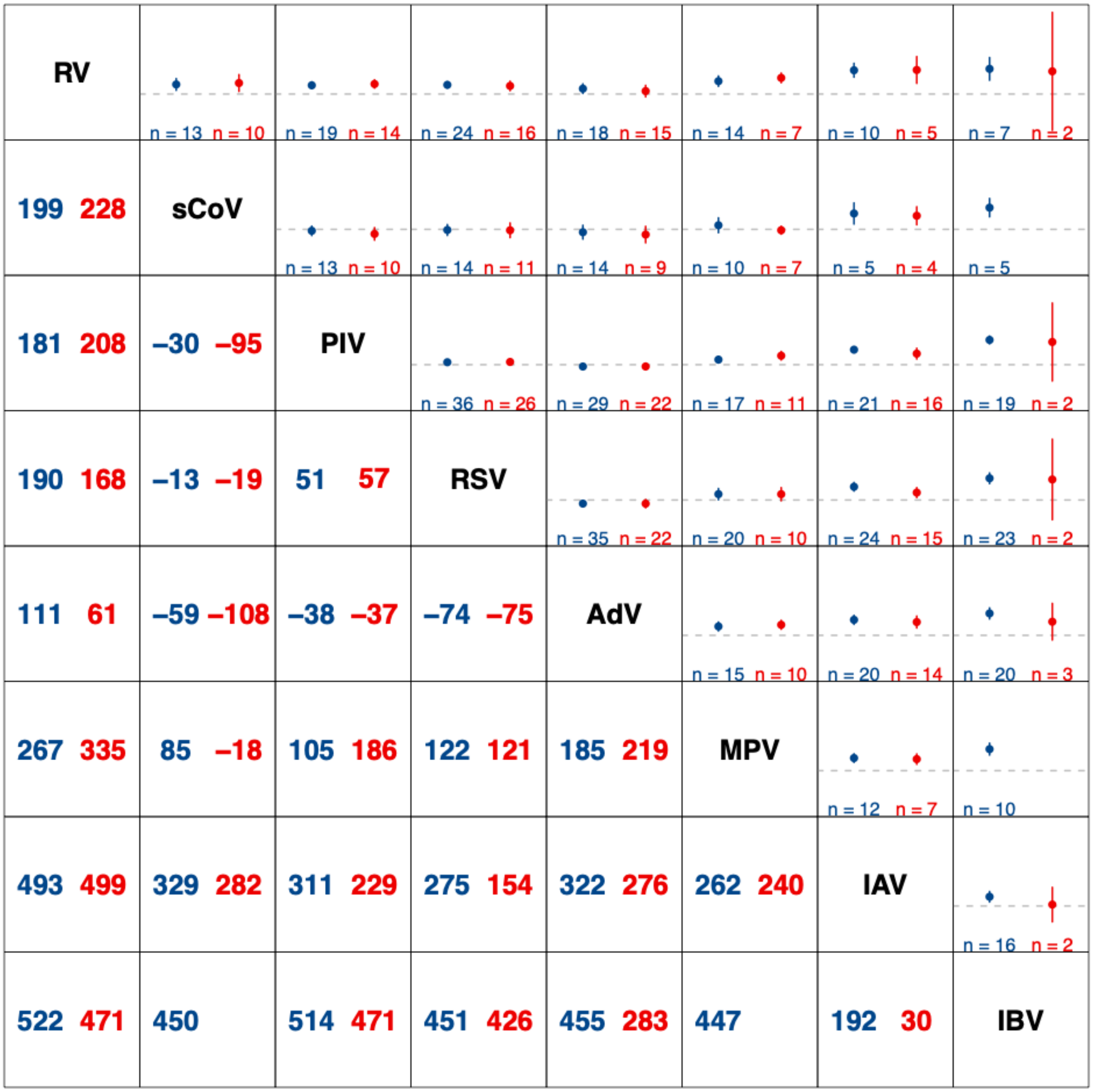


**Supplementary Figure 26. The time difference in days of onset between viruses during their first resurgence and second resurgence following the COVID-19 pandemic onset, matched by study sites reporting monthly aggregated viral activity data.**

The matrix presents the pairwise comparison of timing between viruses in their first (shown in blue) and second resurgence (shown in red). Positive [negative] values indicated that the virus on the top / left resurged earlier [later] than the virus on the bottom / right. Dashed line indicated no differences in the timing. Data are presented as mean values and error bars indicate their 95% confidence intervals. IAV = Influenza A virus; IBV = Influenza B virus; RSV = Respiratory Syncytial Virus; PIV = Parainfluenza Virus; MPV = Metapneumovirus; sCoV = Seasonal Coronavirus; RV = Rhinovirus; AdV = Adenovirus (AdV).

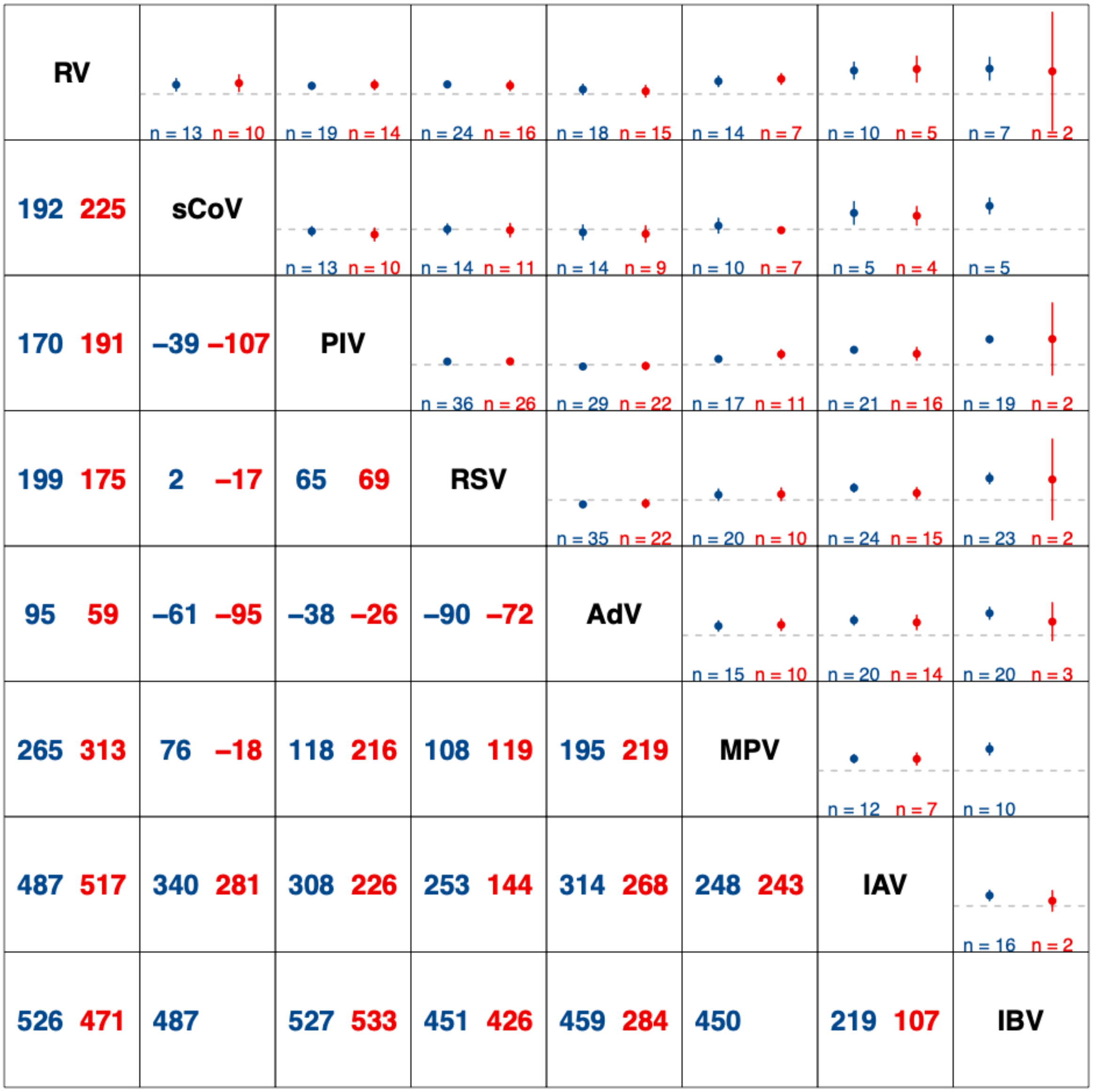


**Supplementary Figure 27. The time difference in days of peak between viruses during their first resurgence and second resurgence following the COVID-19 pandemic onset, matched by study sites reporting monthly aggregated viral activity data.**

The matrix presents the pairwise comparison of timing between viruses in their first (shown in blue) and second resurgence (shown in red). Positive [negative] values indicated that the virus on the top / left resurged earlier [later] than the virus on the bottom / right. Dashed line indicated no differences in the timing. Data are presented as mean values and error bars indicate their 95% confidence intervals. IAV = Influenza A virus; IBV = Influenza B virus; RSV = Respiratory Syncytial Virus; PIV = Parainfluenza Virus; MPV = Metapneumovirus; sCoV = Seasonal Coronavirus; RV = Rhinovirus; AdV = Adenovirus (AdV).

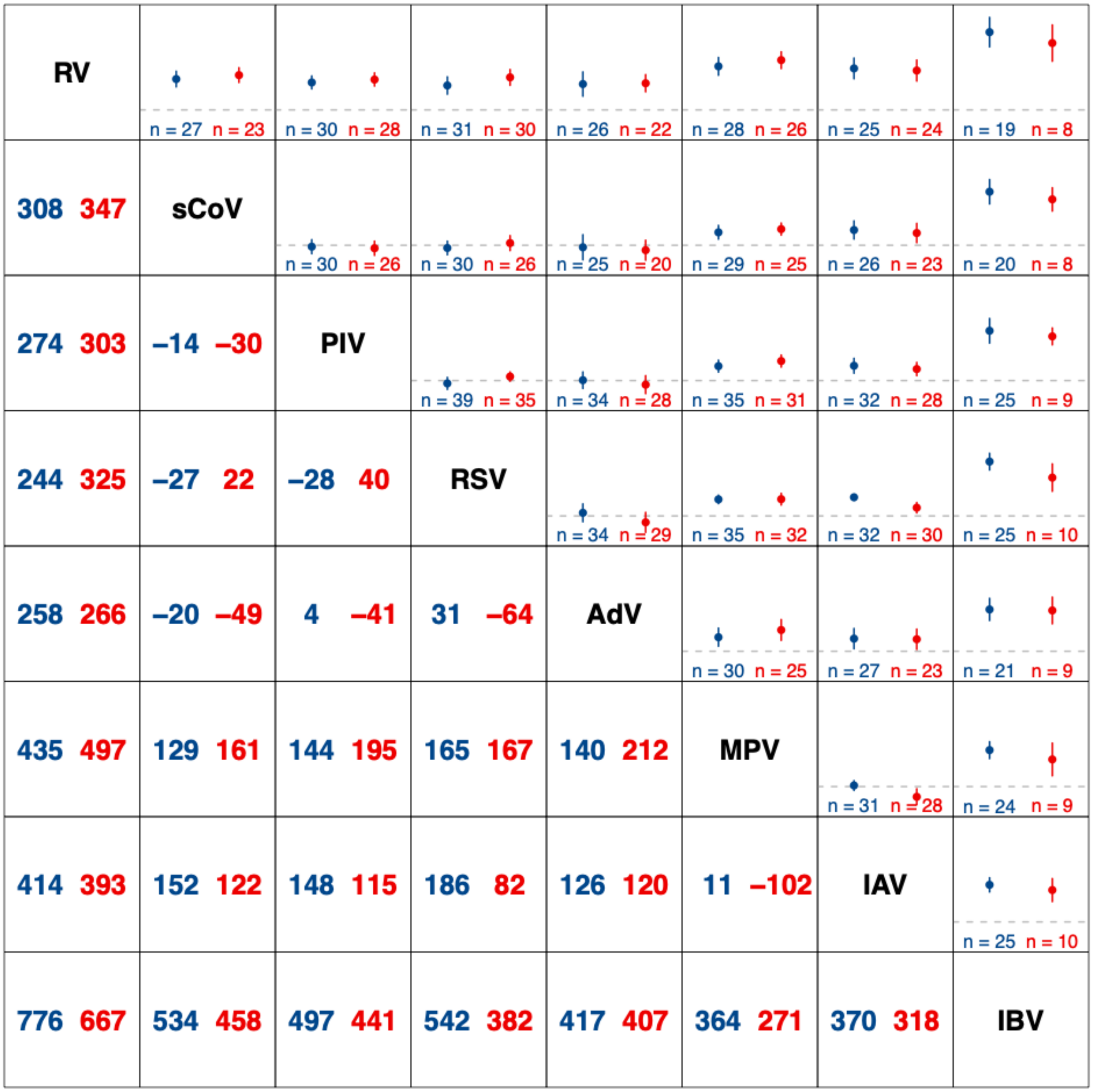


**Supplementary Figure 28. The time difference in days of onset between viruses during their first resurgence and second resurgence following the COVID-19 pandemic onset, matched by study sites reporting weekly aggregated viral activity data.**

The matrix presents the pairwise comparison of timing between viruses in their first (shown in blue) and second resurgence (shown in red). Positive [negative] values indicated that the virus on the top / left resurged earlier [later] than the virus on the bottom / right. Dashed line indicated no differences in the timing. Data are presented as mean values and error bars indicate their 95% confidence intervals. IAV = Influenza A virus; IBV = Influenza B virus; RSV = Respiratory Syncytial Virus; PIV = Parainfluenza Virus; MPV = Metapneumovirus; sCoV = Seasonal Coronavirus; RV = Rhinovirus; AdV = Adenovirus (AdV).

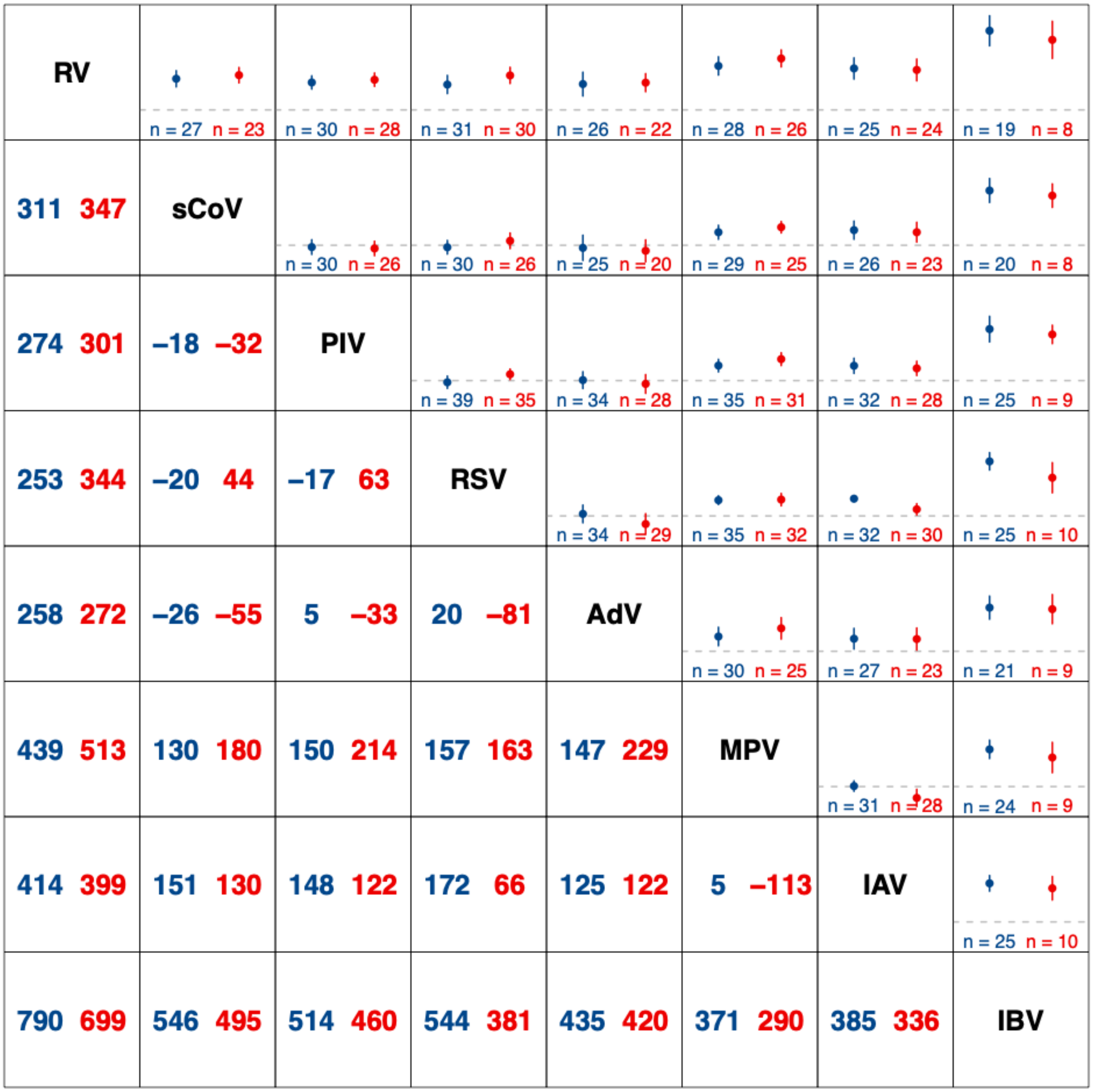


**Supplementary Figure 29. The time difference in days of peak between viruses during their first resurgence and second resurgence following the COVID-19 pandemic onset, matched by study sites reporting weekly aggregated viral activity data.**

The matrix presents the pairwise comparison of timing between viruses in their first (shown in blue) and second resurgence (shown in red). Positive [negative] values indicated that the virus on the top / left resurged earlier [later] than the virus on the bottom / right. Dashed line indicated no differences in the timing. Data are presented as mean values and error bars indicate their 95% confidence intervals. IAV = Influenza A virus; IBV = Influenza B virus; RSV = Respiratory Syncytial Virus; PIV = Parainfluenza Virus; MPV = Metapneumovirus; sCoV = Seasonal Coronavirus; RV = Rhinovirus; AdV = Adenovirus (AdV).

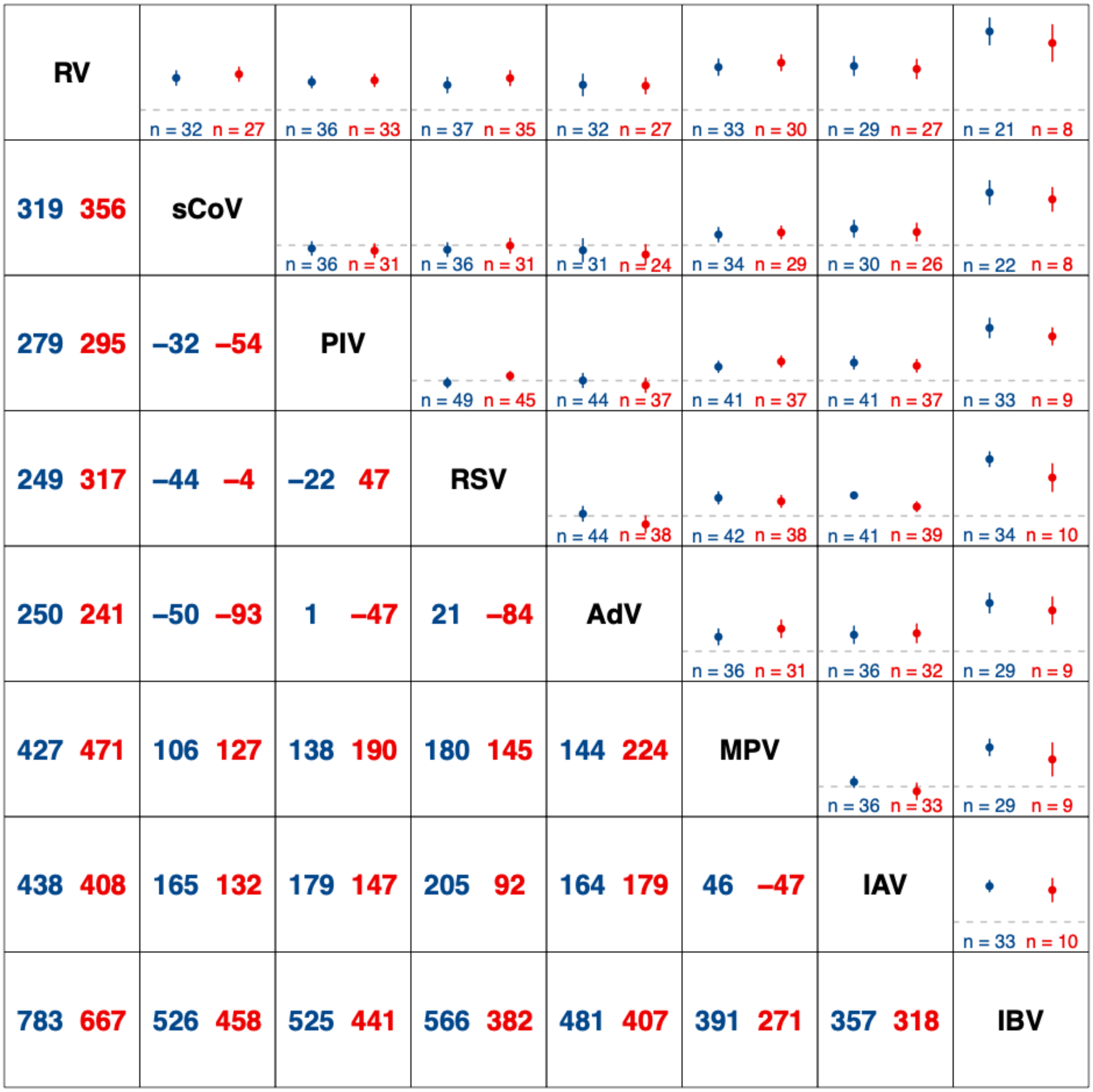


**Supplementary Figure 30. The time difference in days of onset between viruses during their first resurgence and second resurgence following the COVID-19 pandemic onset, matched by study sites providing moderate-to-high quality data.**

The matrix presents the pairwise comparison of timing between viruses in their first (shown in blue) and second resurgence (shown in red). Positive [negative] values indicated that the virus on the top / left resurged earlier [later] than the virus on the bottom / right. Dashed line indicated no differences in the timing. Data are presented as mean values and error bars indicate their 95% confidence intervals. IAV = Influenza A virus; IBV = Influenza B virus; RSV = Respiratory Syncytial Virus; PIV = Parainfluenza Virus; MPV = Metapneumovirus; sCoV = Seasonal Coronavirus; RV = Rhinovirus; AdV = Adenovirus (AdV).

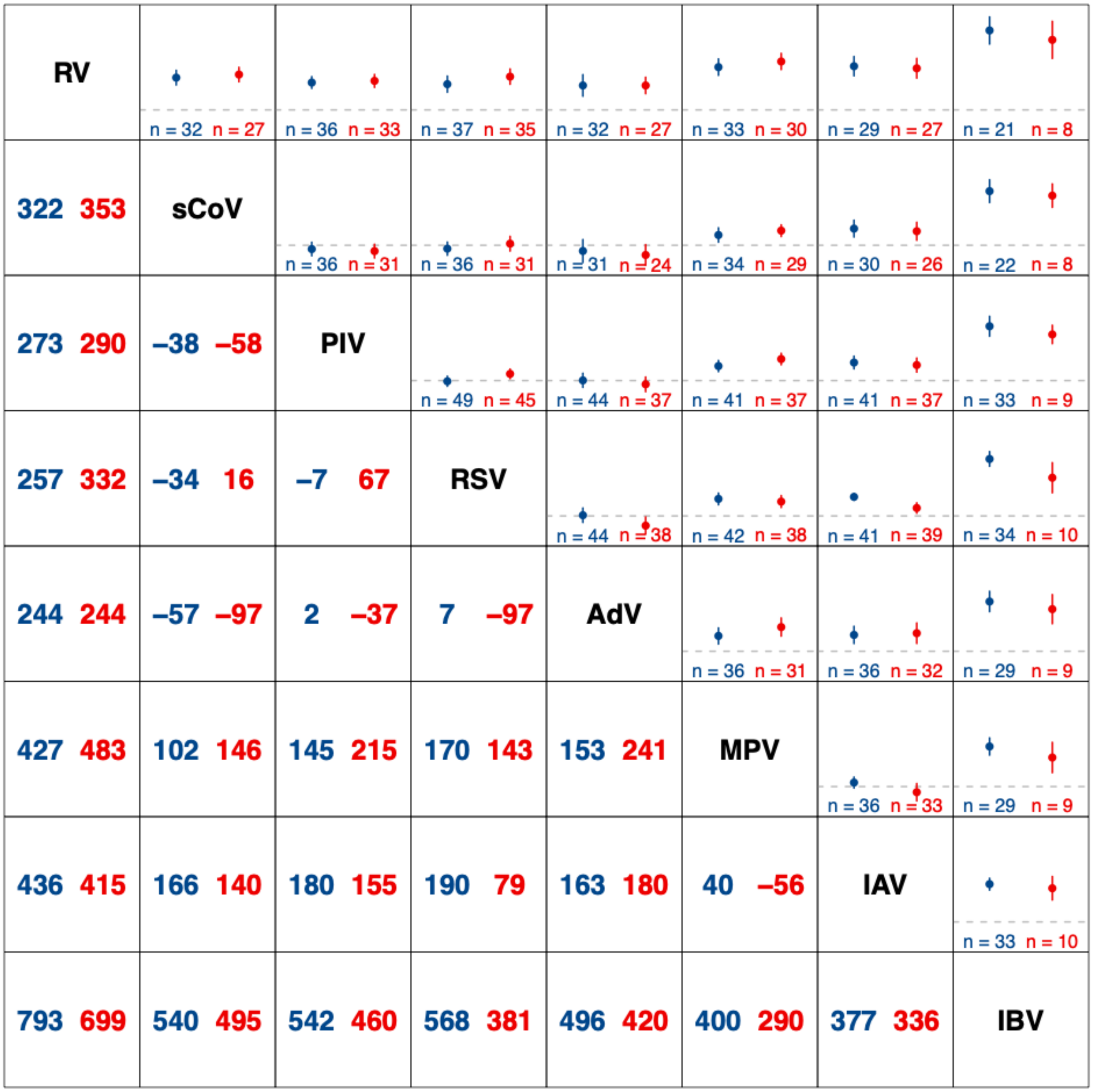


**Supplementary Figure 31. The time difference in days of peak between viruses during their first resurgence and second resurgence following the COVID-19 pandemic onset, matched by study sites providing moderate-to-high quality data.**

The matrix presents the pairwise comparison of timing between viruses in their first (shown in blue) and second resurgence (shown in red). Positive [negative] values indicated that the virus on the top / left resurged earlier [later] than the virus on the bottom / right. Dashed line indicated no differences in the timing. Data are presented as mean values and error bars indicate their 95% confidence intervals. IAV = Influenza A virus; IBV = Influenza B virus; RSV = Respiratory Syncytial Virus; PIV = Parainfluenza Virus; MPV = Metapneumovirus; sCoV = Seasonal Coronavirus; RV = Rhinovirus; AdV = Adenovirus (AdV).

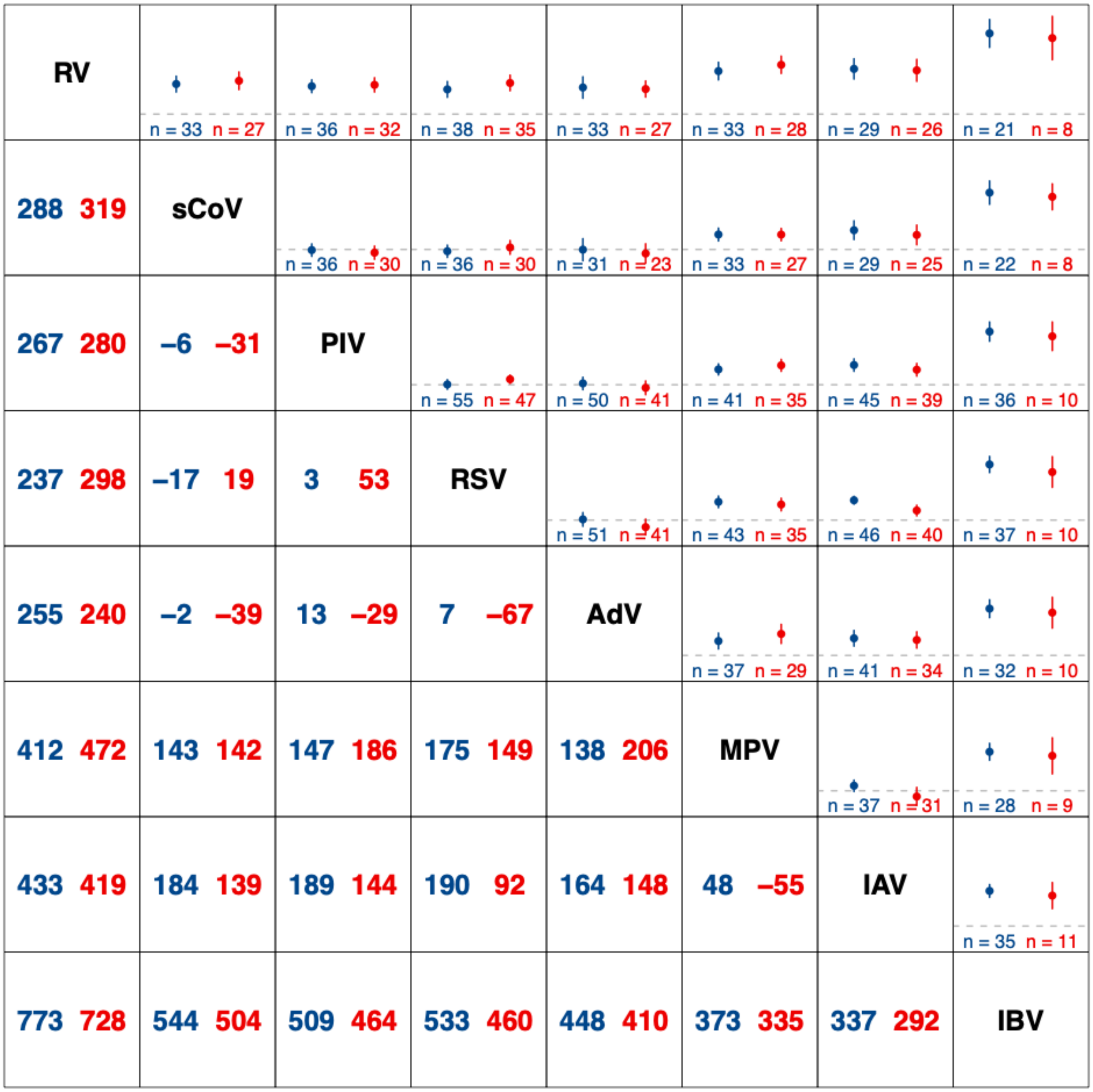


**Supplementary Figure 32. The time difference in days of onset between viruses during their first resurgence and second resurgence following the COVID-19 pandemic onset, matched by study sites providing all age group data.**

The matrix presents the pairwise comparison of timing between viruses in their first (shown in blue) and second resurgence (shown in red). Positive [negative] values indicated that the virus on the top / left resurged earlier [later] than the virus on the bottom / right. Dashed line indicated no differences in the timing. Data are presented as mean values and error bars indicate their 95% confidence intervals. IAV = Influenza A virus; IBV = Influenza B virus; RSV = Respiratory Syncytial Virus; PIV = Parainfluenza Virus; MPV = Metapneumovirus; sCoV = Seasonal Coronavirus; RV = Rhinovirus; AdV = Adenovirus (AdV).

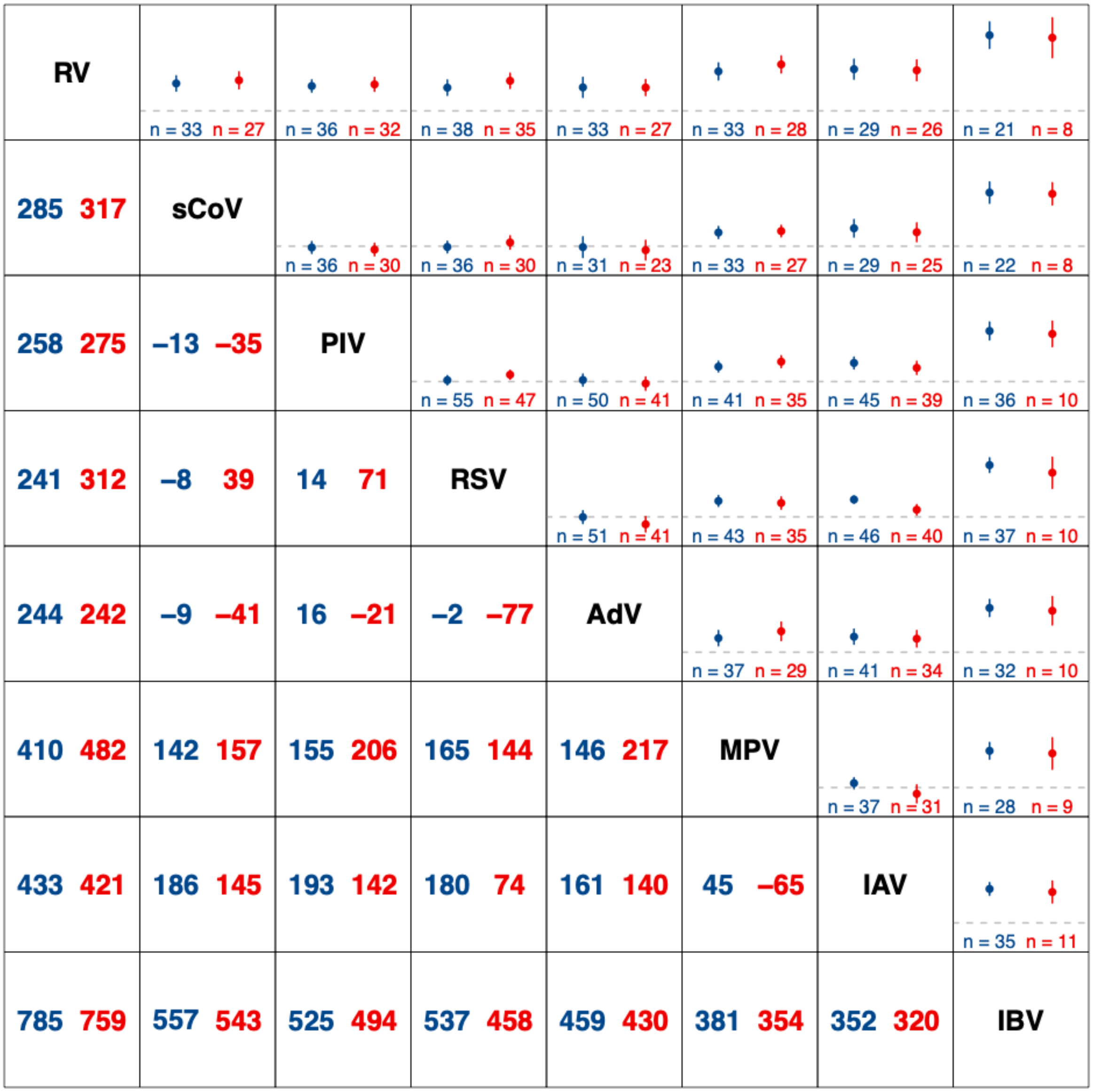


**Supplementary Figure 33. The time difference in days of peak between viruses during their first resurgence and second resurgence following the COVID-19 pandemic onset, matched by study sites providing all age group data.**

The matrix presents the pairwise comparison of timing between viruses in their first (shown in blue) and second resurgence (shown in red). Positive [negative] values indicated that the virus on the top / left resurged earlier [later] than the virus on the bottom / right. Dashed line indicated no differences in the timing. Data are presented as mean values and error bars indicate their 95% confidence intervals. IAV = Influenza A virus; IBV = Influenza B virus; RSV = Respiratory Syncytial Virus; PIV = Parainfluenza Virus; MPV = Metapneumovirus; sCoV = Seasonal Coronavirus; RV = Rhinovirus; AdV = Adenovirus (AdV).

**Ad-hoc analysis results**

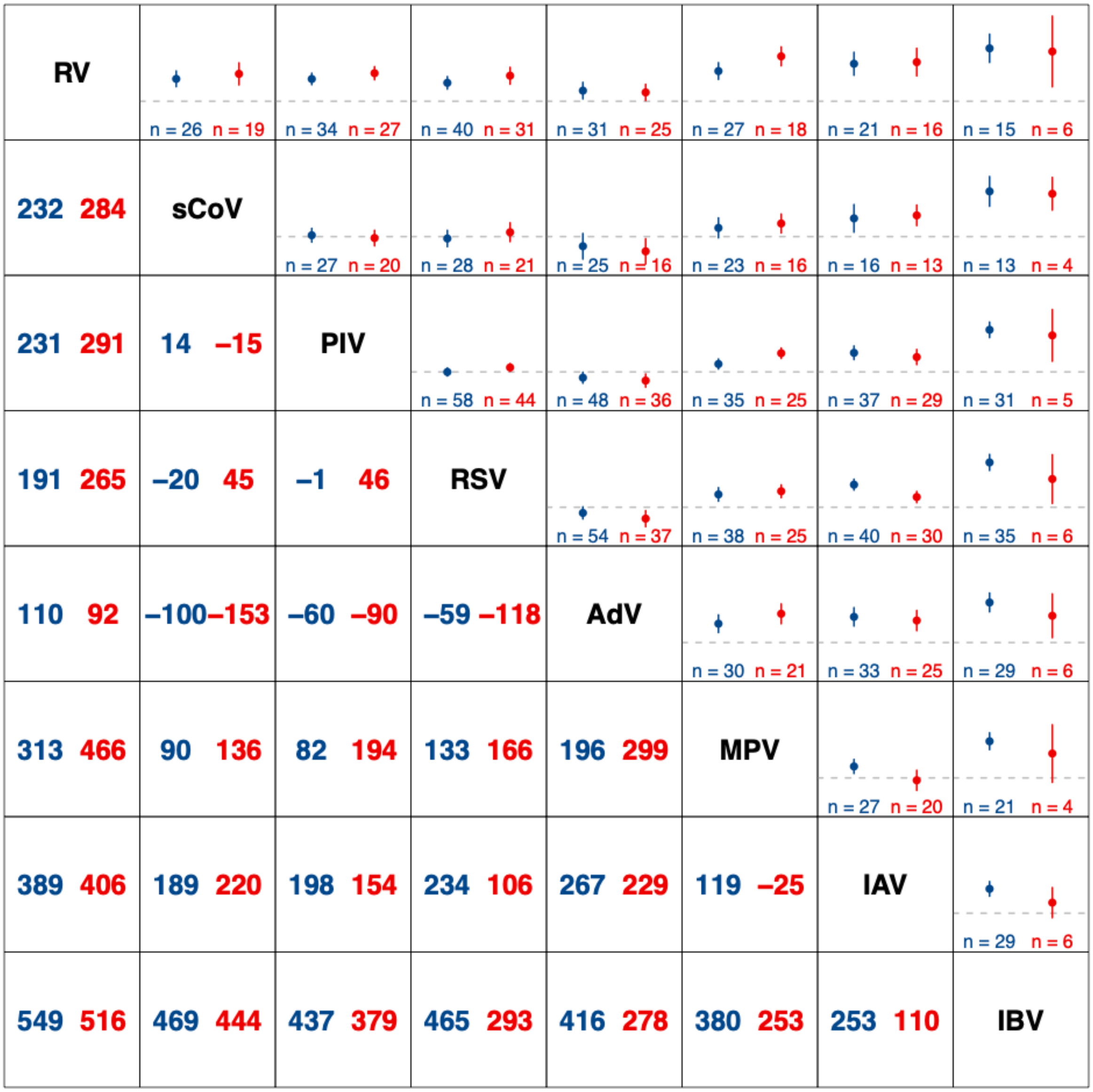


**Supplementary Figure 34. The time difference in days of onset between viruses during their first resurgence and second resurgence following the COVID-19 pandemic onset in study sites excluding Canadian data.**

The matrix presents the pairwise comparison of timing between viruses in their first (shown in blue) and second resurgence (shown in red). Positive [negative] values indicated that the virus on the top / left resurged earlier [later] than the virus on the bottom / right. Dashed line indicated no differences in the timing. Data are presented as mean values and error bars indicate their 95% confidence intervals. IAV = Influenza A virus; IBV = Influenza B virus; RSV = Respiratory Syncytial Virus; PIV = Parainfluenza Virus; MPV = Metapneumovirus; sCoV = Seasonal Coronavirus; RV = Rhinovirus; AdV = Adenovirus (AdV).

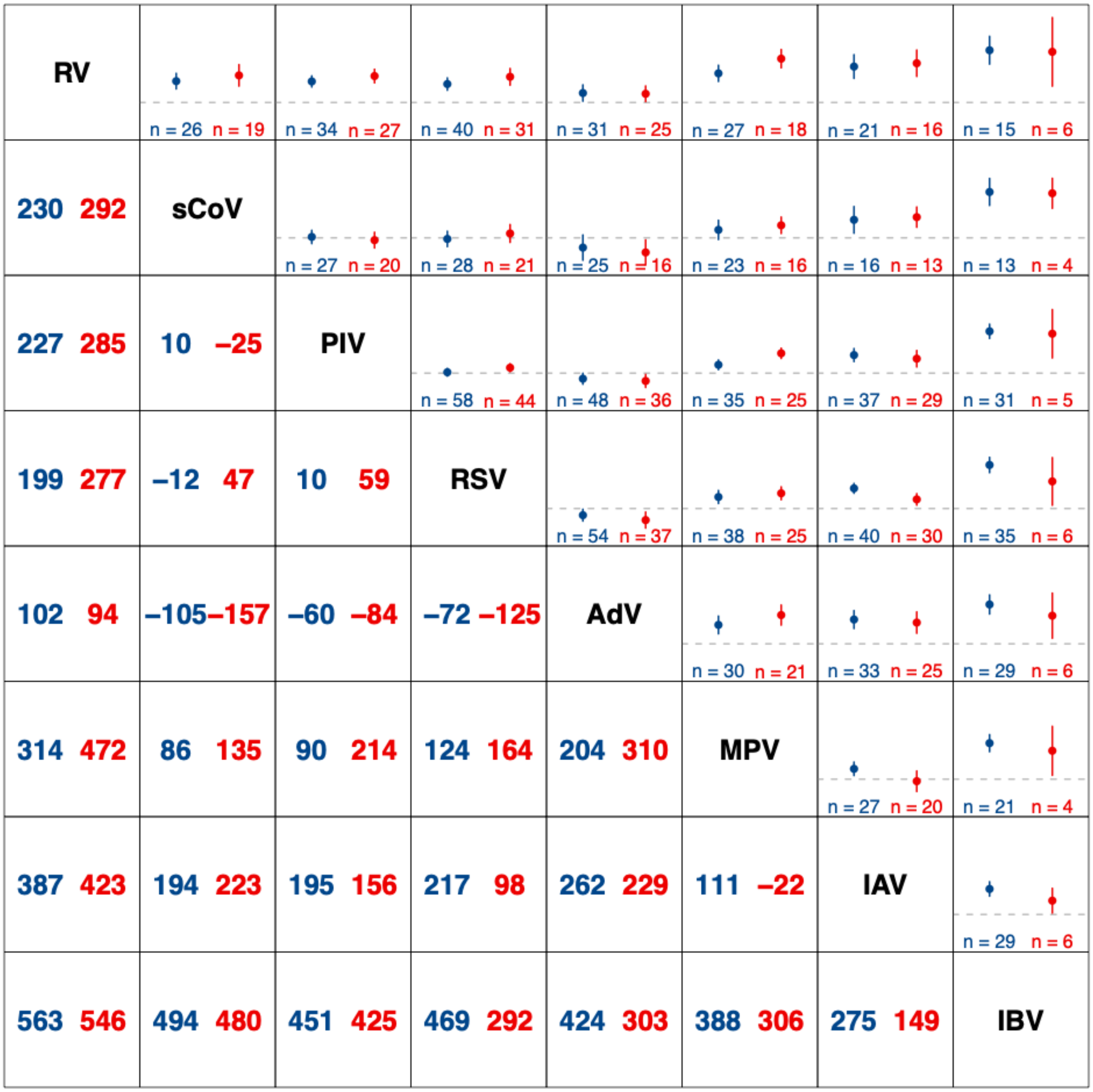


**Supplementary Figure 35. The time difference in days of peak between viruses during their first resurgence and second resurgence following the COVID-19 pandemic onset in study sites excluding Canadian data.**

The matrix presents the pairwise comparison of timing between viruses in their first (shown in blue) and second resurgence (shown in red). Positive [negative] values indicated that the virus on the top / left resurged earlier [later] than the virus on the bottom / right. Dashed line indicated no differences in the timing. Data are presented as mean values and error bars indicate their 95% confidence intervals. IAV = Influenza A virus; IBV = Influenza B virus; RSV = Respiratory Syncytial Virus; PIV = Parainfluenza Virus; MPV = Metapneumovirus; sCoV = Seasonal Coronavirus; RV = Rhinovirus; AdV = Adenovirus (AdV).

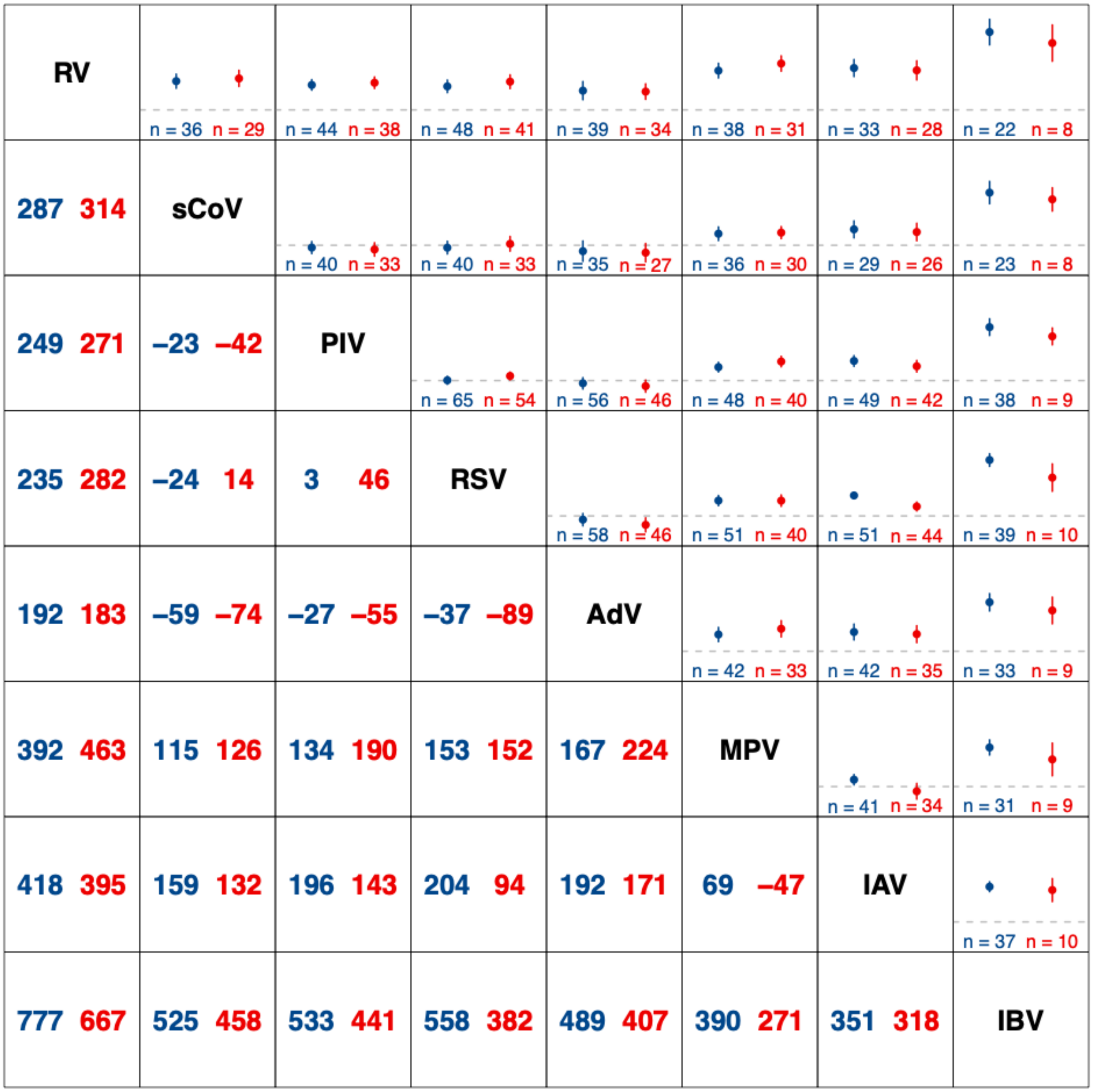


**Supplementary Figure 36. The time difference in days of onset between viruses during their first resurgence and second resurgence following the COVID-19 pandemic onset in study sites excluding Chinese data.**

The matrix presents the pairwise comparison of timing between viruses in their first (shown in blue) and second resurgence (shown in red). Positive [negative] values indicated that the virus on the top / left resurged earlier [later] than the virus on the bottom / right. Dashed line indicated no differences in the timing. Data are presented as mean values and error bars indicate their 95% confidence intervals. IAV = Influenza A virus; IBV = Influenza B virus; RSV = Respiratory Syncytial Virus; PIV = Parainfluenza Virus; MPV = Metapneumovirus; sCoV = Seasonal Coronavirus; RV = Rhinovirus; AdV = Adenovirus (AdV).

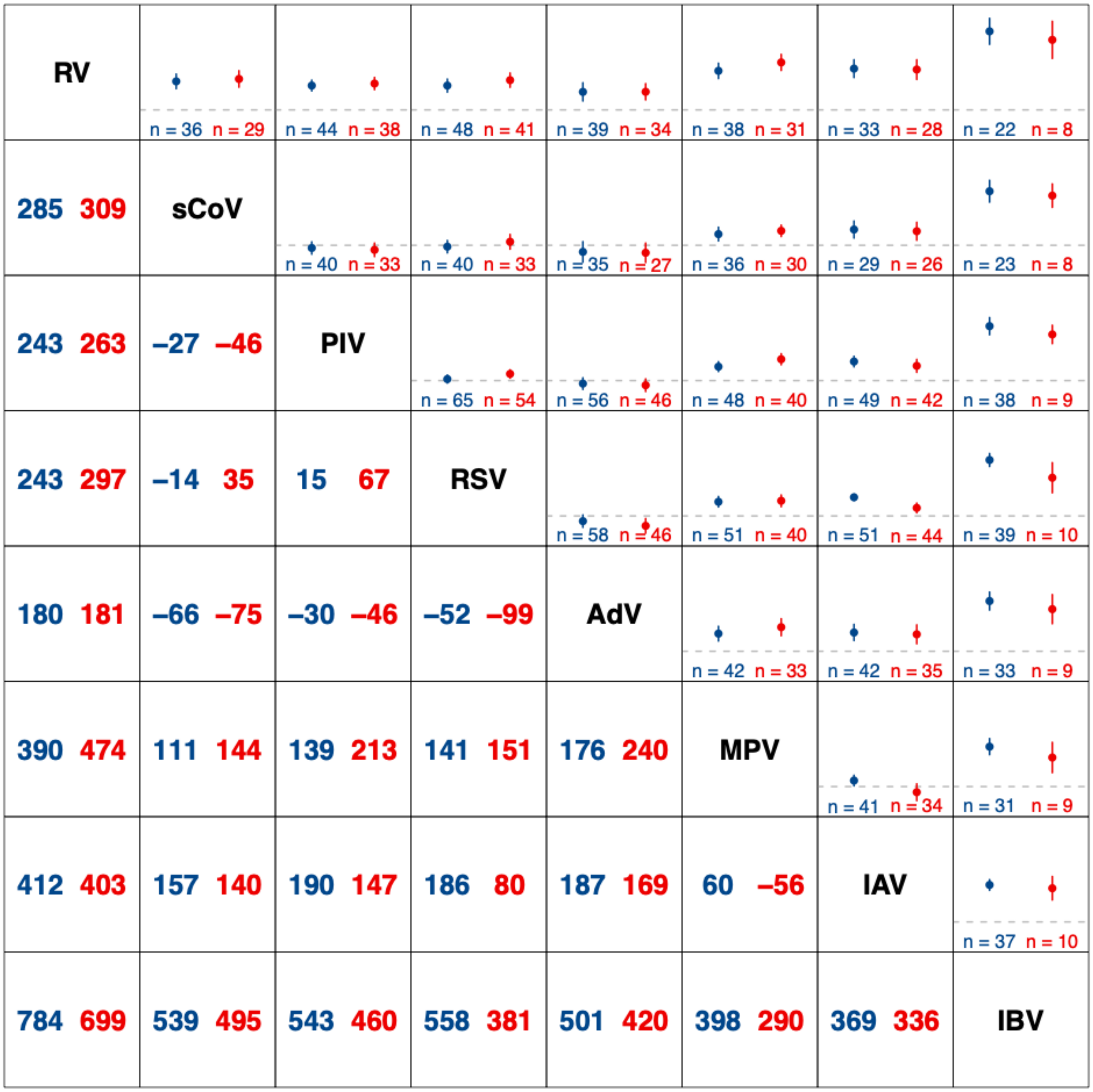

**Supplementary Figure 37. The time difference in days of peak between viruses during their first resurgence and second resurgence following the COVID-19 pandemic onset in study sites excluding Chinese data.**

The matrix presents the pairwise comparison of timing between viruses in their first (shown in blue) and second resurgence (shown in red). Positive [negative] values indicated that the virus on the top / left resurged earlier [later] than the virus on the bottom / right. Dashed line indicated no differences in the timing. Data are presented as mean values and error bars indicate their 95% confidence intervals. IAV = Influenza A virus; IBV = Influenza B virus; RSV = Respiratory Syncytial Virus; PIV = Parainfluenza Virus; MPV = Metapneumovirus; sCoV = Seasonal Coronavirus; RV = Rhinovirus; AdV = Adenovirus (AdV).

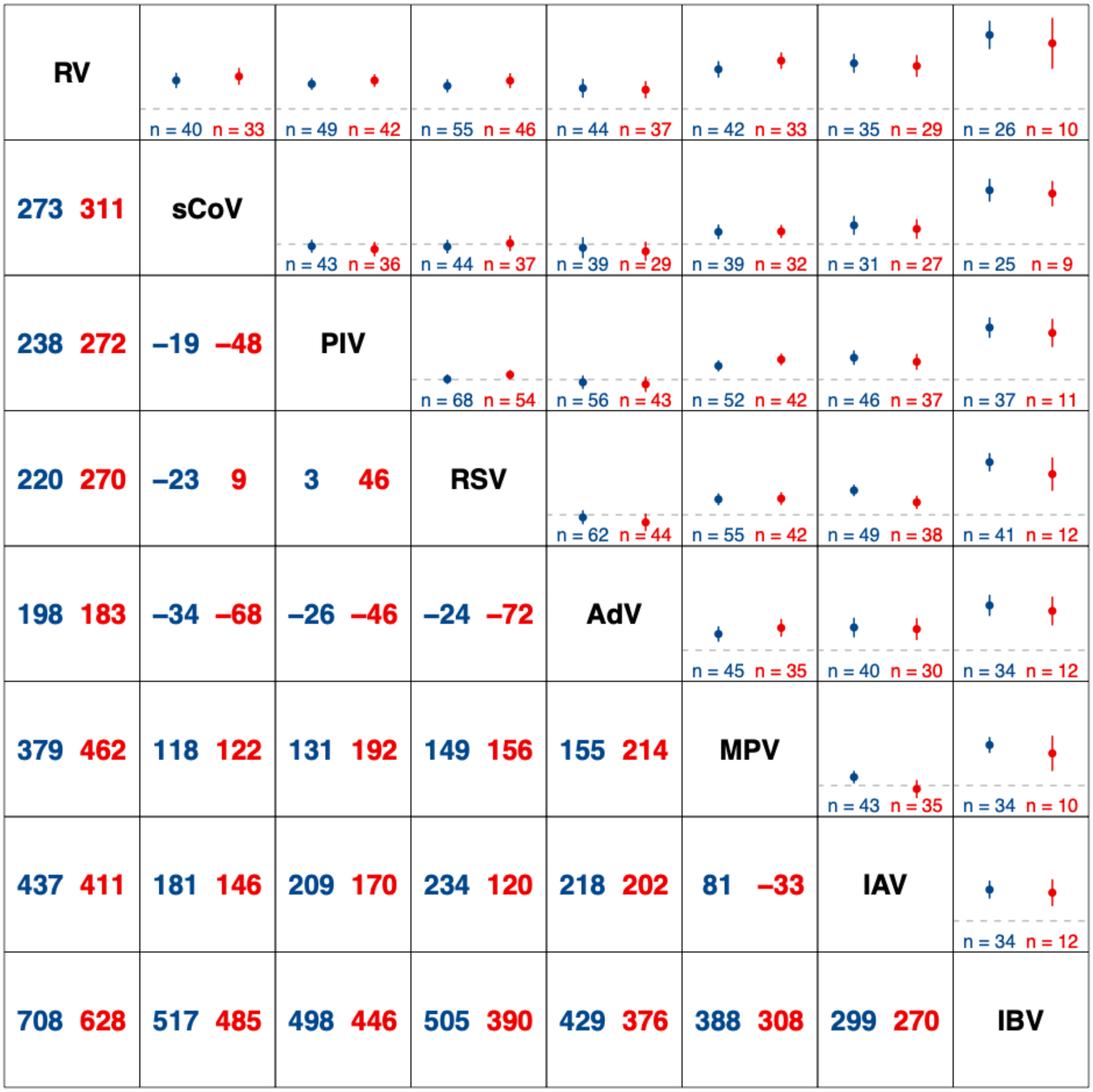


**Supplementary Figure 38. The time difference in days of onset between viruses during their first resurgence and second resurgence following the COVID-19 pandemic onset in study sites excluding Finnish data.**

The matrix presents the pairwise comparison of timing between viruses in their first (shown in blue) and second resurgence (shown in red). Positive [negative] values indicated that the virus on the top / left resurged earlier [later] than the virus on the bottom / right. Dashed line indicated no differences in the timing. Data are presented as mean values and error bars indicate their 95% confidence intervals. IAV = Influenza A virus; IBV = Influenza B virus; RSV = Respiratory Syncytial Virus; PIV = Parainfluenza Virus; MPV = Metapneumovirus; sCoV = Seasonal Coronavirus; RV = Rhinovirus; AdV = Adenovirus (AdV).

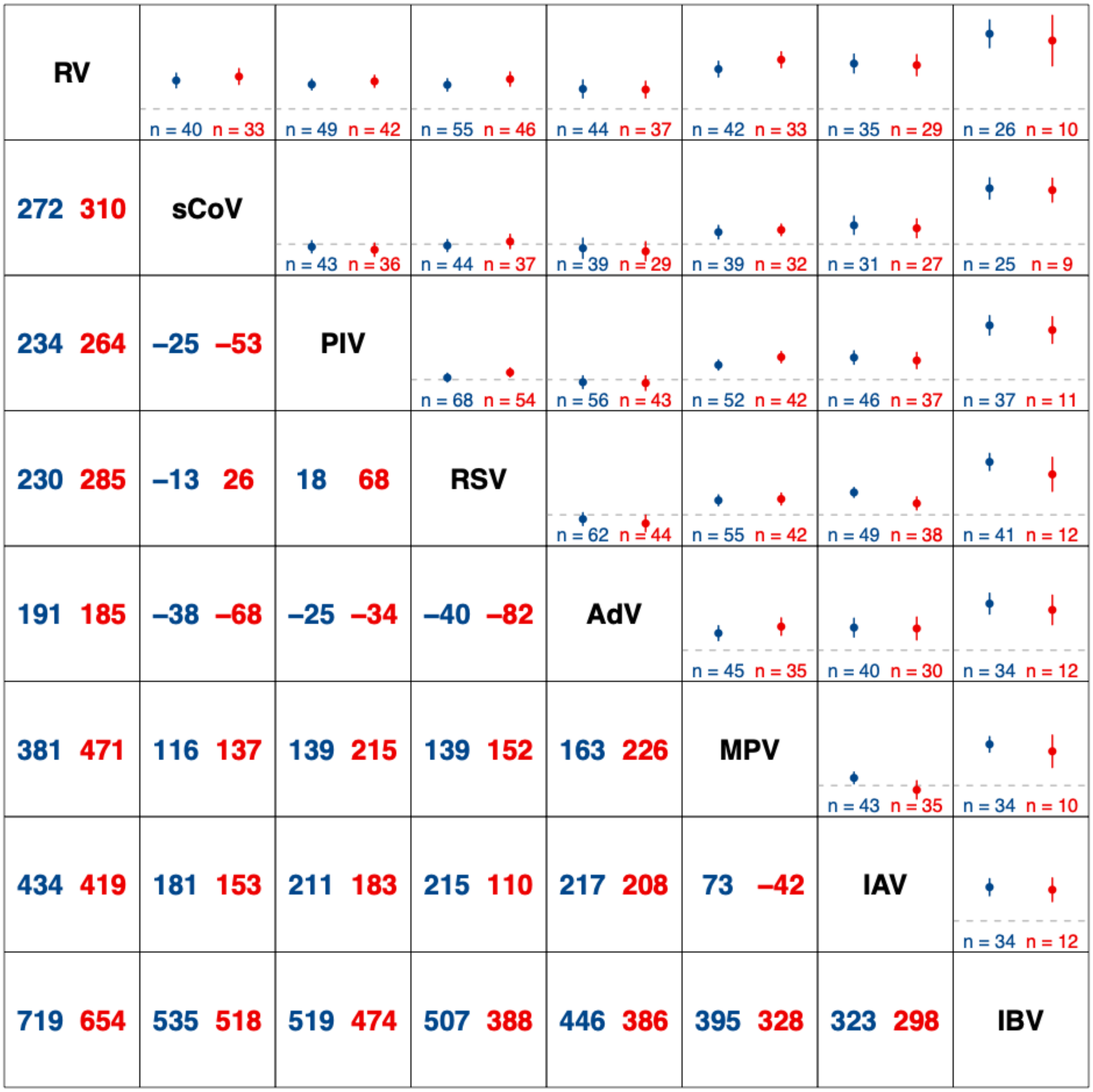


**Supplementary Figure 39. The time difference in days of peak between viruses during their first resurgence and second resurgence following the COVID-19 pandemic onset in study sites excluding Finnish data.**

The matrix presents the pairwise comparison of timing between viruses in their first (shown in blue) and second resurgence (shown in red). Positive [negative] values indicated that the virus on the top / left resurged earlier [later] than the virus on the bottom / right. Dashed line indicated no differences in the timing. Data are presented as mean values and error bars indicate their 95% confidence intervals. IAV = Influenza A virus; IBV = Influenza B virus; RSV = Respiratory Syncytial Virus; PIV = Parainfluenza Virus; MPV = Metapneumovirus; sCoV = Seasonal Coronavirus; RV = Rhinovirus; AdV = Adenovirus (AdV).

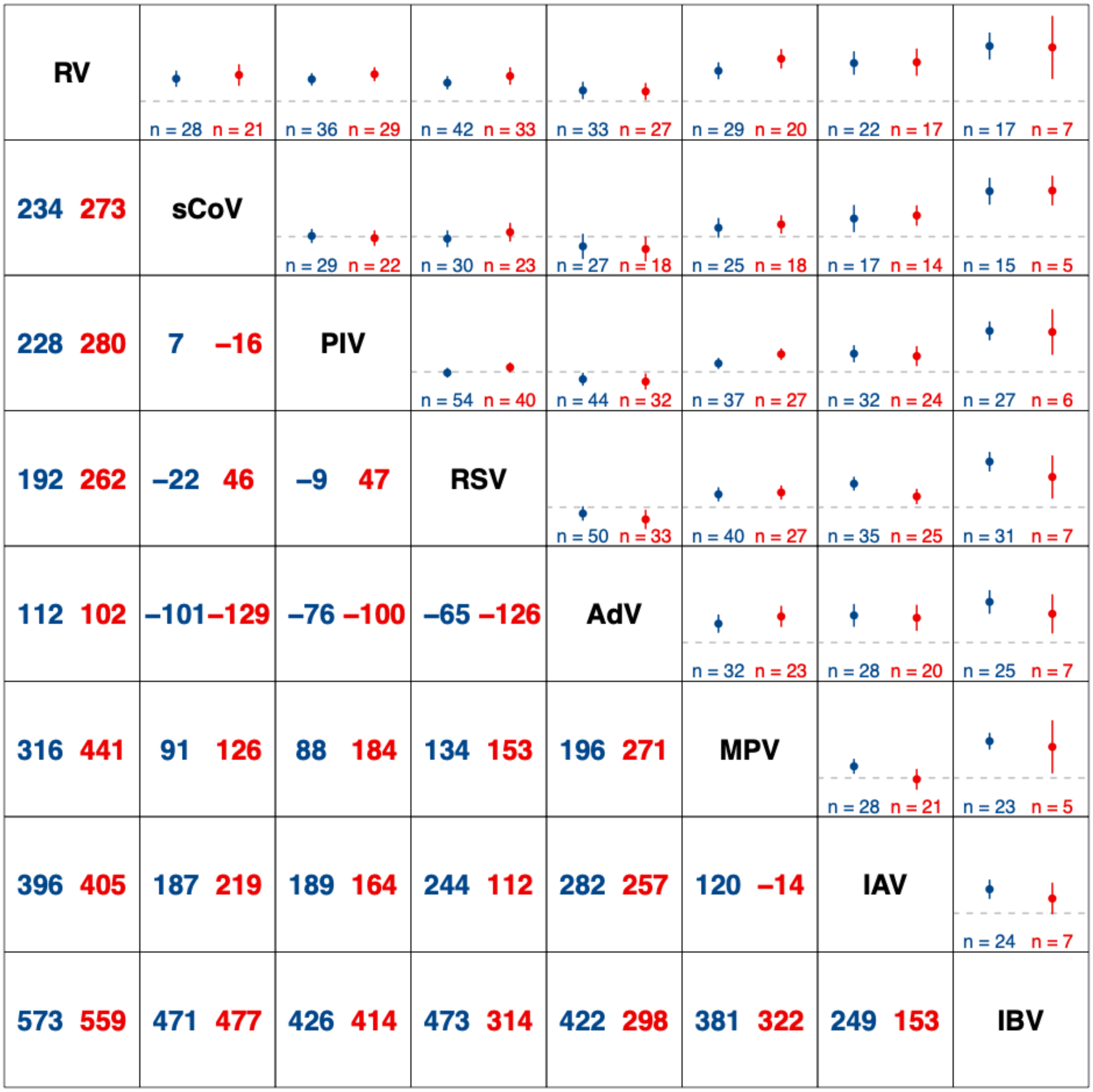


**Supplementary Figure 40. The time difference in days of onset between viruses during their first resurgence and second resurgence following the COVID-19 pandemic onset, using nationally aggregated data from Canada and Finland.**

The matrix presents the pairwise comparison of timing between viruses in their first (shown in blue) and second resurgence (shown in red). Positive [negative] values indicated that the virus on the top / left resurged earlier [later] than the virus on the bottom / right. Dashed line indicated no differences in the timing. Data are presented as mean values and error bars indicate their 95% confidence intervals. IAV = Influenza A virus; IBV = Influenza B virus; RSV = Respiratory Syncytial Virus; PIV = Parainfluenza Virus; MPV = Metapneumovirus; sCoV = Seasonal Coronavirus; RV = Rhinovirus; AdV = Adenovirus (AdV).

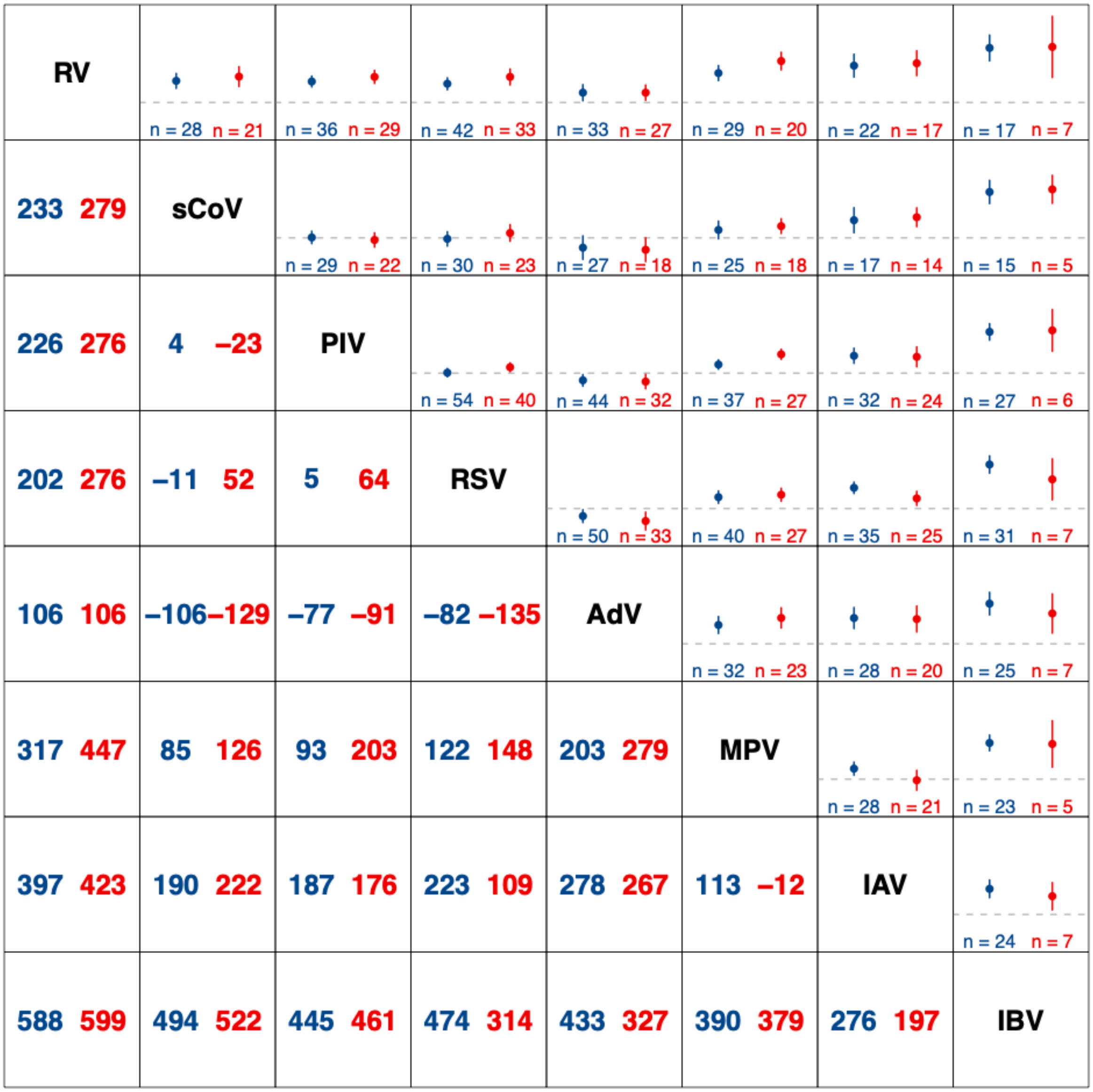


**Supplementary Figure 41. The time difference in days of peak between viruses during their first resurgence and second resurgence following the COVID-19 pandemic onset, using nationally aggregated data from Canada and Finland.**

The matrix presents the pairwise comparison of timing between viruses in their first (shown in blue) and second resurgence (shown in red). Positive [negative] values indicated that the virus on the top / left resurged earlier [later] than the virus on the bottom / right. Dashed line indicated no differences in the timing. Data are presented as mean values and error bars indicate their 95% confidence intervals. IAV = Influenza A virus; IBV = Influenza B virus; RSV = Respiratory Syncytial Virus; PIV = Parainfluenza Virus; MPV = Metapneumovirus; sCoV = Seasonal Coronavirus; RV = Rhinovirus; AdV = Adenovirus (AdV).

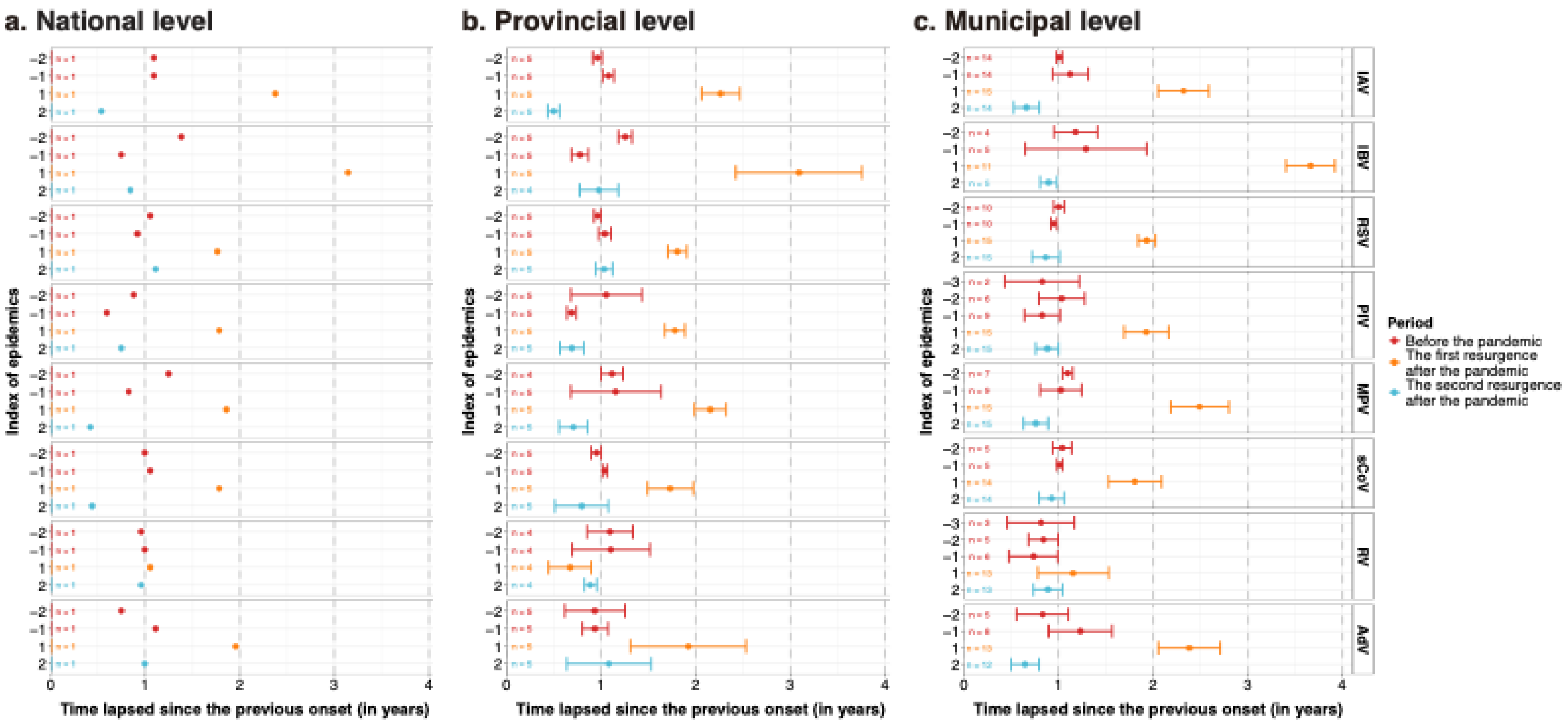


**Supplementary Figure 42. The virus-specific time interval (with 95% CI) between the onsets in two subsequent years across national (A), provincial (B), and municipal (C) levels in Canada.**

The x-axis represents the time lapsed since the previous onset, measured in years. The left y-axis lists the index of epidemics relative to the onset of the COVID-19 pandemic, with negative numbers indicating epidemics occurring before the pandemic, and positive numbers indicating epidemics occurring after the onset of the pandemic. Data are presented as mean values and error bars indicate their 95% confidence intervals. IAV = Influenza A virus; IBV = Influenza B virus; RSV = Respiratory Syncytial Virus; PIV = Parainfluenza Virus; MPV = Metapneumovirus; sCoV = Seasonal Coronavirus; RV = Rhinovirus; AdV = Adenovirus (AdV).

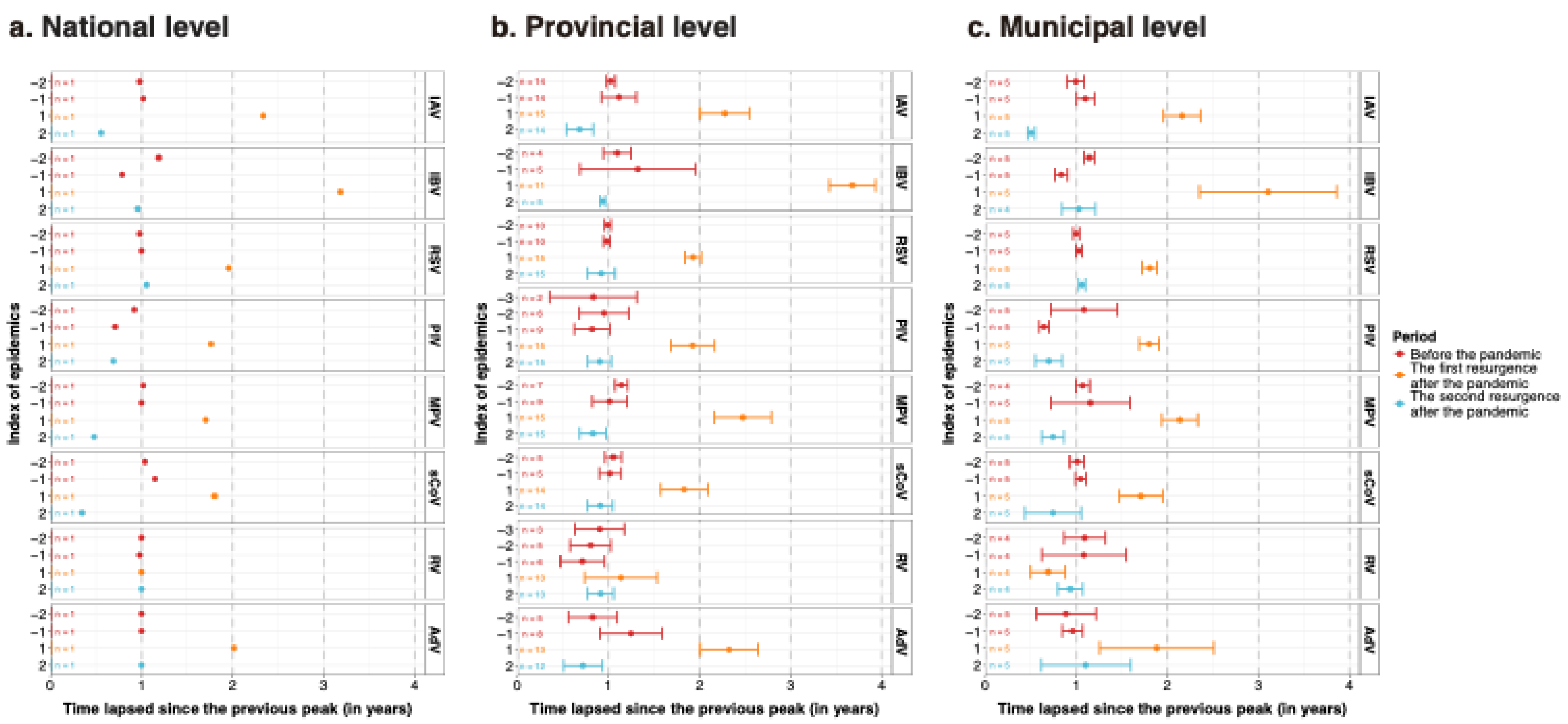


**Supplementary Figure 43. The virus-specific time interval (with 95% CI) between the peaks in two subsequent years across national (A), provincial (B), and municipal (C) levels in Canada.**

The x-axis represents the time lapsed since the previous onset, measured in years. The left y-axis lists the index of epidemics relative to the onset of the COVID-19 pandemic, with negative numbers indicating epidemics occurring before the pandemic, and positive numbers indicating epidemics occurring after the onset of the pandemic. Data are presented as mean values and error bars indicate their 95% confidence intervals. IAV = Influenza A virus; IBV = Influenza B virus; RSV = Respiratory Syncytial Virus; PIV = Parainfluenza Virus; MPV = Metapneumovirus; sCoV = Seasonal Coronavirus; RV = Rhinovirus; AdV = Adenovirus (AdV).

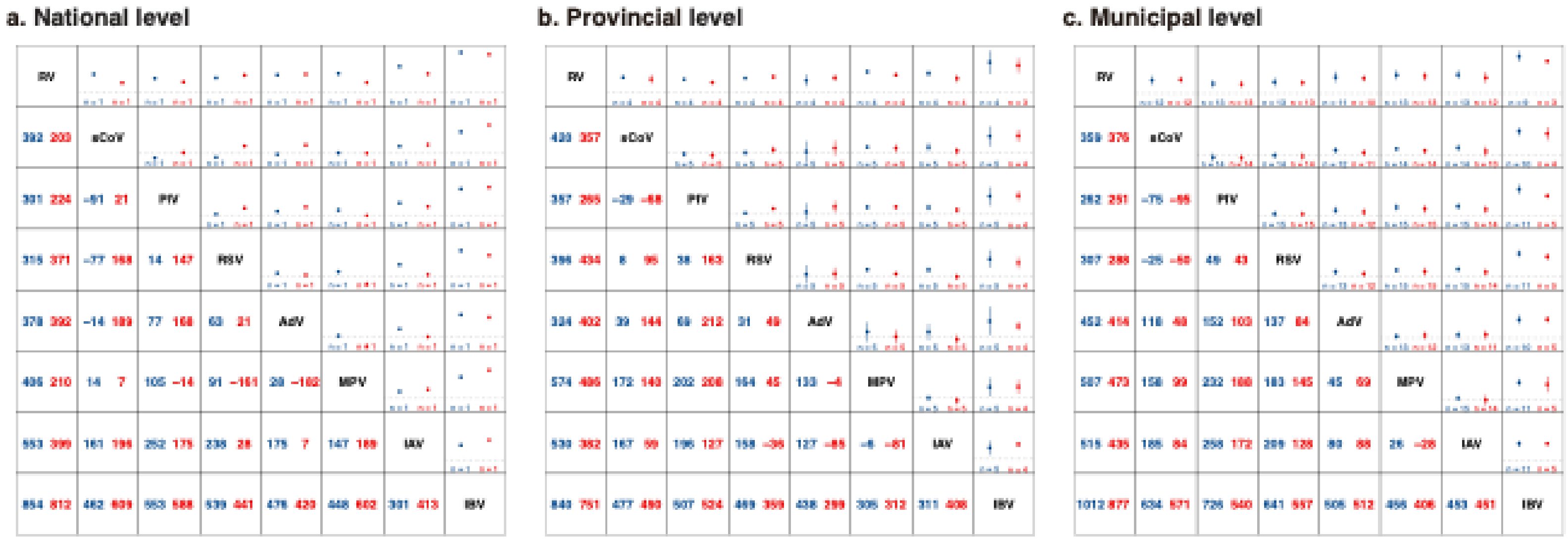

**Supplementary Figure 44. The time difference in days of onset between viruses during their first resurgence and second resurgence following the COVID-19 pandemic onset across national (A), provincial (B), and municipal (C) levels in Canada.**

The matrix presents the pairwise comparison of timing between viruses in their first (shown in blue) and second resurgence (shown in red). Positive [negative] values indicated that the virus on the top / left resurged earlier [later] than the virus on the bottom / right. Dashed line indicated no differences in the timing. Data are presented as mean values and error bars indicate their 95% confidence intervals. IAV = Influenza A virus; IBV = Influenza B virus; RSV = Respiratory Syncytial Virus; PIV = Parainfluenza Virus; MPV = Metapneumovirus; sCoV = Seasonal Coronavirus; RV = Rhinovirus; AdV = Adenovirus (AdV).

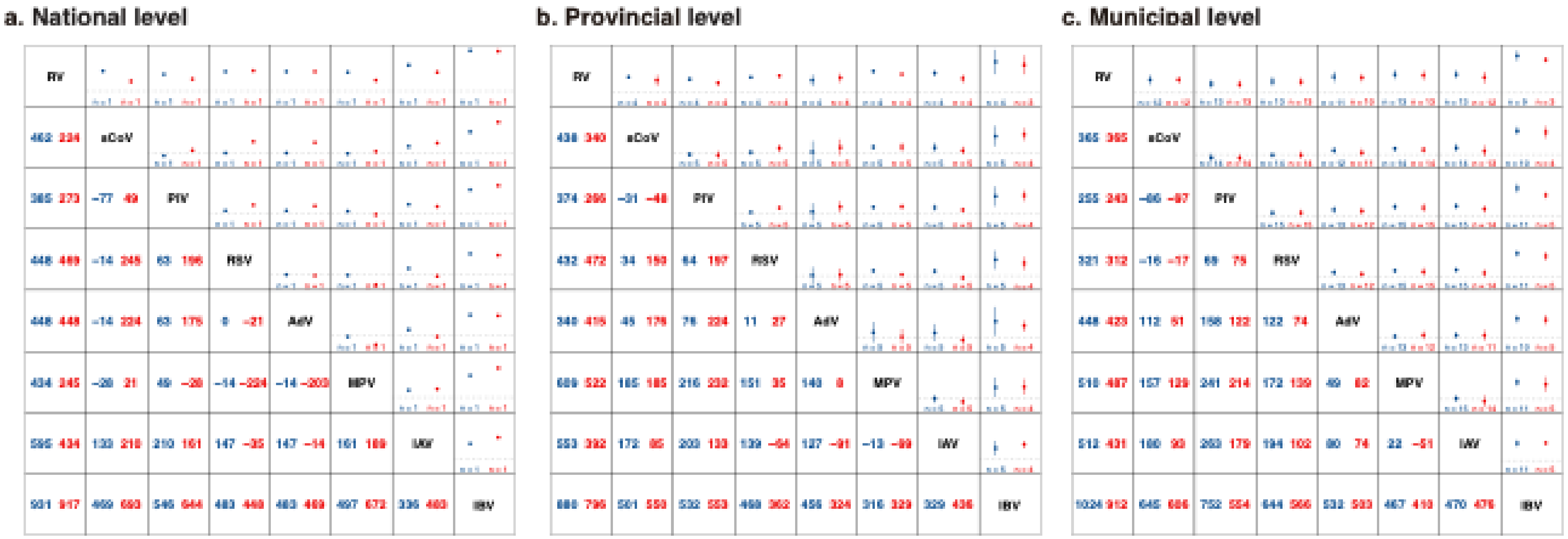

**Supplementary Figure 45. The time difference in days of peak between viruses during their first resurgence and second resurgence following the COVID-19 pandemic onset across national (A), provincial (B), and municipal (C) levels in Canada.**

The matrix presents the pairwise comparison of timing between viruses in their first (shown in blue) and second resurgence (shown in red). Positive [negative] values indicated that the virus on the top / left resurged earlier [later] than the virus on the bottom / right. Dashed line indicated no differences in the timing. Data are presented as mean values and error bars indicate their 95% confidence intervals. IAV = Influenza A virus; IBV = Influenza B virus; RSV = Respiratory Syncytial Virus; PIV = Parainfluenza Virus; MPV = Metapneumovirus; sCoV = Seasonal Coronavirus; RV = Rhinovirus; AdV = Adenovirus (AdV).

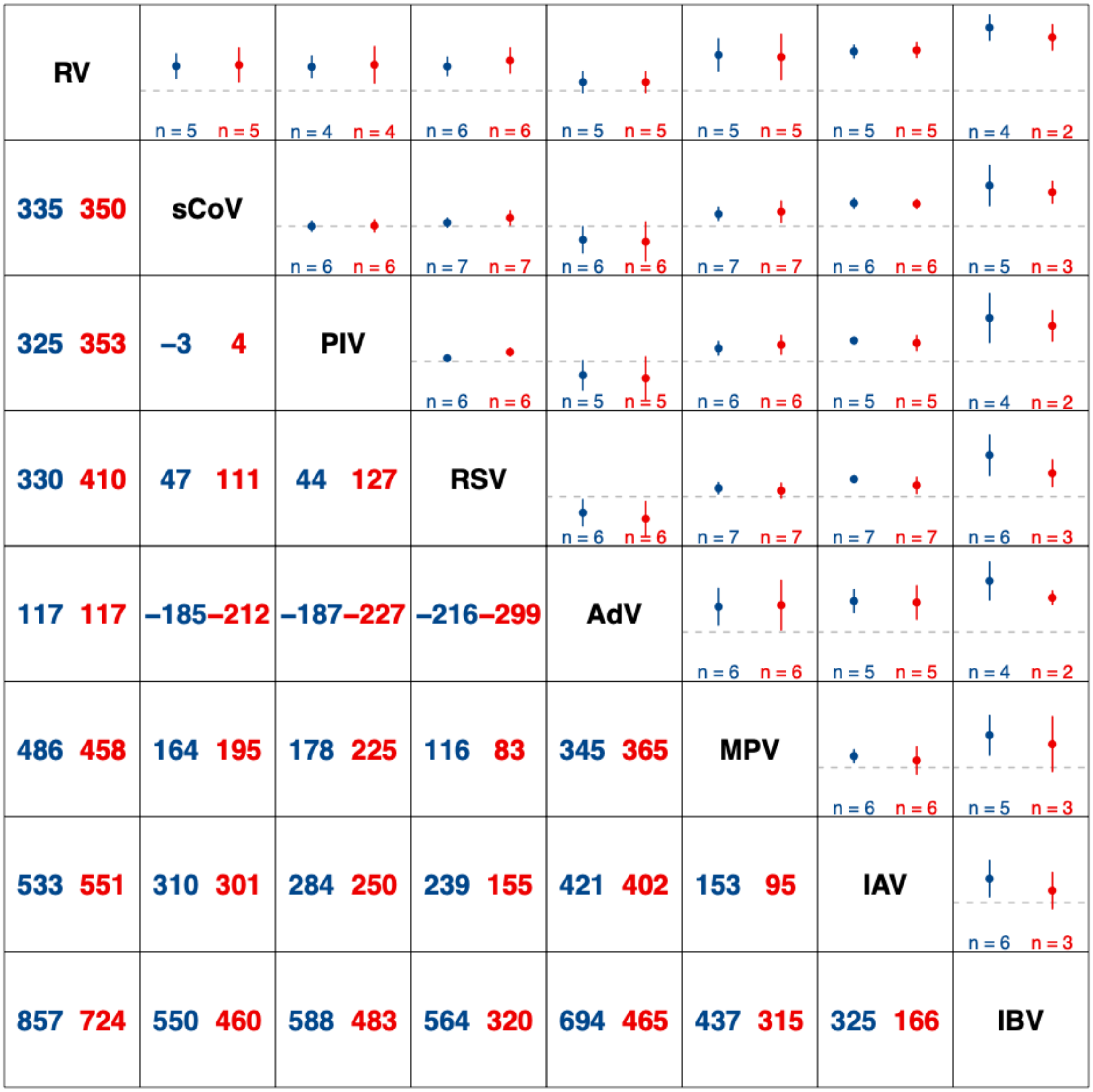


**Supplementary Figure 46. The time difference in days of onset between viruses during their first resurgence and second resurgence following the COVID-19 pandemic, matched by study sites that had over 1000 positive cases for each virus.**

The matrix presents the pairwise comparison of timing between viruses in their first (shown in blue) and second resurgence (shown in red). Positive [negative] values indicated that the virus on the top / left resurged earlier [later] than the virus on the bottom / right. Dashed line indicated no differences in the timing. Data are presented as mean values and error bars indicate their 95% confidence intervals. IAV = Influenza A virus; IBV = Influenza B virus; RSV = Respiratory Syncytial Virus; PIV = Parainfluenza Virus; MPV = Metapneumovirus; sCoV = Seasonal Coronavirus; RV = Rhinovirus; AdV = Adenovirus (AdV).

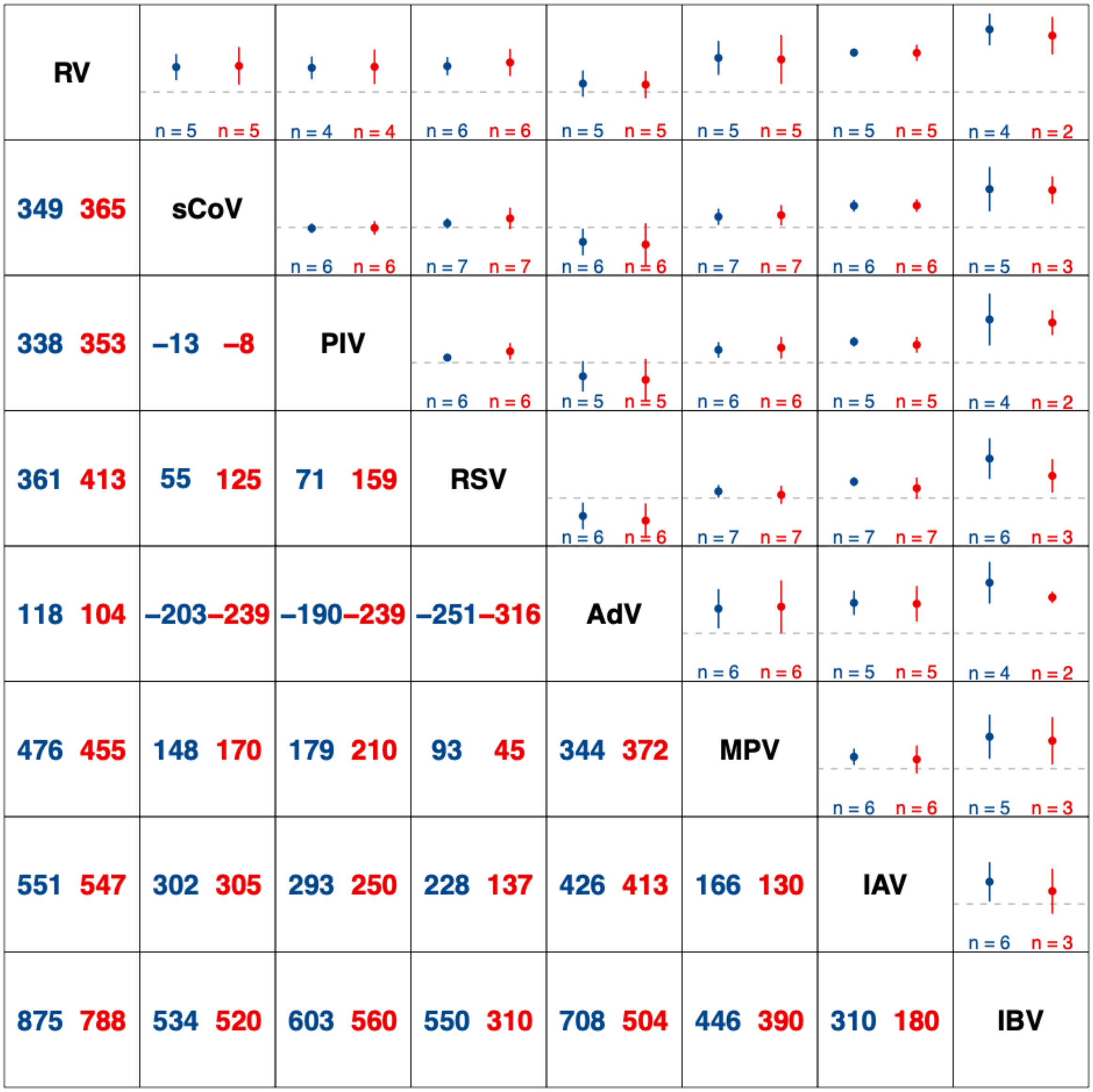


**Supplementary Figure 47.The time difference in days of peak between viruses during their first resurgence and second resurgence following the COVID-19 pandemic, matched by study sites that had over 1000 positive cases for each virus.**

The matrix presents the pairwise comparison of timing between viruses in their first (shown in blue) and second resurgence (shown in red). Positive [negative] values indicated that the virus on the top / left resurged earlier [later] than the virus on the bottom / right. Dashed line indicated no differences in the timing. Data are presented as mean values and error bars indicate their 95% confidence intervals. IAV = Influenza A virus; IBV = Influenza B virus; RSV = Respiratory Syncytial Virus; PIV = Parainfluenza Virus; MPV = Metapneumovirus; sCoV = Seasonal Coronavirus; RV = Rhinovirus; AdV = Adenovirus (AdV).

**Supplementary methods**

**Supplementary Methods 1. Search Strategy.**

**Web of science:**

1. TS=(respiratory viru* OR respiratory viral* OR respiratory NEAR/3 infectio* OR respiratory NEAR/1 disease* OR respiratory NEAR/1 illness*)
2. TS=(influenza* OR flu OR RSV OR Respiratory Syncytial Virus* OR parainfluenza* OR adenovirus* OR metapneumovirus* OR bocavirus* OR rhinovirus* OR enterovirus* OR IFV OR HPIV OR HAdV OR HMPV OR HCoV OR HBoV OR HRV)
3. TS=(nonpharmaceutical* OR ("non pharmaceutical*") OR NPI OR pandemic* OR COVID*)
4. TS=((epidemic* NEAR/1 characteristic*) OR (epidemic* NEAR/1 feature*) OR (epidemic* NEAR/1 trend) OR (epidemic* NEAR/1 change*) OR (epidemic* NEAR/1 pattern*))
5. TS=((prevalence NEAR/1 characteristic*) OR (prevalence NEAR/1 feature*) OR (prevalence NEAR/1 trend) OR (prevalence NEAR/1 change*) OR (prevalence NEAR/1 pattern*))
6. TS=((contagio* NEAR/2 characteristic*) OR (contagio* NEAR/2 feature*) OR (contagio* NEAR/2 trend) OR (contagio* NEAR/2 change*) OR (contagio* NEAR/2 pattern*))
7. TS=((circulat* NEAR/1 characteristic*) OR (circulat* NEAR/1 feature*) OR (circulat* NEAR/1 trend) OR (circulat* NEAR/1 change*) OR (circulat* NEAR/1 pattern*))
8. TS=((viru* NEAR/2 activity) OR (viru* NEAR/2 trend) OR (viral* NEAR/2 activity) OR (viral* NEAR/2 trend))
9. TS=(hospitalization* OR (surveillance NEAR/1 data))
10. (#4) OR (#5) OR (#6) OR (#7) OR (#8) OR (#9)
11. (#1) AND (#2) AND (#3) AND (#10)
12. (#11) AND PY=(2021-2023)

**Medline：**

1. exp Respiratory Tract Infections/ep, pc, sn, tm or (respiratory adj3 infectio*).mp. or exp Respirovirus/ or Respirovirus Infections/ep, pc, tm or Respirovirus/ or (respiratory viru*).mp. or (respiratory viral*).mp. or exp Respiratory Tract Diseases/ep, pc, tm or (respiratory adj1 disease*).mp. or (respiratory adj1 illness*).mp.
2. Pandemic/ or pandemic.mp. or COVID-19/ep, pc or nonpharmaceutical*.mp. or (non adj1 pharmaceutical*).mp.
3. Influenza, Human/ep, pc, sn, tm or influenza*.mp. or flu.mp. or IFV.mp. or Respiratory Syncytial Virus, Human/ or Respiratory Syncytial Virus* or RSV.mp. or exp Rubulavirus/ or parainfluenza* or HPIV.mp. or Metapneumovirus/ or metapneumovirus* or HMPV.mp. or Human bocavirus/ or bocavirus* or HBoV.mp. or Rhinovirus/ or rhinovirus* or HRV.mp. or exp Enterovirus/ or enterovirus* or exp Coronavirus/ or HCoV.mp. or exp Adenoviruses, Human/ or adenovirus* or HAdv.mp.
4. exp Epidemiology/
5. exp Population Surveillance/
6. exp Incidence/
7. exp Prevalence/
8. exp Hospitalization/

9. ((epidemi* or prevalence or contagious or circulat* or viru* or viral*) adj3 (characteristic* or feature* or trend or change* or pattern* or activity or hospitalization*)).mp.
10. surveillance data.mp.
11. 1 and 2 and 3
12. 4 or 5 or 6 or 7 or 8 or 9 or 10
13. 11 and 12
14. limit 13 to (humans and yr="2021 -Current")

**Embase:**

1. 'Respiratory Tract Infections'/exp OR 'Respirovirus'/exp OR 'Respirovirus infection'/exp OR (respiratory NEXT/1 (viru* or viral* or infectio* or disease* or illness*))
2. 'pandemic'/de OR pandemic* OR 'coronavirus disease 2019'/de OR 'nonpharmaceutical*' OR 'non NEXT/1 pharmaceutical*'
3. 'influenza'/exp OR influenza* OR IFV OR flu OR 'human respiratory syncytial virus'/exp OR 'Respiratory Syncytial Virus*' OR RSV OR 'rubulavirus'/exp OR 'Parainfluenza virus infection'/exp OR parainfluenza* OR HPIV.mp. OR 'metapneumovirus'/exp OR metapneumovirus* OR HMPV OR 'bocaparvovirus'/exp OR bocavirus* OR HBoV OR 'rhinovirus'/exp OR rhinovirus* OR HRV OR 'enterovirus'/exp OR enterovirus* OR 'coronavirinae'/exp OR HCoV OR 'Human adenovirus C'/exp OR adenovirus* OR HAdV
4. 'epidemic'/exp OR 'epidemiological surveillance'/exp OR 'incidence'/exp OR 'infection rate'/exp OR 'morbidity'/exp OR 'prevalence'/exp OR 'hospitalization'/exp OR 'seasonal variation'/exp OR ((epidemi* OR prevalence OR contagious OR circulat* OR viru* OR viral*) NEXT/1 (characteristic* OR feature* OR trend OR change* OR pattern* OR activity OR hospitalization*)) OR 'surveillance data'
5. #1 AND #2 AND #3 AND #4
6. #5 AND (2021:py OR 2022:py OR 2023:py) AND 'human'/de

**WHO COVID-19 Research Database:**

1. ((respiratory viru*) OR (respiratory viral*) OR (respiratory infectio*) OR (respiratory disease*) OR (respiratory illness*))
2. (influenza* OR flu OR RSV OR (Respiratory Syncytial Virus*) OR parainfluenza* OR adenovirus* OR metapneumovirus* OR bocavirus* OR rhinovirus* OR enterovirus* OR IFV OR HPIV OR HAdV OR HMPV OR HCoV OR HBoV OR HRV)
3. (nonpharmaceutical* OR (non pharmaceutical*) OR NPI OR pandemic* OR COVID*)
4. ((epidemic* characteristic*) OR (epidemic* feature*) OR (epidemic* trend) OR (epidemic* change*) OR (epidemic* pattern*))
5. ((prevalence characteristic*) OR (prevalence feature*) OR (prevalence trend) OR (prevalence change*) OR (prevalence pattern*))
6. ((contagio* characteristic*) OR (contagio* feature*) OR (contagio* trend) OR (contagio* change*) OR (contagio* pattern*))
7. ((circulat* characteristic*) OR (circulat* feature*) OR (circulat* trend) OR (circulat* change*) OR (circulat*

pattern*))

8. ((viru* activity) OR (viru* trend) OR (viral* activity) OR (viral* trend)) OR
9. (hospitalization* OR (surveillance data))
10. 4 OR 5 OR 6 OR 7 OR 8 OR 9
11. 1 AND 2 AND 3 AND 10
12. 11 AND AND year_cluster:("2021" OR "2022" OR "2023")
13. 12 AND db:("MEDLINE" OR "EuropePMC" OR "EMBASE" OR "ICTRP" OR "Scopus" OR "COVIDWHO" OR "LILACS" OR "Web of Science" OR "CAB Abstracts" OR "ProQuest Central" OR "Indonesian Research" OR "MDPI" OR "Academic Search Complete" OR "ScienceDirect" OR "GIM" OR "PubMed")

**Supplementary Methods 2. Quality assessment form.**

| **Category** | **Question** | **Assessment**<br>**Yes – 1 point.**<br>**No – 0 points.**<br>**Not clear – 0 points.** |
|---|---|---|
| Representativeness | Q1: Whether the study was a multicenter study? | |
| Representativeness | Q2: Whether the study included all age groups? | |
| Precision | Q3: Whether the viral activities were reported on a weekly basis? | |
| Precision | Q4: Whether the total number of positive cases for all viruses exceeded 1,000 since 2020? | |
| Reliability | Q5: For published literatures: Whether the viral activity data were not extracted from figures?<br>For other sources: Whether data were missing for ≤ 3 months? | |
| Reliability | Q6: Whether the testing method of different viruses were consistent? | |
| Representativeness | Q7: Whether viral activity data were available for at least five types of viruses? | |
| Reliability | Q8: Whether the testing capacity was stable during the study period? * | |

* We evaluated testing stability by two distinct criteria. First, whether the data source had any documented major interruptions in testing during the pandemic. Second, whether the number of specimens taken was consistently zero for more than two weeks.

**Reference**


1. Lumley, S. F. *et al.* Changes in paediatric respiratory infections at a UK teaching hospital 2016-2021; impact of the SARS-CoV-2 pandemic. *J Infect* **84**, 40–47 (2022).
2. Yohei Kume *et al.* Changes in virus detection in hospitalized children before and after the severe acute respiratory syndrome coronavirus 2 pandemic. *Influenza other respir. viruses* **16**, 837–841 (2022).
3. Shi, H. J. *et al.* Effects of Non-Pharmacological Interventions on Respiratory Viruses Other Than SARS-CoV-2: Analysis of Laboratory Surveillance and Literature Review From 2018 to 2021. *J Korean Med Sci* **37**, e172 (2022).
4. Han, X., Xu, P., Wang, H., Mao, J. & Ye, Q. Incident changes in the prevalence of respiratory virus among children during COVID-19 pandemic in Hangzhou, China. *J. Infect.* **84**, 579–613 (2022).
5. Sominina, A. A. *et al.* Interference of sars-cov-2 with other respiratory viral infections agents during pandemic. *Epidemiologiya i Vaktsinoprofilaktika* **20**, 28–39 (2021).
6. Liu, P. *et al.* The changing pattern of common respiratory and enteric viruses among outpatient children in Shanghai, China: Two years of the COVID-19 pandemic. *J Med Virol* **94**, 4696–4703 (2022).
7. Hanchi, A. L. *et al.* Changes in the Epidemiology of Respiratory Pathogens in Children during the COVID-19 Pandemic. *Pathogens* **11**, (2022).
8. Park, C. *et al.* Changes in the pattern and disease burden of acute respiratory viral infections before and during the COVID-19 pandemic. *Osong Public Health and Research Perspectives* **13**, 203–211 (2022).
9. Wan, L. *et al.* The changing pattern of common respiratory viruses among children from 2018 to 2021 in Wuhan, China. *Arch Virol* **168**, 291 (2023).
10. Dagan, R. *et al.* The COVID-19 Pandemic as an Opportunity for Unravelling the Causative Association between Respiratory Viruses and Pneumococcus-Associated Disease in Young Children: A Prospective Study. *Lancet eBioMedicine* (2022) doi:10/gtqwvx.
11. Razanajatovo, N. H. *et al.* Epidemiological Patterns of Seasonal Respiratory Viruses during the COVID-19 Pandemic in Madagascar, March 2020-May 2022. *Viruses* **15**, 12–12 (2023).
12. Danino, D. *et al.* Involvement of Respiratory Viruses in Community-Acquired Alveolar Pneumonia (CAAP) in Children < 5 Years in Southern Israel, Before and During the COVID-19 Pandemic. *Open Forum Infectious Diseases* **9(Supplement 2)**, S896–S897 (2022).
13. Wang, H. *et al.* Lockdown measures during the COVID-19 pandemic strongly impacted the circulation of respiratory pathogens in Southern China. *Sci Rep* **12**, 16926–16926 (2022).
14. Fratty, I. S. *et al.* Outbreak of Influenza and Other Respiratory Viruses in Hospitalized Patients Alongside the SARS-CoV-2 Pandemic. *Frontiers in Microbiology* **13**, (2022).
15. Haddadin, Z. *et al.* Respiratory pathogens during the COVID-19 pandemic: Alterations in detection and seasonality in Nashville, Tennessee. *PLoS ONE* **17**, e0270469–e0270469 (2022).
16. Oster, Y. *et al.* Viral and Bacterial Respiratory Pathogens during the COVID-19 Pandemic in Israel. *MICROORGANISMS* **11**, 166–166 (2023).
17. Li, Y. *et al.* Prevalence of respiratory viruses among hospitalized children with lower respiratory tract infections during the COVID-19 pandemic in Wuhan, China. *International Journal of Infectious Diseases* **139**, 6–12 (2024).

18. Lin, C. X. *et al.* Comparison of 14 respiratory pathogens among hospitalized children during and after the COVID-19 outbreak in Chaoshan area. *Virology Journal* **20**, (2023).
19. Feng, Y., Zhang, H., Zhang, B., Zhou, Y. & Yuan, H. Impact of normalized COVID-19 prevention and control measures on lower respiratory tract infection pathogenesis in hospitalized children. *Front Public Health* **12**, 1367614 (2024).
20. Maison, N. *et al.* Old foes following news ways?-Pandemic-related changes in the epidemiology of viral respiratory tract infections. *Infection* **52**, 209–218 (2024).
21. Fu, X. *et al.* Etiological characteristics of acute respiratory infections during the SARS-CoV-2 epidemic in Guizhou Province, China. *Journal of International Medical Research* **52**, 3000605241236050 (2024).
22. Fukuda, Y. *et al.* Changing patterns of infectious diseases among hospitalized children in hokkaido, japan, in the post-COVID-19 era, july 2019 to june 2022. *Pediatr. Infect. Dis. J.* **42**, 766–773 (2023).
23. Cao, R. *et al.* Influence of COVID-19 pandemic on the virus spectrum in children with respiratory infection in Xuzhou, China: a long-term active surveillance study from 2015 to 2021. *BMC Infect. Dis.* **23**, 467 (2023).
24. Guadalupe-Fernandez, V. *et al.* Investigating epidemiological distribution (temporality and intensity) of respiratory pathogens following COVID-19 de-escalation process in Catalonia, September 2016-June 2021: Analysis of regional surveillance data. *PLoS ONE [Electronic Resource]* **19**, e0285892 (2024).
25. Hao, Y., Cheng, L. & Lu, D. Epidemiological Study of Respiratory Virus Infections among Hospitalized Children Aged 14 Years and Younger during COVID-19 Pandemic in Wuhan, China, 2018–2022. *J Glob Infect Dis* **15**, 149–155 (2023).
26. Tang, M. *et al.* Comparison of respiratory pathogens in children with community-acquired pneumonia before and during the COVID-19 pandemic. *BMC Pediatrics* **23**, (2023).
27. Xia, J. *et al.* Respiratory virus infections among hospitalized children aged 7 years and younger in Wuhan, China, 2017–2021. *Journal of Infection* **86**, e1–e4 (2023).
28. Michaela Davids *et al.* Changes in Prevalence and Seasonality of Pathogens Identified in Acute Respiratory Tract Infections in Hospitalised Individuals in Rural and Urban Settings in South Africa; 2018-2022. *Viruses* **16**, 05 (2024).
29. Dina, J. *et al.* Characteristics of respiratory viruses' circulation through a six-year period (2016–2022) in a pediatric population in Normandy, France, and the impact of COVID-19 pandemic. *Microbiology Spectrum* **11**, (2023).
30. Kırca, F. *et al.* Impact of non-pharmaceutical interventions on circulating respiratory viruses during the COVID-19 pandemic in Turkey. *Annals of Saudi Medicine* **43**, 143–153 (2023).
31. Chan, W.-S. *et al.* The Seasonality of Respiratory Viruses in a Hong Kong Hospital, 2014–2023. *Viruses* **15**, (2023).
32. Centers for Disease Control and Prevention. National Respiratory and Enteric Virus Surveillance System. https://www.cdc.gov/surveillance/nrevss/index.html (2023).
33. Seattle Flu Alliance. Seattle Flu Study. *Pathogens* https://seattleflu.org/pathogens.
34. Houston Methodist. Respiratory Pathogen Epidemiology Snapshot. https://flu.houstonmethodist.org/#.
35. KCDC Infectious Disease Homepage. https://npt.kdca.go.kr/npt/biz/npp/iss/ariStatisticsMain.do.

36. Finnish institute for Health and Welfare. Finnish National Infectious Diseases Register. https://www.thl.fi/ttr/gen/rpt/tilastot.html.
37. Taiwan National Infectious Disease Statistics System. https://nidss.cdc.gov.tw/en/Home/Index?op=2.
38. Státní zdravotní ústav. Zprávy o chřipkové aktivitě, hlášení a výsledky laboratorních vyšetření. *SZÚ | Oficiální web Státního zdravotního ústavu v Praze* https://szu.cz/tema/a-z-infekce/ch/chripka/zprava-o-chripkove-aktivite-hlaseni-a-vysledky-laboratornich-vysetreni/.
39. Canada, P. H. A. of. Respiratory Virus Detections in Canada. https://www.canada.ca/en/public-health/services/surveillance/respiratory-virus-detections-canada.html (2012).
40. FluNet. PAHO - Epidemic diseases - Flu. https://ais.paho.org/phip/viz/ed_flu.asp.
41. UKHSA. Weekly national flu reports. *GOV.UK* https://www.gov.uk/government/collections/weekly-national-flu-reports (2023).
42. Public Health Scotland. Scottish Health and Social Care Open Data. https://www.opendata.nhs.scot/dataset/viral-respiratory-diseases-including-influenza-and-covid-19-data-in-scotland/resource/5032c2b9-a206-4024-9536-5fdf345c8483.
43. Robert Koch Institut. Arbeitsgemeinschaft Influenza. https://influenza.rki.de/Wochenberichte.aspx.
44. Cong, B. *et al.* Changes in the global hospitalisation burden of respiratory syncytial virus in young children during the COVID-19 pandemic: a systematic analysis. *Lancet Infect Dis* S1473-3099(23)00630–8 (2023) doi:10.1016/S1473-3099(23)00630-8.
